# Structural Bifurcation in the High→Low‐Spin and Low→High‐Spin Phase Transitions Explains the Asymmetric Spin‐Crossover in [FeL_2_][BF_4_]_2_ (L=2,6‐Di{pyrazol‐1‐yl}isonicotinonitrile)

**DOI:** 10.1002/anie.202416924

**Published:** 2024-12-17

**Authors:** Ahmed Ahmed, Amy Hall, Hari Babu Vasili, Rafal Kulmaczewski, Alexander N. Kulak, Oscar Cespedes, Christopher M. Pask, Lee Brammer, Thomas M. Roseveare, Malcolm A. Halcrow

**Affiliations:** ^1^ School of Chemistry University of Leeds Woodhouse Lane Leeds UK LS2 9JT; ^2^ School of Natural Sciences College of Science and Engineering University of Galway H91 TK 33 Galway Ireland; ^3^ School of Physics and Astronomy University of Leeds W. H. Bragg Building Leeds UK LS2 9JT; ^4^ Department of Chemistry University of Sheffield Brook Hill Sheffield UK S3 7HF

**Keywords:** iron, spin-crossover, crystal engineering, phase transitions, in situ diffraction

## Abstract

Polycrystalline [FeL_2_][BF_4_]_2_ (L=2,6‐di(pyrazol‐1‐yl)isonicotinonitrile) exhibits an abrupt hysteretic spin transition near 240 K, with a shoulder on the warming branch whose appearance depends on the sample history. The freshly isolated material is a *ca* 60 : 40 mixture of triclinic (HS1) and tetragonal (HS2) high‐spin polymorphs, which are structurally closely related. Both HS1 and HS2 undergo a high→low‐spin transition on cooling at 230±10 K. HS1 transforms to a new triclinic low‐spin phase with a doubled unit cell volume (LS3), while HS2 forms a monoclinic low‐spin phase (LS4) with similar unit cell dimensions to HS2. Single crystals of LS3 and LS4 both convert to HS1 on rewarming. The low→high‐spin transition for LS4 is *ca* 10 K higher in temperature than for LS3, explaining the asymmetric thermal hysteresis. Powder diffraction, calorimetry and magnetic data show that multiple cycling about the spin‐transition leads to slow enrichment of the HS1 and LS3 phases at the expense of HS2 and LS4. That is consistent with the HS2/LS4 fraction of the polycrystalline sample undergoing rare, bifurcated HS2→(LS3+LS4) and LS4→(HS1+HS2) phase transitions. The rate of enrichment of HS1/LS3 differed between these experiments, implying it is sample and/or measurement‐dependent. Three other salts of this iron(II) complex and the coordination polymer [Ag(*μ*‐L)]BF_4_ are also briefly described.

## Graphical abstract

The title compound exhibits a cooperative spin‐transition with an unusual asymmetric hysteresis. It crystallizes as a mixture of two high‐spin polymorphs HS1 and HS2, which convert simultaneously to the low‐spin phases LS3 and LS4 on cooling. The HS1 and LS3 fractions increase at the expense of HS2 and LS4 upon repeated scanning, which reflects that HS2 converts to a mixture of LS3 and LS4 on cooling, and LS4 to HS1 and HS2 on rewarming.

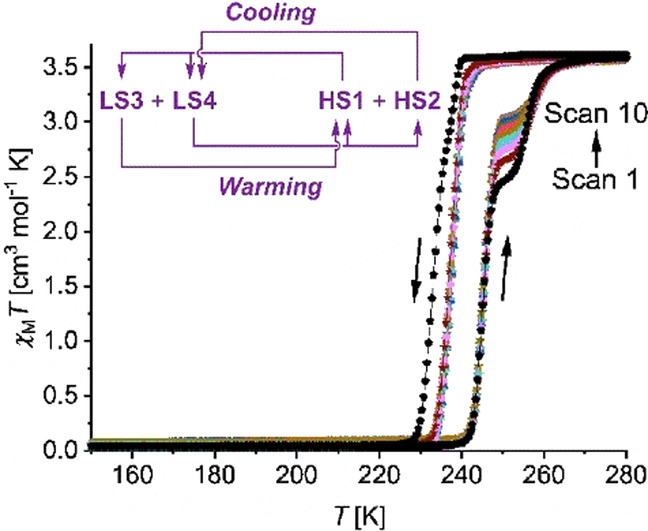

## Introduction

Spin‐crossover (SCO) materials are molecular solids or network structures containing metal ions, which undergo a change in their total electronic spin (*S*) upon heating, cooling, or another physical stimulus.[^1^, ^2^, ^3^, ^4^, ^5^] SCO can occur in metal ions with a 3*d* ^4^–3*d* ^7^ electron count, and is especially common in iron chemistry. There is continued interest in SCO materials as switching components for nanoscience and molecular electronics,[^5^, ^6^, ^7^, ^8^] and for macroscopic applications such as solid state refrigeration,[^9^, ^10^, ^11^, ^12^] mechanical actuation[^13^, ^14^, ^15^] and thermochromic printing.[^16^, ^17^] More fundamentally, spin transitions are also important mechanistic models for phase transitions and other structural or chemical transformations in the crystalline state.[^18^, ^19^] The switching properties of SCO materials reflect the interplay between the steric and electronic properties of the individual switching centers, and of the lattice containing them.[^4^, ^20^] Designing new materials with tailored switching properties from these principles is an ambitious challenge for crystal engineering.[^20^, ^21^, ^22^, ^23^]

Thermal SCO in the solid state can occur gradually or abruptly with temperature, continuously or discontinuously (*ie* in two or more discrete steps), and with or without thermal hysteresis.[^1^, ^2^, ^3^] Crystallographic phase changes may also occur as SCO proceeds, and are often important for mediating cooperative spin‐transitions.[^23^, ^24^, ^25^] Complicated multi‐step spin‐transitions can occur involving more than one phase change,[^26^, ^27^, ^28^, ^29^, ^30^, ^31^, ^32^, ^33^, ^34^, ^35^] and/or crystallographically independent metal centers switching independently of each other.[^36^, ^37^, ^38^] An unusual, related phenomenon is SCO asymmetry where one branch of the transition is discontinuous (ie stepped) while the other is not.[^39^, ^40^, ^41^, ^42^, ^43^, ^44^, ^45^, ^46^, ^47^, ^48^]

**Scheme 1:**
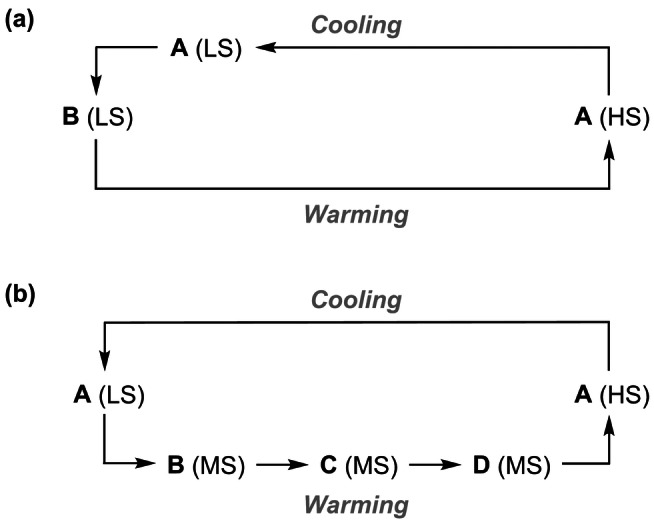
Two forms of interplay between spin states and structural phases (**A**–**D**) leading to asymmetric SCO hysteresis in different materials (HS=high‐spin, LS=low‐spin, MS=a mixed high:low‐spin state population). The shoulder in (a) can occur on the cooling branch (as shown)[^43^, ^44^, ^45^] or the warming branch^[46]^ of the transition in different materials.

Most examples of asymmetric SCO with available structural data are associated with a temperature‐dependent conformational rearrangement, which may or may not involve a crystallographic phase change. That conformational change induces an additional kinetic barrier to one branch of the spin‐transition, leading to asymmetric hysteresis (Scheme 1a).[^43^, ^44^, ^45^, ^46^] However a different mechanism operates in [Fe(bpp)_2_][Ni(mnt)_2_]_2_ ⋅ MeNO_2_ (bpp=2,6‐bis{pyrazol‐1‐yl}pyridine, H_2_mnt=maleonitriledithiol), whose SCO occurs in one step on cooling and in four discrete steps on warming. This reflects a re‐entrant sequence of crystal phase changes in the warming branch of the transition only, which are mediated by reversible dimerization of the [Ni(mnt)_2_]⋅^−^ radical anions in the structure (Scheme 1b).^[47]^

**Scheme 2:**
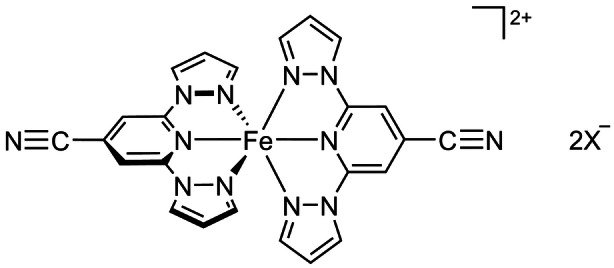
The structure of [FeL_2_]X_2_ (X^−^=BF_4_^−^, ClO_4_^−^, PF_6_^−^ or CF_3_SO_3_^−^).

**Figure 1:**
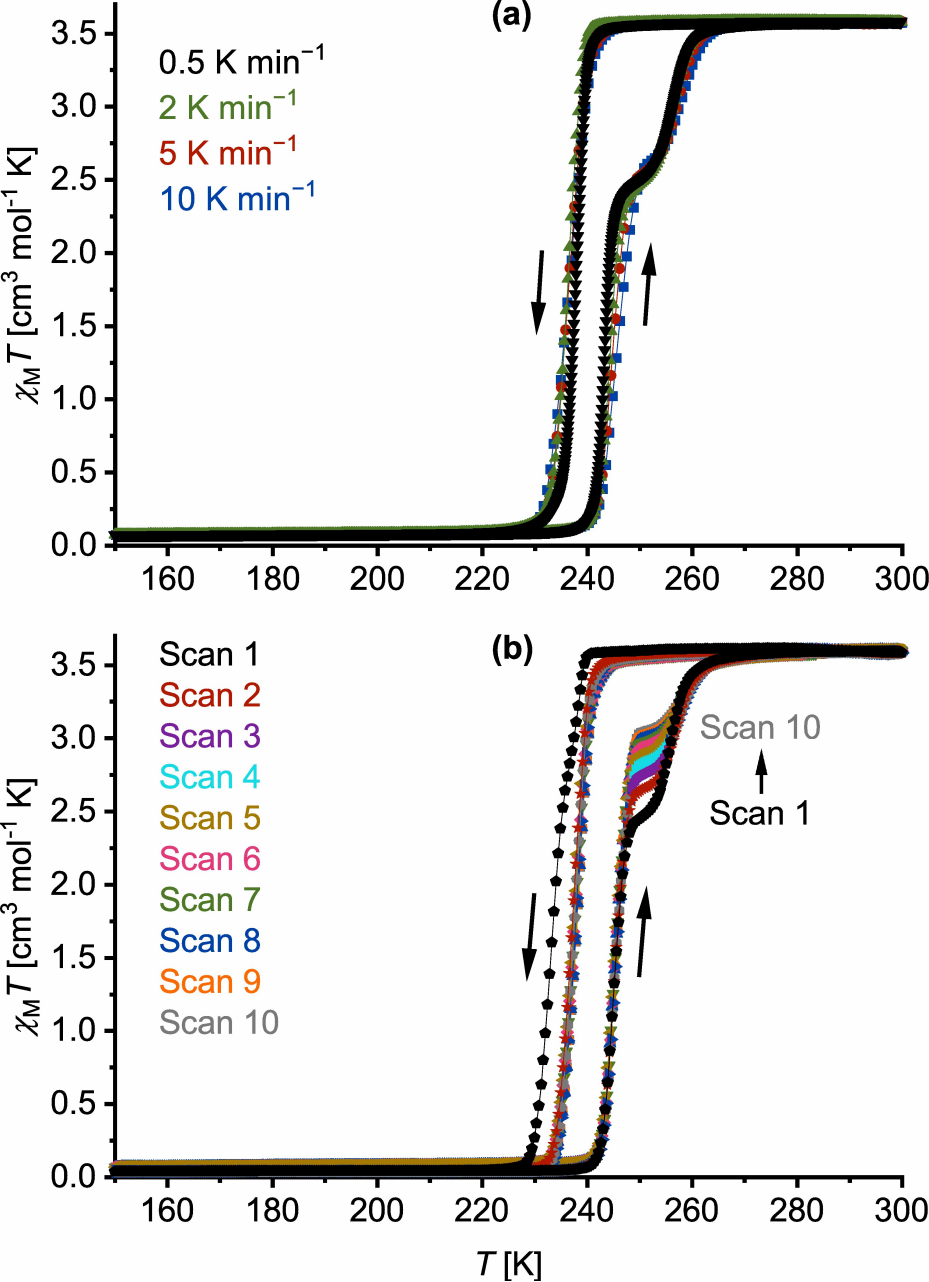
Magnetic susceptibility data for polycrystalline [*Fe*L_*2*_][BF_4_]_2_ measured in cooling and heating modes: (a) four different samples measured at the scan rates shown; (b) the same sample measured with ten 300→150→300 K thermal cycles at 5 K min^−1^. Data from each measurement are connected by spline curves for clarity. These curves are plotted individually in Figures S3 and S5.

Kumar et al. have reported the new ligand 2,6‐di(pyrazol‐1‐yl)isonicotinonitrile (L), and two salts of its iron complex (Scheme 2).^[48]^ Freshly crystallized [FeL_2_][BF_4_]_2_ shows an abrupt, hysteretic spin‐transition near 240 K, with a clear shoulder near the high‐spin side on the warming branch of the hysteresis loop (Figure 1). They showed the shoulder is reduced, or does not appear, in aged or rapidly precipitated samples (Figure 1b). However, no structural data to explain these observations were included in that study.

We had also independently prepared L and its iron complex salts, including [FeL_2_][BF_4_]_2_. We now present structural data which rationalize the asymmetric hysteresis in Figure 1. The underlying structural chemistry is very different from those represented in Scheme 1 and includes rare bifurcated structural phase transitions, where one phase transforms simultaneously into two different phases upon cooling or warming.^[49]^

## Results and Discussion

### Synthesis and Analytical Characterization

Kumar et al. synthesized L by treating 4‐iodo‐2,6‐di(pyrazol‐1‐yl)pyridine with CuCN.^[48]^ We used the alternative approach, of reacting commercially available 2,6‐dichloroisonicotinonitrile with 2 equiv. sodium pyrazolate. This afforded L in one step in up to 55 % yield, without the use of toxic cyanide reagents. Complexation of L by 0.5 equiv. of the appropriate iron(II) salt yielded [FeL_2_]X_2_ (X^−^=BF_4_^−^, ClO_4_^−^, PF_6_^−^ or CF_3_SO_3_^−^; Scheme 2) as orange or red powders after the usual work‐up. The products are sparingly soluble in common solvents, but small quantities of polycrystalline solvent‐free material can be obtained from nitromethane using di‐*iso*propyl ether vapor as antisolvent.^[50]^

Magnetic data from polycrystalline [FeL_2_][BF_4_]_2_ are consistent with the previous report (Figure 1).^[48]^ The material undergoes abrupt SCO with a small thermal hysteresis. The hysteresis loop has a discontinuity on the warming branch at *χ*_M_ *T*=2.4 cm^3^ mol^−1^ K in freshly prepared samples, when the low→high‐spin transition is *ca* 67 % complete. The transition temperatures are *T*_1/2_↓=238 K and *T*_1/2_↑=244, 256 K at a scan rate of 0.5 K min^−1^. Unusually the hysteresis is almost independent of the scan rate (Figure 1a). That implies the asymmetry of the transition does not reflect a significant kinetic barrier to SCO,^[51]^ or any associated structural rearrangement.[^43^, ^44^, ^45^, ^46^, ^47^] Although it is not apparent in the raw data, first derivative plots of the data in Figure 1a also appear to show structure on the cooling branch of the hysteresis loop (Figure S4). Hence, the high→low‐spin transition may also contain two closely spaced components.

Slightly wider hysteresis, with structure on the cooling branch, was observed from one sample which may have contained larger or higher quality crystallites.^[51]^ However this collapsed after one thermal cycle to the narrower hysteresis observed elsewhere in this study (Figure 1b). Multiple thermal scans of that sample at a constant scan rate leads the warming shoulder to lose intensity as the experiment proceeds, from *χ*_M_ *T*=2.5 cm^3^ mol^−1^ K (70 % transition completeness) in scan 1 to 3.1 cm^3^ mol^−1^ K (85 % completeness) in scan 10. These features are reproduced by differential scanning calorimetry (DSC), where the spin‐transition manifests as one cooling peak and two warming peaks at temperatures consistent with the magnetic data. The enthalpy of the lower temperature warming peak increases, and the higher temperature one decreases, as the sample is repeatedly cycled (Figure S6). Similar observations were noted in ref. [48].

### Single‐Crystal X‐Ray Diffraction of [FeL_2_][BF_4_]_2_

Solvent‐free [FeL_2_][BF_4_]_2_ crystallized as stacks of plates, which were challenging to analyze (Figure S7). While the samples were visually homogeneous, investigation of different crystals revealed they contain two polymorphic phases at room temperature. These are: a triclinic phase (HS1; space group *P*1, Z=2), and, a tetragonal phase (HS2; *P*4_3_, Z=4). There are clear similarities between the unit cell dimensions of these phases (Table 1), in that HS1 is related to HS2 by a doubling of the unit cell *c* dimension. Crystallographic refinements of HS1 and HS2 had low precision and high residuals reflecting their twinned, weakly diffracting nature. However their crystallographic symmetry and chemical connectivity are clear, and our structural assignments of HS1 and HS2 are confirmed by the powder diffraction data described below.

**Table 1:** Single‐crystal unit cell parameters for the phases of [FeL_2_][BF_4_]_2_.

	HS1 (300 K)	LS3 (120 K)	HS2 (300 K)	LS4 (150 K)
Space group	*P* $\overset{-}{1}$	*P* $\overset{-}{1}$	*P*4_3_	*P*2_1_/*n*
*Z*	2	4	4	4
*a* [Å]	8.4864(13)	8.5664(6)	8.4905(4)	8.234(6)
*b* [Å]	8.5043(12)	16.7521(8)	8.4905(4)	38.10(2)
*c* [Å]	19.871(3)	19.3801(19)	39.283(3)	8.634(7)
*α* [deg]	81.785(11)	95.278(6)	90	90
*β* [deg]	87.899(10)	90.487(7)	90	94.65(5)
*γ* [deg]	89.859(9)	91.814(5)	90	90
*V* [Å^3^]	1418.4(4)	2767.8(4)	2831.9(3)	2700(3)

The [FeL_2_]^2+^ molecules in HS1 and HS2 are high‐spin, and adopt similar coordination geometries which are significantly distorted from their idealized *D*_2d_ symmetry (Figures S8 and S9). Detailed analysis of their metric parameters is inappropriate, however, given the low precision of the refinements. Both HS1 and HS2 adopt versions of the well‐known “terpyridine embrace” lattice type, based on layers of interdigitated complex cations in the (001) crystal plane (Figure S10).^[52]^ The terpyridine embrace structure type is quite common in [Fe(bpp)_2_]Y_2_ (Y^−^=BF_4_^−^ or ClO_4_^−^) derivatives with small ligand substituents, where it is often associated with abrupt spin‐transitions with narrow thermal hysteresis (Δ*T*_1/2_≤5 K).[^21^, ^53^, ^54^, ^55^]

**Figure 2:**
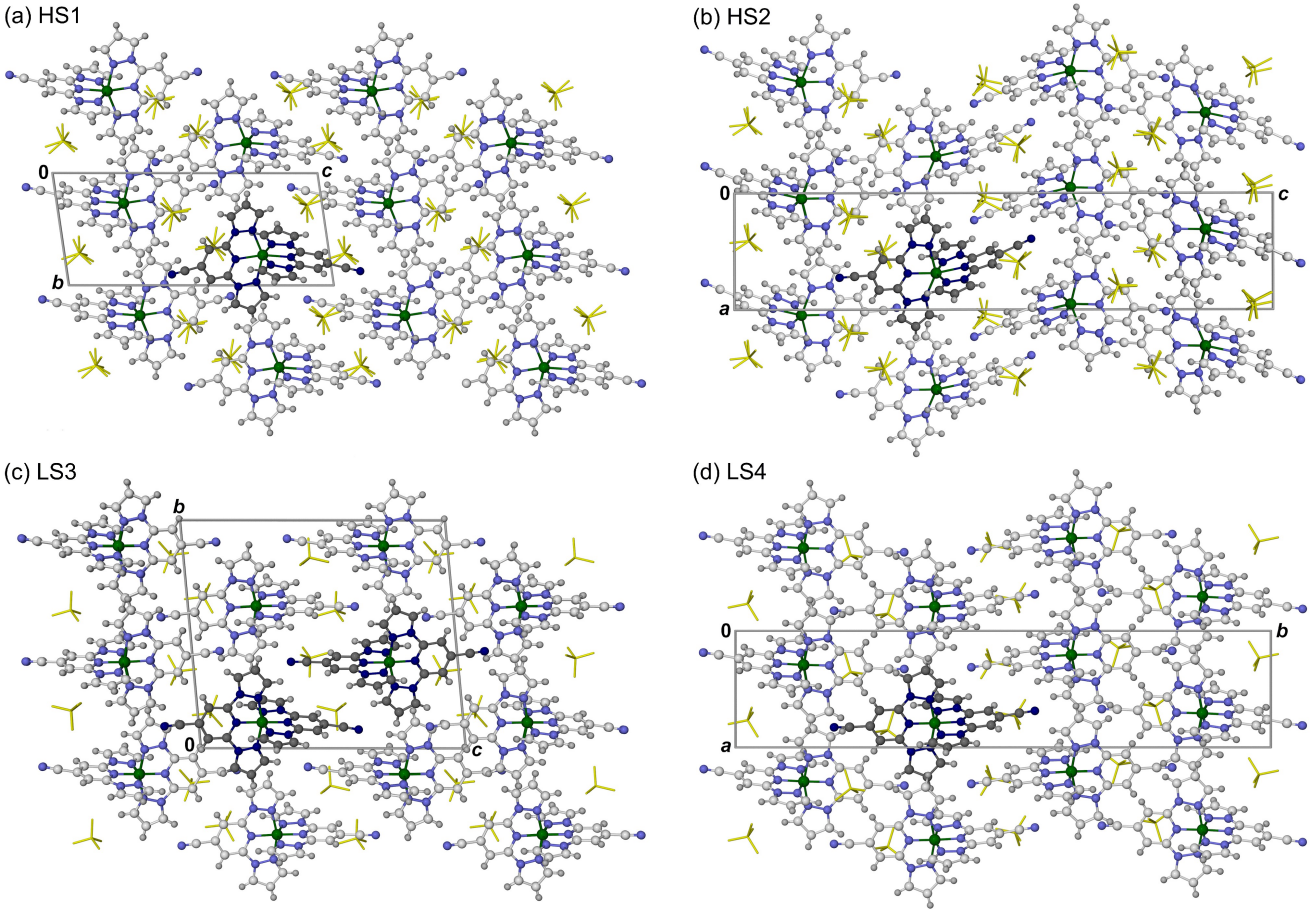
Packing diagrams of the phases of [FeL_2_][BF_4_]_2_ (Table 1): (a) HS1, (b) HS2, (c) LS3, (d) LS4. (a)–(c) are from single‐crystal structure refinements of those phases, while (d) is a model derived from a Rietveld refinement of synchrotron powder diffraction data using rigid body cation and anion moieties.^[57]^ The unique cation environment(s) in each structure are shown in dark coloration, while the other molecules are de‐emphasized for clarity. All atoms have arbitrary radii. Color code: C, white or dark grey; H, pale grey; Fe, green; N, pale or dark blue; BF_4_^−^, yellow.

There are two main points of difference between HS1 and HS2 (Figure 2). First, the cation layers propagate along *c* by inversion symmetry in HS1, and via the 4_3_ screw axis in HS2. Thus, the cation layers adopt a two‐fold repeat pattern in HS1 and a four‐fold repeat in HS2, which accounts for the doubled *c* axis in HS2 (Table 1). Second, the least‐squares plane of each cation layer in HS1 is canted by *ca* 8° from perpendicular with respect to the *c* axis, whereas in HS2 they are perpendicular to it by symmetry.

**Figure 3:**
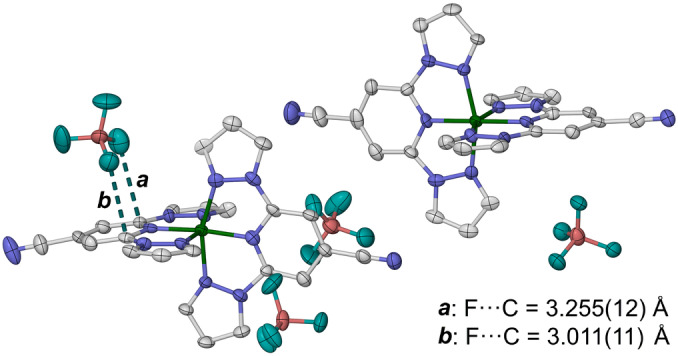
The asymmetric unit of the LS3 phase of [FeL_2_][BF_4_]_2_. Intermolecular anion⋅⋅⋅*π* contacts that may contribute to the bent ligand conformation of cation A, on the left, are also shown. Displacement ellipsoids are at the 50 % probability level, and H atoms are omitted. Color code: C, white; B, pink; F, cyan; Fe, green; N, blue.

The unit cells of HS1 and HS2 crystals were monitored through a 300→150→300 K temperature cycle. Cooling the HS1 crystal from 250 to 200 K transformed it to a new triclinic LS3 phase *(P*1, *Z*=4), which differs from HS1 by a doubling of the *b* axis with a switch in the handedness of the triclinic cell.^[56]^ The crystal then reformed HS1 on rewarming between the same temperatures (Table S5). A precise refinement of LS3 was achieved with a twinned X‐ray dataset from a different crystal at 120 K, which has two formula units of the compound in the asymmetric unit. This phase contains two unique cations, labelled ‘A’ and ‘B’, which are both low‐spin from their metric parameters but differ in their ligand conformations. One ligand in each cation has a bowl shaped conformation, with its 4‐cyanopyridyl group being displaced out of its meridional plane. This conformational distortion is larger in molecule A than in molecule B, which might reflect the influence of an anion⋅⋅⋅*π* contact between that pyridyl group and a BF_4_^−^ ion (Figure 3). The corresponding ligand in molecule B is also overlaid by a BF_4_^−^ ion, but with a longer C⋅⋅⋅F distance of 3.4 Å.

The ligand conformation in molecule A also influences its coordination geometry, through the *trans*‐N{pyridyl}‐Fe−N{pyridyl} angle (*φ*).[^53^, ^58^] This is *φ*=173.6(3)° for molecule A, which is unusually low for a low‐spin [Fe(bpp)_2_]^2+^ derivative,^[58]^ while molecule B exhibits *φ*=178.6(3)° which is closer to its ideal value of 180°. Since changes to *φ* during SCO are associated with hysteretic transitions in some [Fe(bpp)_2_]^2+^ derivatives,[^59^, ^60^, ^61^, ^62^, ^63^] this conformational deformation of molecule A may be relevant to the cooperative SCO properties of [FeL_2_][BF_4_]_2_ (Figure 1).

The [FeL_2_]^2+^ cations in LS3 associate by intermolecular face‐to‐face *π*⋅⋅⋅*π* and edge‐to‐face C−H⋅⋅⋅*π* contacts between their pyrazolyl rings. This four‐fold interdigitation of their pyrazolyl groups again affords “terpyridine embrace” cation layers in the (001) plane.^[52]^ Each layer contains an equal number of A and B cation sites, which are segregated into chains by translation along the unit cell *a* axis (Figure S12). These chains alternate by translation along *b* to form the cation layers, which then propagate into three dimensions via the crystallographic inversion symmetry (Figure 2).

The HS2 crystal also changed between 250 and 200 K, to a lower symmetry LS4 phase (Table 1). The crystal system and space group of LS4 were not uniquely defined by these data, but it was identified as a monoclinic phase by the synchrotron powder diffraction study described below (*P*2_1_/*n*, Z=4; Figure 2).^[57]^ The *b* and *c* axes are exchanged during the HS2→LS4 transition, while the 4_3_ screw axis relating the cation layers in HS2 is replaced by an alternate 2_1_ screw axis/*n* glide sequence in LS4. Unexpectedly, rewarming the LS4 crystal transformed it to triclinic HS1 at a slightly higher temperature, between 250 and 275 K (Table S6).

### X‐Ray Powder Diffraction of [FeL_2_][BF_4_]_2_

**Figure 4:**
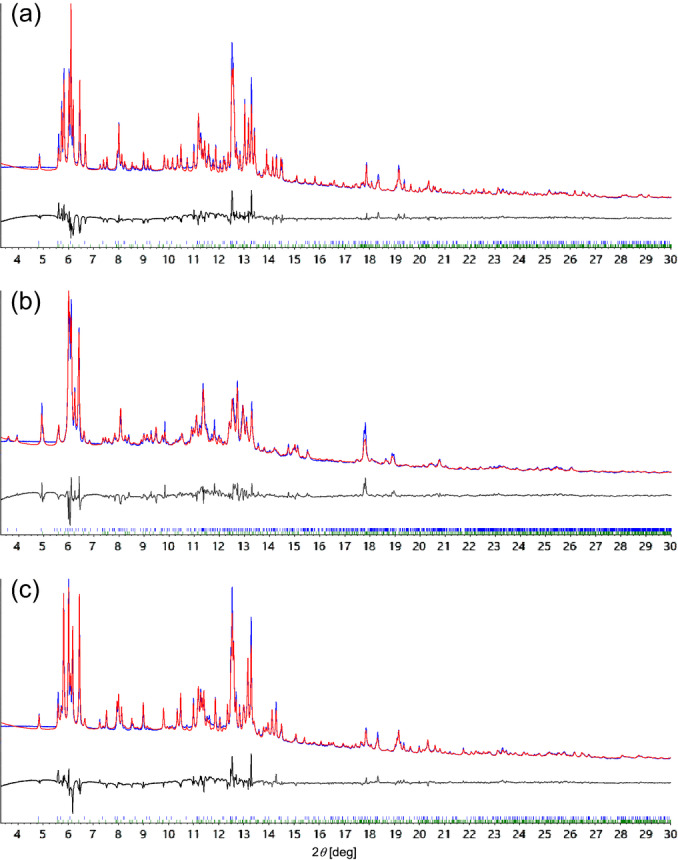
Rietveld fits of synchrotron powder diffraction data from [FeL_2_][BF_4_]_2_: (a) as‐prepared at 300 K, a mixture of HS1+HS2; (b) at 150 K, fitted as a mixture of LS3+LS4; (c) rewarmed to 300 K, as a different ratio of HS1+HS2. Measured data are plotted in dark blue, fits are in red and difference plots are in gray. The fitted parameters are listed in Table S7.

The X‐ray powder diffraction pattern of [FeL_2_][BF_4_]_2_ is complex, and contains more than one solid phase. Data collected with synchrotron radiation, from two different samples, confirmed they were a mixture of the HS1 : HS2 phases at 300 K. These converted to a LS3 : LS4 mixture at 150 K, then reverted back to HS1 : HS2 upon rewarming (Figure 4). Rigid body Rietveld refinements^[64]^ from these data determined the crystallographic symmetry of LS4. They also implied that the HS1 fraction of the high‐spin samples changed during the experiment, from *ca* 60 % in the as‐isolated materials to 79–85 % following the thermal cycle (Table S7).

**Figure 5:**
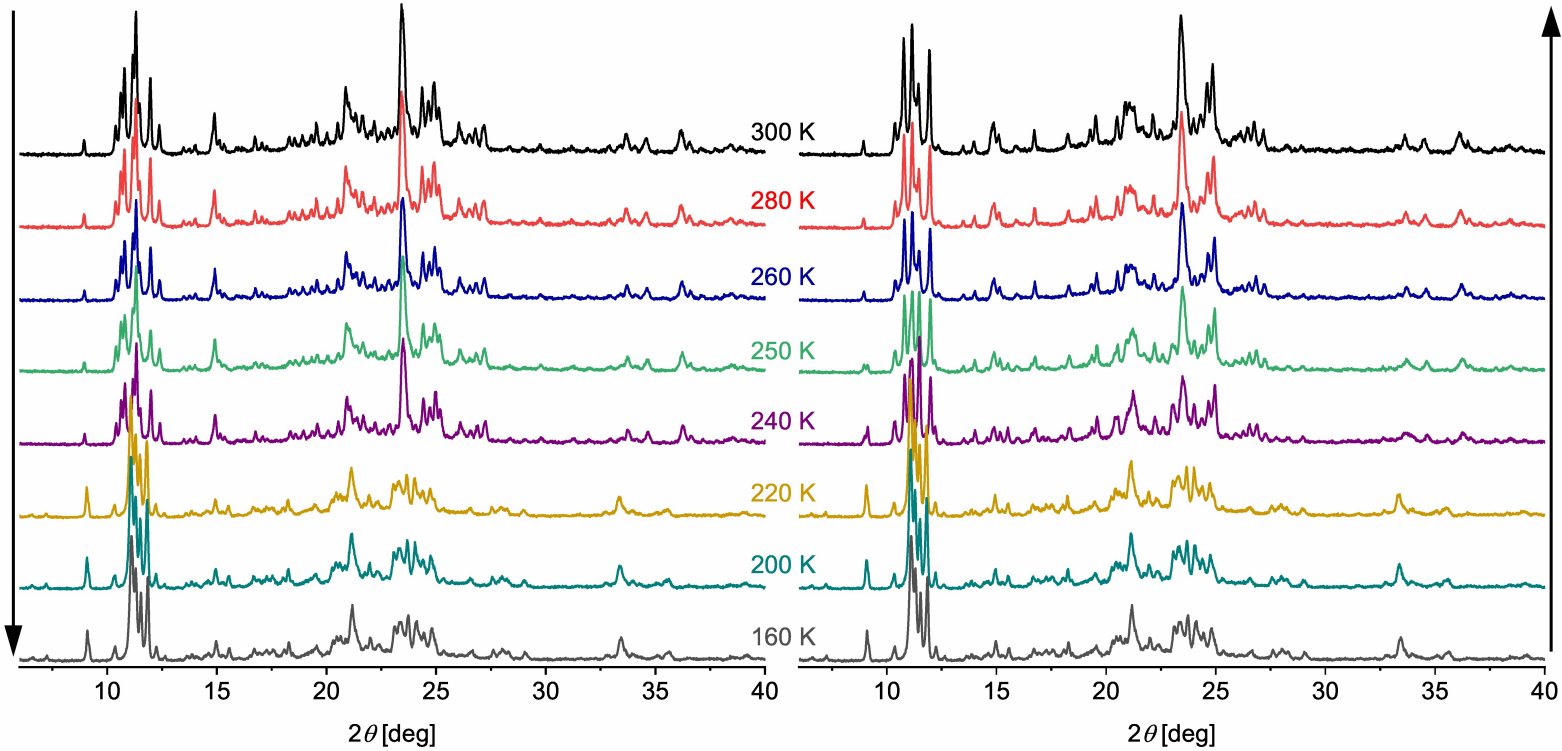
Selected variable temperature X‐ray powder diffraction data for [FeL_2_][BF_4_]_2_, measured in cooling (left) and warming (right) modes. Additional data are plotted in Figure S15. The initial and final 300 K powder patterns contain different ratios of the HS1 and HS2 phases; see the text for more details.

A more detailed investigation of [FeL_2_][BF_4_]_2_ was undertaken with a laboratory powder diffractometer using Cu−*K*_*α*_ radiation (Figure 5). The pattern measured at 300 K was unchanged on cooling to 240 K, before transforming at 220 K to a new pattern which is retained on further cooling to 160 K. This change corresponds to the complete high→low‐spin transition in the magnetic data (Figure 1). The pattern of the low‐spin phases is retained on rewarming until 220 K, then evolves slowly between 240–260 K towards its original form (Figures 5 and S15). That is also consistent with the magnetic data, showing a stepwise low→high‐spin transition within that temperature window (Figure 1). No more changes were observed on warming from 260 to 300 K. Pawley refinements^[65]^ of these data confirmed the sample is a mixture of HS1+HS2 between 300–240 K, then contains LS3+LS4 from 220 to 160 K (Figure S16). On rewarming, the data were fitted using the following components: 160–220 K, LS3+LS4; 235–245 K, HS1+LS3+LS4; 250 K, HS1+HS2+LS4; and 255–300 K, HS1+HS2 (Table S8). This supports the suggestion from the single‐crystal unit cell data, that the LS4 phase undergoes its low→high‐spin transition at a slightly higher temperature than LS3. It also rationalizes the shoulder on the warming branch of the hysteresis loop in Figure 1.

**Figure 6:**
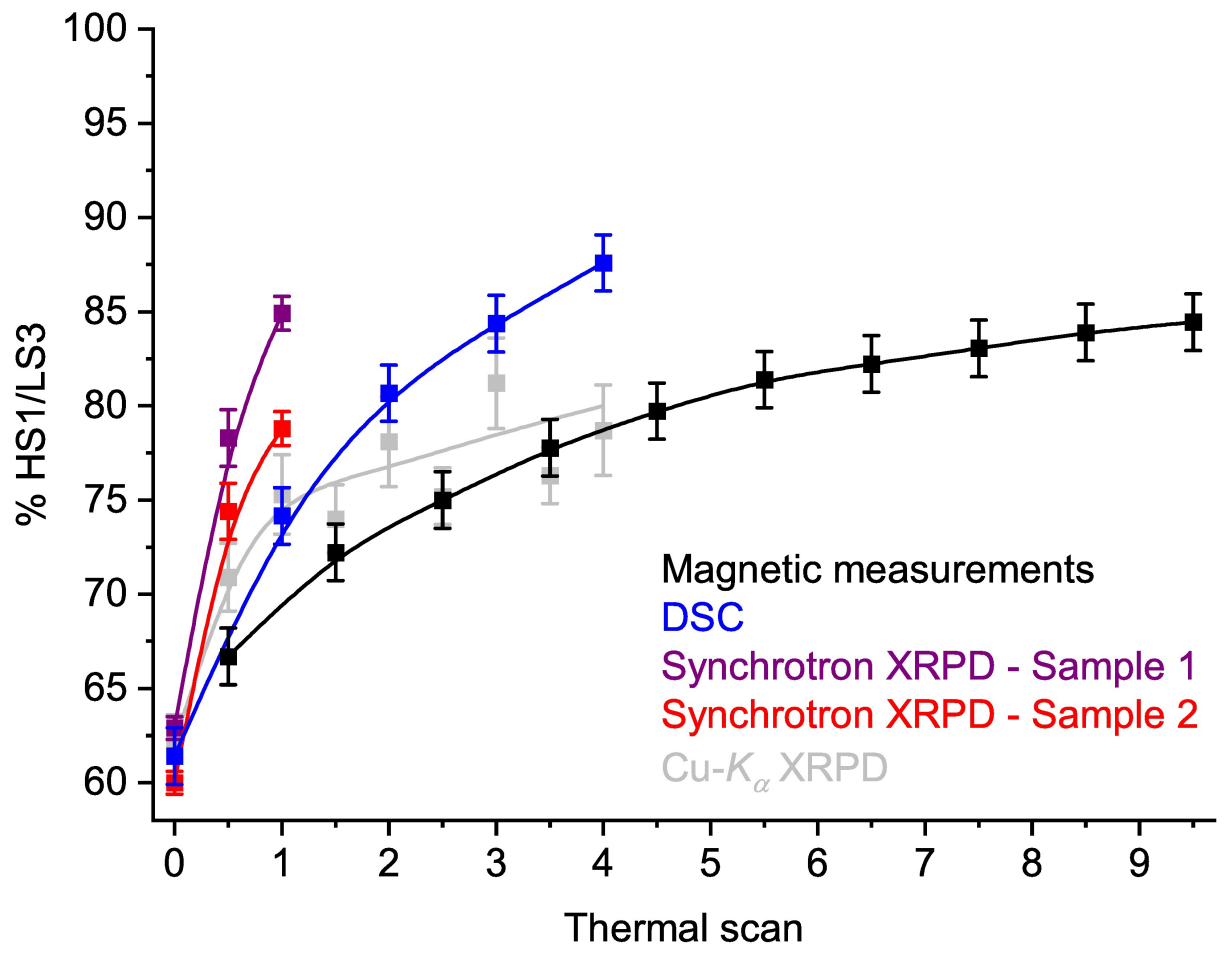
The phase composition of [FeL_2_][BF_4_]_2_ upon multiple thermal scanning, measured by different techniques (XRPD=X‐ray powder diffraction; Figure S5, and Tables S2, S7 and S10). Each set of data is connected by a regression curve for clarity. The compositions are expressed as the percentage of the sample adopting the HS1 or LS3 phase. The errors bars on the powder diffraction data correspond to 3*σ*, while the other data are plotted with estimated errors.

The HS1+HS2 powder pattern at 300 K again showed differences after thermal cycling (Figure 5). This was addressed by measuring a fresh sample on a 300→250→180→235→250→275→300 K temperature programme, which was repeated four times. The general pattern of phase changes in the sample was unchanged during this process (Table S9). However, Rietveld refinements^[64]^ of the 300 and 180 K data from each cycle confirmed the sample became enriched with HS1 or LS3 as the experiment proceeded (Table S10). The fresh sample yielded a HS1 : HS2 ratio of 0.621(5) : 0.379(5) at 300 K. After one thermal cycle that ratio refined to 0.753(7) : 0.247(7), and after a second cycle it was 0.781(8) : 0.219(8). Repeating the same cycle for a third and fourth time, then annealing the sample at 398 K for 24 hrs, led to no further changes in the sample composition within experimental error (Figure 6). In scans 2–4, the fitted fraction of LS3 at 180 K is consistently 3–6 % lower than for HS1. However, since these differences are often within experimental error, their significance is unclear.

**Scheme 3:**
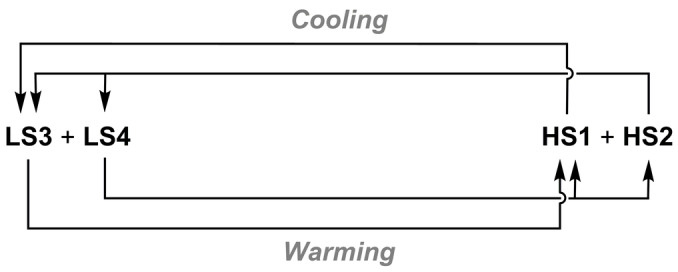
The interplay between crystal phases giving rise to the asymmetric SCO hysteresis in [FeL_2_][BF_4_]_2_ (*cf* Scheme 1).

All these data show that repeated cycling about the spin‐transition leads to slow enrichment of HS1 and LS3, at the expense of HS2 and LS4 (Figure 6). That explains why the shoulder on the warming hysteresis branch in the magnetic data becomes less intense upon multiple scanning, as the fraction of HS2/LS4 in the sample is depleted (Figure 1b).^[48]^ Some support for this was obtained from the single crystal data, where cooling and heating crystals of HS2 led to a HS2→LS4→HS1 sequence of phase changes (Table S6). However, cooling HS2 and warming LS4 in powder samples must lead to bifurcated HS2→(LS3+LS4) and LS4→(HS1+HS2) transitions since some HS2 and LS4 is retained after thermal cycling (Scheme 3).

While this phase enrichment behavior was observed in all our measurements, the rate of enrichment of HS1/LS3 differs between the experiments. In the magnetic data and the Cu−*K*_*α*_ powder diffraction, the composition of the sample eventually plateaus at *ca* 80 % HS1. However, a steady state composition was not achieved in the other data in Figure 6, which imply full conversion to HS1/LS3 might eventually be reached after additional thermal scans. Be that as it may, the phase behavior of [FeL_2_][BF_4_]_2_ depends on the sample studied and on the technique used to measure it.

### Other Salts and Reactions of the Iron Complex

Single crystals of [FeL_2_][ClO_4_]_2_ and [FeL_2_][PF_6_]_2_ are isomorphous with each other (monoclinic, *P*2_1_/*c*, *Z*=4), but not with the BF_4_^−^ salt. Crystals of [FeL_2_][CF_3_SO_3_]_2_ (monoclinic, *P*2_1_/*n*, *Z*=4) adopt a third type of crystal lattice. All three salts are crystallographically low‐spin at 120–125 K, with one unique molecule per asymmetric unit and no noteworthy structural features (Table S11). While their crystal structures are formed from layers of complex cations, the molecules in those layers are more loosely associated and do not interdigitate in a terpyridine embrace motif (Figures S20–S24). Polycrystalline and rapidly precipitated powder samples of [FeL_2_][PF_6_]_2_ and [FeL_2_][CF_3_SO_3_]_2_ are phase‐pure, and isomorphous with their single‐crystal phases. Both salts are low‐spin at room temperature, but exhibit the onset of gradual SCO on heating above 320 K (Figures S26–S28).

The phase behavior of [FeL_2_][ClO_4_]_2_ is more complicated, however. Samples of its pure single‐crystal phase are low‐spin below 350 K as predicted crystallographically, but are difficult to obtain. Most samples of that material were contaminated by a second polycrystalline phase, which exhibits a hysteretic spin‐transition at *T*_1/2_=240 K (Δ*T*_1/2_=30 K; Figure S29). The SCO‐active phase of [FeL_2_][ClO_4_]_2_ was isolated as a pure material in ref. [48], but was not structurally characterized. In our hands, the SCO‐active material always co‐crystallized with the low‐spin single‐crystal phase. However, comparison of the powder diffraction data in ref. [48] with simulations based on the results from this study, confirms the SCO phase of [FeL_2_][ClO_4_]_2_ is isomorphous with HS2 (Figure S30). That being the case, it is notable that its SCO is unchanged after multiple thermal scans.^[48]^ SCO in the HS2 phase of [FeL_2_][ClO_4_]_2_ does not show the same phase bifurcation as in the BF_4_^−^ salt.

In an attempt to link [FeL_2_]^2+^ centers into chains through their pendant nitrile groups, [FeL_2_]X_2_ (X^−^=BF_4_^−^, ClO_4_^−^, CF_3_SO_3_^−^) were treated with the corresponding silver(I) salts in different mole ratios and solvents. This always led to rapid precipitation of insoluble white powders, which proved to be the silver complexes [AgL]X. A crystal structure of [Ag(*μ*‐L)]BF_4_ ⋅ 0.5MeNO_2_ (triclinic, *P*1, *Z*=4) showed a linear coordination polymer containing two unique silver ions with typically distorted square planar geometries,^[66]^ linked by *κ* ^1^ : *κ* ^3^‐*μ*‐L ligands (Figure S32).

## Conclusion

The structural chemistry underlying the asymmetric spin‐transition shown by [FeL_2_][BF_4_]_2_ reported by Kumar et al. (Figure 1)^[48]^ differs from other examples of SCO asymmetry.[^39^, ^40^, ^41^, ^42^, ^43^, ^44^, ^45^, ^46^, ^47^] The freshly crystallized material contains two polymorphic phases, triclinic HS1 and tetragonal HS2, which have closely related unit cell dimensions and are variations of same layered crystal packing motif (Figure 2). HS1 and HS2 undergo abrupt high→low‐spin transitions at coincidentally similar temperatures, *T*_1/2_=238 ±1 K. HS1 forms a new triclinic low‐spin phase LS3 with a doubled unit cell *b* axis. The low‐spin counterpart of HS2, LS4, has monoclinic symmetry with similar unit cell dimensions to HS2.

The more distorted metal coordination geometries in HS1, HS2 and LS3 are not replicated in the other crystalline, low‐spin salts of the complex. Since such distortions are known to stabilize the high‐spin state in compounds related to [FeL_2_]^2+^, that should explain why only the BF_4_^−^ salt of [FeL_2_]^2+^ is SCO‐active in this study.[^21^, ^67^]

In warming mode, LS3 and LS4 exhibit their low→high‐spin transitions at different temperatures, *T*_1/2_↑=244 and 256 K respectively. While the HS1 and LS3 phases interconvert cleanly, the powder diffraction data imply the HS2 fraction of the sample transforms to a mixture of LS3 and LS4, while rewarming LS4 produces a mixture of HS1 and HS2 (Scheme 3). Some support for that comes from single crystal studies of HS2, which showed a HS2→LS4→HS1 sequence of phase changes on thermal cycling. That explains why the HS1 : HS2 ratio in different measurements consistently increases from 0.62 : 0.38±0.02 in freshly crystallized material, to 0.80 : 0.20±0.02 after one, two or three thermal cycles (Figure 6). The enrichment of HS1 and depletion of HS2 are also reflected in magnetic measurements and DSC data upon repeated thermal cycling (Figures 1b and S6).^[48]^ Importantly these changes are not caused by slow loss of lattice solvent, which progressively changes the magnetic data upon multiple scanning in some other SCO compounds.[^68^, ^69^, ^70^, ^71^, ^72^]

Behavior resembling the LS4→(HS1+HS2) transition has not been seen before in SCO materials. Indeed, bifurcated crystallographic phase transitions in general are extremely rare, and only one example has been crystallographically characterized.^[49]^ In that compound, single crystals of the ambient phase transform simultaneously on heating into two conformational polymorphs,^[73]^ which are related by hindered rotation of an anthryl substituent. Since the molecular conformations and crystal packing in HS1 and HS2 are similar, the bifurcated transformation of LS4 should have a different origin.

Notably however, the rate of enhancement of HS1/LS3 differs significantly between the measurements (Figure 6). Firstly, the two synchrotron powder diffraction samples gave different results, implying their phase transitions could be sample dependent. That is consistent with the sensitivity of SCO in [FeL_2_][BF_4_]_2_ to the sample preparation method used, which was explored in ref. [48]. Particle size and crystallinity are known to influence SCO transitions,[^74^, ^75^, ^76^, ^77^, ^78^, ^79^, ^80^] and the relative stability and interconversion rate of polymorphic phases in other types of compound.[^81^, ^82^, ^83^, ^84^, ^85^, ^86^] Secondly, in some measurements the sample reached a steady state composition of *ca* 80 % HS1/LS3, whereas in other measurements that plateau value was surpassed. Interestingly the fastest measurement techniques in Figure 6, synchrotron powder diffraction and DSC, give the largest rate and magnitude of HS1/LS3 enhancements. However the significance of that is unclear, since SCO in fresh samples of [FeL_2_][BF_4_]_2_ is almost independent of scan rate in the SQUID magnetometer (Figure 1a).

## Supporting Information

Synthetic methods, analytical data and measurement protocols; crystallographic Figures and Tables; additional Figures of magnetic, calorimetry and powder diffraction data, and other spectra; and, tabulated unit cell data from the Pawley and Rietveld refinement fits. The authors have cited additional references within the Supporting Information.[^87^, ^88^, ^89^, ^90^, ^91^, ^92^, ^93^, ^94^, ^95^, ^96^, ^97^, ^98^, ^99^, ^100^, ^101^, ^102^, ^103^, ^104^]

## Author Contributions

The synthesis and analytical characterization of the compounds was performed by AA and AH. HBV and RK performed the magnetic measurements, using SQUID magnetometer time provided by OC. TMR, CMP and RK collected and refined the single‐crystal diffraction data. TMR designed and performed the variable temperature powder diffraction study, while LB contributed with TMR to interpretation of the diffraction data. ANK provided the SEM images. MAH conceived and supervised the study, and prepared the publication. All authors have approved the final version of the manuscript.

## Conflict of Interests

The authors declare no conflict of interest.

1

## Supporting information

As a service to our authors and readers, this journal provides supporting information supplied by the authors. Such materials are peer reviewed and may be re‐organized for online delivery, but are not copy‐edited or typeset. Technical support issues arising from supporting information (other than missing files) should be addressed to the authors.

Supporting Information

## Data Availability

The data that support the findings of this study are openly available in the University of Leeds Library at https://doi.org/10.5518/1564.
