# Supplementary material for: Structural Bifurcation in the High→Low‐Spin and Low→High‐Spin Phase Transitions Explains the Asymmetric Spin‐Crossover in [FeL2][BF4]2 (L=2,6‐Di{pyrazol‐1‐yl}isonicotinonitrile)
Source: Angew Chem Int Ed Engl. 2024 Dec 17;64(5):e202416924. doi: 10.1002/anie.202416924 (PMC11773316; doi:10.1002/anie.202416924)
Supplement: Supplementary file 1 — Supporting Information [file ANIE-64-e202416924-s001.pdf]

Angewandte Chemie
Eine Zeitschrift der Gesellschaft Deutscher Chemiker

Supporting Information

Structural Bifurcation in the High→Low-Spin and Low→High-Spin Phase Transitions Explains the Asymmetric Spin-Crossover in $[FeL_2][BF_4]_2$ (L = 2,6-Di{pyrazol-1-yl}isonicotinonitrile)

A. Ahmed, A. Hall, H. B. Vasili, R. Kulmaczewski, A. N. Kulak, O. Cespedes, C. M. Pask, L. Brammer, T. M. Roseveare, M. A. Halcrow**

		Page
**Experimental – Synthesis and characterization data.**		S3
**Experimental – Crystallographic details and refinement procedures.**		S4
**Experimental – Other measurements**		S5
**Table S1**	Experimental data for the single crystal structure determinations.	S6
**Figure S1**	^{1}H and ^{13}C NMR spectra of L.	S7
**Figure S2**	The asymmetric unit and packing diagram for the crystal structures of L.	S8
**Angular distortion parameters used to describe the molecular geometries of $[FeL_2]^{2+}$ salts.**		S9
**Scheme S1**	The angular distortion parameters θ and ϕ.	S9
**Figure S3**	Magnetic susceptibility data for polycrystalline $[FeL_2][BF_4]_2$ at different scan rates.	S10
**Figure S4**	First derivative susceptibility plots for $[FeL_2][BF_4]_2$, from the data in Figure S3.	S11
**Figure S5**	Magnetic susceptibility data for polycrystalline $[FeL_2][BF_4]_2$ upon multiple scanning at the same scan rate.	S12
**Figure S6**	Differential scanning calorimetry (DSC) data for polycrystalline $[FeL_2][BF_4]_2$.	S14
**Table S2**	SCO parameters from the DSC data.	S14
**Figure S7**	Scanning electron microscope images of polycrystalline $[FeL_2][BF_4]_2$.	S15
**Figure S8**	The asymmetric unit of the HS1 phase of $[FeL_2][BF_4]_2$ at 300 K.	S16
**Figure S9**	The asymmetric unit of the HS2 phase of $[FeL_2][BF_4]_2$ at 300 K.	S17
**Figure S10**	A terpyridine embrace layer in the HS1 and HS2 phases of $[FeL_2][BF_4]_2$.	S18
**Figure S11**	The asymmetric unit of $[FeL_2][BF_4]_2$ phase LS3 at 120 K.	S19
**Table S3**	Selected bond lengths and angles for the LS3 phase $[FeL_2][BF_4]_2$.	S20
**Figure S12**	A terpyridine embrace cation layer in the LS3 phase of $[FeL_2][BF_4]_2$, showing the arrangement of the 'A' and 'B' cation sites.	S20
**Figure S13**	The intermolecular $\pi\cdots\pi$ and C–H$\cdots\pi$ contacts in the LS3 phase.	S21
**Table S4**	The intermolecular $\pi\cdots\pi$ and C–H$\cdots\pi$ contacts in the LS3 phase.	S21
**Table S5**	Variable temperature unit cell data from a single crystal of HS1.	S22
**Table S6**	Variable temperature unit cell data from a single crystal of HS2.	S22
**Figure S14**	Rietveld refinement fits of $[FeL_2][BF_4]_2$ in its high-spin and low-spin states, from synchrotron powder diffraction data.	S23
**Table S7**	Rietveld refinements of the synchrotron powder diffraction data from $[FeL_2][BF_4]_2$ in its high-spin and low-spin states.	S25
**Figure S15**	Variable temperature powder diffraction data from $[FeL_2][BF_4]_2$ measured with Cu-K_α radiation in warming mode.	S26
**Figure S16**	Representative Pawley refinement fits of variable temperature powder diffraction data.	S27
**Figure S17**	Rietveld refinement fits of $[FeL_2][BF_4]_2$ at 300 and 180 K following successive thermal cycles, and after thermal annealing.	S30
**Table S8**	Pawley phase fits of variable temperature powder diffraction data from $[FeL_2][BF_4]_2$ – full temperature scan.	S34
**Table S9**	Pawley phase fits of variable temperature powder diffraction data from $[FeL_2][BF_4]_2$ – repeated thermal scanning.	S35
**Table S10**	Rietveld refinements of the Cu-K_α powder diffraction data from $[FeL_2][BF_4]_2$ at 300 K and 180 K, during the repeated thermal scans.	S37
**Figure S18**	The asymmetric units of isomorphous $[FeL_2][ClO_4]_2$ and $[FeL_2][PF_6]_2$.	S39

		Page
**Figure S19**	The asymmetric unit of $[FeL_2][CF_3SO_3]_2$.	S40
**Table S11**	Selected bond lengths and angles for $[FeL_2][ClO_4]_2$, $[FeL_2][PF_6]_2$ and $[FeL_2][CF_3SO_3]_2$.	S41
**Figure S20**	Packing diagram of $[FeL_2][PF_6]_2$.	S42
**Figure S21**	The nearest neighbor molecules in the cation layers of $[FeL_2][PF_6]_2$.	S43
**Figure S22**	Intermolecular $\pi\cdots\pi$ (red) and C–H$\cdots\pi$ (cyan) contacts in $[FeL_2][PF_6]_2$ at 120 K.	S44
**Table S12**	The intermolecular $\pi\cdots\pi$ and C–H$\cdots\pi$ contacts in $[FeL_2][ClO_4]_2$ and $[FeL_2][PF_6]_2$.	S44
**Figure S23**	Packing diagram of $[FeL_2][CF_3SO_3]_2$ at 120 K.	S45
**Figure S24**	The nearest neighbor molecules in the cation layers of $[FeL_2][CF_3SO_3]_2$ at 120 K.	S46
**Figure S25**	IR spectra from L, and the polycrystalline salts of $[FeL_2]^{2+}$.	S47
**Figure S26**	X-ray powder diffraction data for crystalline samples of $[FeL_2][ClO_4]_2$, $[FeL_2][PF_6]_2$ and $[FeL_2][CF_3SO_3]_2$.	S48
**Figure S27**	X-ray powder diffraction data for rapidly precipitated powder samples of $[FeL_2][ClO_4]_2$, $[FeL_2][PF_6]_2$ and $[FeL_2][CF_3SO_3]_2$.	S49
**Figure S28**	Magnetic susceptibility data for $[FeL_2][PF_6]_2$ and $[FeL_2][CF_3SO_3]_2$.	S50
**Figure S29**	Magnetic susceptibility data for crystalline and powder samples of $[FeL_2][ClO_4]_2$.	S50
**Figure S30**	Observed and simulated X-ray powder diffraction data for the phase-pure SCO-active form of $[FeL_2][ClO_4]_2$	S51
**Figure S31**	^{1}H NMR spectrum of $[FeL_2][PF_6]_2$.	S52
**Figure S32**	The asymmetric unit of $[Ag(\mu\text{-}L)]BF_4\cdot 0.5MeNO_2$.	S53
**Table S13**	Selected bond lengths and angles in $[Ag(\mu\text{-}L)]BF_4\cdot 0.5MeNO_2$.	S53
**Figure S33**	Packing diagram of $[Ag(\mu\text{-}L)]BF_4\cdot 0.5MeNO_2$.	S54
**Figure S34**	X-ray powder diffraction data for $[Ag(\mu\text{-}L)]BF_4$.	S55
**Figure S35**	IR spectra of L and $[Ag(\mu\text{-}L)]BF_4$.	S55
**References**		S56

Experimental

Synthesis of 2,6-di(pyrazol-1-yl)isonicotinonitrile (L). Ligand L was prepared by a different procedure from the literature protocol.[1] *N*,*N*-Dimethylformamide (dmf; 70 cm^3) was added to a round bottom flask containing sodium hydride (60 % dispersion in mineral oil; 2.5 g, 62.5 mmol), forming a cloudy suspension. Solid 1*H*-pyrazole (3.9 g, 57.4 mmol) was added and the solution was stirred for 30 mins under nitrogen, until H_2 evolution had ceased and most of the solid had dissolved. This mixture was placed in a dropping funnel, and added dropwise to a stirred solution of 2,6-dichloro-isonicotinonitrile (4.0 g, 23.2 mmol) in dmf (50 cm^3) maintained at 110°C. After the addition was complete (*ca* 4 hrs) the mixture was maintained at 110°C for 48 hrs, affording an orange-brown solution and a white suspension. The reaction mixture was cooled to room temperature and quenched with excess water. The pink precipitate was collected by filtration, washed with water (2x 100 cm^3) and dried *in vacuo*. Recrystallization from a 1:1 dichloromethane:hexane mixture yielded L in NMR purity as a pale orange solid. Yield: 3.0 g, 55 %. Mp 156-158 °C. HR-ESI MS *m*/*z* 237.0879 (calcd. for $[HL]^+$ 237.0889); ^1H NMR (300 MHz, $CDCl_3$) $\delta = 6.45$ (dd, 1.5 and 2.8 Hz, 2H, Pz H^4), 7.71 (d, 1.5 Hz, 2H, Pz H^5), 7.95 (s, 2H, Py $H^{3/5}$), 8.43 (d, 2.8 Hz, 2H, Pz H^3); ^{13}C NMR (400 MHz, $CDCl_3$) δ = 109.1 (2C, Py $C^{3/5}$), 111.1 (2C, Pz C^4), 115.9 (1C, *C*≡N), 125.1 (1C, Py C^4), 127.2 (2C, Pz C^5), 143.5 (2C, Pz C^3), 150.8 (2C, Py $C^{2/6}$); IR 2233 cm^{-1} (ν{C≡N}).

The crystallization filtrate contains a more soluble red byproduct, which can form in significant quantities unless care is taken. Avoiding a large excess of the pyrazolate reagent, and dropwise addition of the pyrazolate to the 2,6-dichloro-isonicotinonitrile, improve the yield of the desired product L.

Synthesis of the iron complex salts. The same basic method, described below for $[FeL_2][BF_4]_2$, was used for all the iron complexes in this work. A solution of L (0.25 g, 1.1 mmol) and $Fe[BF_4]_2 \cdot 6H_2O$ (0.18 g, 0.53 mmol) in nitromethane (50 cm^3) was stirred at room temperature until all the solid had dissolved (*ca.* 30 mins). The red solution was filtered, then concentrated until the product began to precipitate. Addition of a two-fold excess volume of diethyl ether afforded the product as a bright orange powder, which was collected by filtration, washed with diethyl ether and dried *in vacuo*. The other complex salts were prepared by analogous reactions using the appropriate iron precursor $Fe[ClO_4]_2 \cdot 6H_2O$ or $Fe[CF_3SO_3]_2$; $Fe[PF_6]_2$ was prepared *in situ* using stoichiometric quantities of $FeCl_2 \cdot 4H_2O$ and $AgPF_6$. Crude yields were 55-65 %.

The isolated iron complex salts are sparingly soluble in common solvents, but can be recrystallized in small quantities from nitromethane. More crystalline products were obtained using di-*iso*propylether, rather than diethyl ether, as the antisolvent in the crystallizations.

For $[FeL_2][BF_4]_2$. Orange solid. Elemental analysis: calcd (%) for $C_{24}H_{16}B_2F_8FeN_{12}$ (701.96) C 41.1, H 2.30, N 24.0; found C 40.9, H 2.17, N 23.8; IR no νC≡N vibration observed (Figure S25).

For $[FeL_2][ClO_4]_2$. Red solid. Elemental analysis: calcd (%) for $C_{24}H_{16}Cl_2FeN_{12}O_8$ (727.24) C 39.6, H 2.22, N 23.1; found C 39.4, H 1.80, N 22.7; IR 2238 cm^{-1} (ν{C≡N}).

For $[FeL_2][PF_6]_2$. Red solid. ^1H NMR (CD_3CN) δ = 24.0, 27.3 (both 4H, Py $H^{3/5}$ *&* Pz H^5), 41.3, 43.0 (both 4H, Pz H^3 + H^4); elemental analysis: calcd for $C_{24}H_{16}F_{12}FeN_{12}P_2$ (818.28) C 35.2, H 1.97, N 20.5; found C 35.2, H 1.86, N 20.2; IR 2238 cm^{-1} (ν{C≡N}).

For $[FeL_2][CF_3SO_3]_2$. Red solid. Elemental analysis: calcd (%) for $C_{26}H_{16}F_6FeN_{12}O_6S_2$ (826.48) C 37.8, H 1.95, N 20.3; found C 37.7, H 1.86, N 20.3; IR 2238 cm^{-1} (ν{C≡N}).

Synthesis of [Ag(μ-L)]BF_4. Solid L (0.050 g, 0.21 mmol) was dissolved in nitromethane (10 cm^3). The solution was heated to 40 °C, and $AgBF_4$ (0.040 g, 0.21 mmol) was then added. The solution was stirred at this temperature for 2 hours, then filtered. Slow diffusion of diethyl ether vapor into the yellow solution yielded the product as a white powder. The freshly crystallized material contains 0.5 equiv nitromethane by X-ray diffraction, but microanalysis implies this is replaced by a similar amount of lattice water upon drying in air. Yield 0.059 g, 64 %. Elemental analysis: calcd (%) for $C_{12}H_8AgBF_4N_6 \cdot 0.5H_2O$ (430.91) C 32.8, H 2.06, N 19.1; found C 32.7, H 1.52, N 18.6; IR 2271 cm^{-1} (ν{C≡N}).

Single crystal *X*-ray structures

The crystals of L were obtained from an NMR sample solution of the compound in $CDCl_3$, while the metal complex crystals were obtained by slow diffusion of di-*iso*propyl ether vapor into solutions of those compounds in nitromethane. Diffraction data for the HS1 and HS2 phases of $[FeL_2][BF_4]_2$ were measured with a Bruker D8 Diffractometer with a IμS Microfocus Cu-Kα sealed tube source and a Photon III detector. Other data were collected using an Agilent SuperNova dual source diffractometer, with Cu-K_α (λ = 1.5418 Å) radiation. Experimental data for the crystal structures are listed in Table S1.

The structures were solved by direct methods (*SHELX-TL*[2]), and developed by full least-squares refinement on F^2 (*SHELXL2018*[3]). Crystallographic figures were produced using *XSEED*,[4] and other publication materials were prepared with *OLEX*2.[5] Unless otherwise stated, all crystallographically ordered non-H atoms in the structures described below were refined anisotropically, and H atoms were placed in calculated positions and refined using a riding model.

Structure refinement of L. The asymmetric unit of L contains half a molecule of the compound, which spans a crystallographic C_2 axis. H atoms in this structure were located in the Fourier map and refined positionally, with U_{iso} constrained to 1.2xU_{eq} of the bound C atom.

Structure refinement of $[FeL_2][BF_4]_2$. This material crystallizes as stacks of thin platelets, which were challenging to separate for analysis.

A survey of five crystals at room temperature identified two different high-spin phases of the material with triclinic (HS1) and tetragonal (HS2) symmetry. These both diffract weakly at 300 K, and a θ = 55° resolution cutoff was applied to the HS1 data. The HS1 crystal described here was refined as a two-component twin by rotation around *c* (as for LS3 below), while the HS2 crystal was treated as a two-component merohedral twin. Anion disorder in each crystal was modelled using refined B–F and F···F distance restraints. While their basic structural features are clear, the refinements have low precision and high residuals.

The HS1 and HS2 crystals were both analyzed on a 300, 250, 200, 150, 250, 275 and 300 K temperature cycle. Their diffraction quality declined further on cooling, and was particularly poor following the HS-LS transition. None-the-less the different phases adopted by these crystals at each temperature were clearly defined (Tables S5 and S6).

After many attempts a higher quality dataset was obtained of the low-spin phase (LS3) of the compound, containing two non-superimposable twin domains related by an almost perfect 180° rotation about *c*. The twinning was successfully resolved, which yielded a precise refinement of this phase. The asymmetric unit contains two formula units of the compound, with all residues in general crystallographic positions. No positional disorder is present in the model, but a displacement ellipsoid restraint was applied to one C atom to address a non-positive-definite error. A second data collection from the same crystal at 100 K yielded the HS1 unit cell, but was too weak to be solved.

Structure refinements of $[FeL_2][ClO_4]_2$ and $[FeL_2][PF_6]_2$. These isomorphous crystals were collected at 120 and 125 K, respectively. There is no disorder in the perchlorate salt, and no restraints were applied to its refinement. One anion in $[FeL_2][PF_6]_2$ is disordered over two positions, with occupancies of 0.67 and 0.33. These were treated with refined P–F and *trans*-F···F distance restraints. All non-H atoms except the minor anion disorder site were refined anisotropically in that structure. A room temperature crystal structure of $[FeL_2][ClO_4]_2$ is described in ref. [1].

Structure refinement of $[FeL_2][CF_3SO_3]_2$. No disorder is present in the refinement of this crystal, which has no special features.

Structure refinement of $[Ag(\mu\text{-}L)]BF_4{\cdot}½MeNO_2$. The asymmetric unit contains two silver ions and two organic ligands forming part of the same coordination polymer chain; two BF_4^- ions; and one molecule of nitromethane. No disorder is present in the model, and no restraints were applied to the refinement. There is a residual Fourier peak of +1.4 e Å^{-3} which may represent minor unresolved disorder in the solvent molecule.

Deposition numbers 2375310 (L), 2375311 ($[FeL_2][BF_4]_2$, LS3), 2375312 ($[FeL_2][ClO_4]_2$), 2375313 ($[FeL_2][PF_6]_2$), 2375314 ($[FeL_2][CF_3SO_3]_2$), 2375315 ($[Ag(\mu\text{-}L)]BF_4{\cdot}½MeNO_2$), 2380639 ($[FeL_2][BF_4]_2$, HS1) and 2380640 ($[FeL_2][BF_4]_2$, HS2) contain the supplementary crystallographic data for this paper. These data are provided free of charge by the joint Cambridge Crystallographic Data Centre and Fachinformationszentrum Karlsruhe Access Structures service.

Other measurements

Elemental analyses were performed by the London Metropolitan University School of Human Sciences microanalytical service. Electrospray mass spectra were recorded on a Bruker MicroTOF-q instrument from $CHCl_3$ solution. Diamagnetic NMR spectra employed a Bruker AV3HD spectrometer operating at 400.1 (^{1}H) or 100.6 MHz (^{13}C), while paramagnetic ^{1}H NMR spectra were obtained with a Bruker AV3 spectrometer operating at 300.1 MHz. IR spectra were obtained with a Bruker Alpha instrument.

Magnetic susceptibility measurements were performed using a Quantum Design MPMS-3 SQUID/VSM magnetometer, in an applied field of 5000 G. Polycrystalline powder samples were measured inside a plastic capsule mounted in a brass rod holder, at a scan rate of 5 K min^{-1} unless otherwise stated. Diamagnetic corrections for the samples were estimated from Pascal's constants,[6] and a previously measured diamagnetic correction for the sample holder was also applied to the data. Differential scanning calorimetry (DSC) measurements were performed with a TA Instruments Q2000 heat flux calorimeter with a TA RCS90 refrigerated cooling system. The samples were prepared in aluminium, hermetically sealed pans and scanned at a rate of 10 K min^{-1}.

Room temperature X-ray powder diffraction data were measured with a Bruker D2 Phaser diffractometer, using Cu-K_α radiation (λ = 1.5419 Å). Unless otherwise stated, variable temperature powder diffraction was performed using a Bruker D8 Advance X-ray powder diffractometer equipped with a Cu-K_α sealed-tube source, a focusing Göbel Mirror and a Lynx-Eye Detector. The sample was measured inside a 0.7 mm borosilicate glass capillary. The temperature was controlled with a Oxford Cryosystems Cryostream 700 Plus Series. Data were collected at a series of temperatures with a ramp rate of 360 K/hour with a 300 s dwell period prior to data collection to ensure suitable thermal equilibrium. Data were collected for 19 minutes with a scan range $4 \leq 2\theta \leq 60°$ (step size of 0.015°, sample rotation 30 rpm). The collected data were analysed using Pawley refinement methods.[7]

A second variable temperature cycling experiment was then performed on a second sample from the same crystallization vial. A temperature series was collected at 300, 250, 180, 235, 250 and 275 K which was repeated four times. Data were collected for 19 minutes (250, 235, 250 and 275 K) or 58 minutes (300 and 180 K), with each collection preceded by a 5-minute dwell period to allow for thermal equilibration. Data were initially analysed using Pawley refinement[7] then, in order to determine the phase composition of the high-spin and low-spin samples, data recorded at 300 and 180 K were re-analysed using Rietveld methods.[8] The crystallographic structures for HS1, HS2 and LS3 were used as initial models, with atom coordinates, thermal displacement and occupancy of the disordered BF_4^- ions being fixed to the single crystal values. An initial model of the LS4 phase was produced from the synchrotron powder diffraction data, as described below. Lattice parameters, peaks shapes, background parameters and zero point error were refined starting from parameters taken from the preliminary Pawley fitting.

Synchrotron powder diffraction data were obtained from station I11 at Diamond Light Source, using synchrotron radiation (λ = 0.8241 Å).[9-11] Data were recorded using a wide-angle (90°) position sensitive detector (PSD) comprising of 18 Mythen-2 modules.[11] Data from two different sample of $[FeL_2][BF_4]_2$ were collected on a 300→150→300 K temperature cycle. The samples were held at each temperature for 500 s then collected over the range $2.0 \leq 2\theta \leq 66.4°$, with a resolution of 0.04° and a collection time of 30 s. A structural model of LS4 was obtained by Rietveld refinement of the data with $2\theta \leq 40°$ using rigid body cation and anion moieties generated from LS3, whose positions and rotations were allowed to freely refine. This model was used for the low-spin Rietveld refinements in the thermal cycling experiment.

The Pawley and Rietveld refinements were performed using *Topas* in conjunction with *jEdit*.[12-14]

Table S1. Experimental data for the crystal structure determinations.

	L	$[FeL_2][BF_4]_2$			$[FeL_2][ClO_4]_2$	$[FeL_2][PF_6]_2$	$[FeL_2][CF_3SO_3]_2$	$[Ag(\mu\text{-}L)]BF_4\cdot$-½$MeNO_2$
		HS1[c]	HS2[c]	LS3[c,d]				
formula	$C_{12}H_8N_6$	$C_{24}H_{16}B_2F_8FeN_{12}$			$C_{24}H_{16}Cl_2FeN_{12}O_8$	$C_{24}H_{16}F_{12}FeN_{12}P_2$	$C_{26}H_{16}F_6FeN_{12}O_6S_2$	$C_{12.5}H_{9.5}AgBF_4N_{6.5}O$
fw	236.24	701.96			727.24	818.28	826.48	461.45
crystal system	monoclinic	triclinic	tetragonal	triclinic	monoclinic	monoclinic	Monoclinic	triclinic
space group	$C2/c$	$P\bar{1}$	$P4_3$	$P\bar{1}$	$P2_1/c$	$P2_1/c$	$P2_1/n$	$P\bar{1}$
a [Å]	14.2930(12)	8.4864(13)	8.4905(4)	8.5664(6)	18.9858(8)	19.4196(3)	12.1782(1)	9.8180(10)
b [Å]	9.8176(5)	8.5043(12)	8.4905(4)	16.7521(8)	8.6768(4)	8.7930(1)	20.3824(2)	12.1024(11)
c [Å]	8.4088(6)	19.871(3)	39.283(3)	19.3801(19)	17.0037(7)	17.4105(3)	12.7052(2)	13.8688(13)
α [deg]	90	81.785(11)	90	95.278(6)	90	90	90	97.690(8)
β [deg]	107.163(7)	87.899(10)	90	90.487(7)	91.847(4)	92.682(1)	96.350(1)	110.021(9)
γ [deg]	90	89.859(9)	90	91.814(5)	90	90	90	94.937(8)
V [Å^3]	1127.40(14)	1418.4(4)	2831.9(3)	2767.8(4)	2799.7(2)	2969.70(8)	3134.35(6)	1518.9(3)
Z	4	2	4	4	4	4	4	4
T [K]	120(2)	300(2)	300(2)	120(2)	120(2)	125(2)	120(2)	120(2)
D_{calcd} [g cm^{-3}]	1.392	1.644	1.646	1.685	1.725	1.830	1.751	2.018
μ\{Cu-K_α\} [mm^{-1}]	0.753	5.120	5.129	5.248	6.737	6.193	6.021	11.269
reflections measured	1927	9034	42012	12882	12448	11397	13823	11255
unique reflections	1082	3303	4817	12882	5288	5789	5954	5695
R_{int}	0.016	0.188	0.113	–[d]	0.056	0.031	0.022	0.037
R_1 [$I > 2\sigma(I)$][a]	0.031	0.162	0.145	0.074	0.065	0.044	0.032	0.038
wR_2 [all data][b]	0.087	0.447	0.403	0.210	0.157	0.116	0.083	0.091
GOF	1.082	1.485	1.811	0.958	1.062	1.202	1.023	1.038
Flack parameter	–	–	0.718(4)[c]	–	–	–	–	–
CCDC	2375310	2380639	2380640	2375311	2375312	2375313	2375314	2375315

[a] $R = \Sigma [|F_o| - |F_c|] / \Sigma |F_o|$ [b] $wR = [\Sigma w(F_o^2 - F_c^2)^2 / \Sigma wF_o^4]^{1/2}$ [c] Refined as a two-component twin. [d] The non-superimposable twin domains in this dataset were resolved in the hkl file, whose reflections were treated without merging.

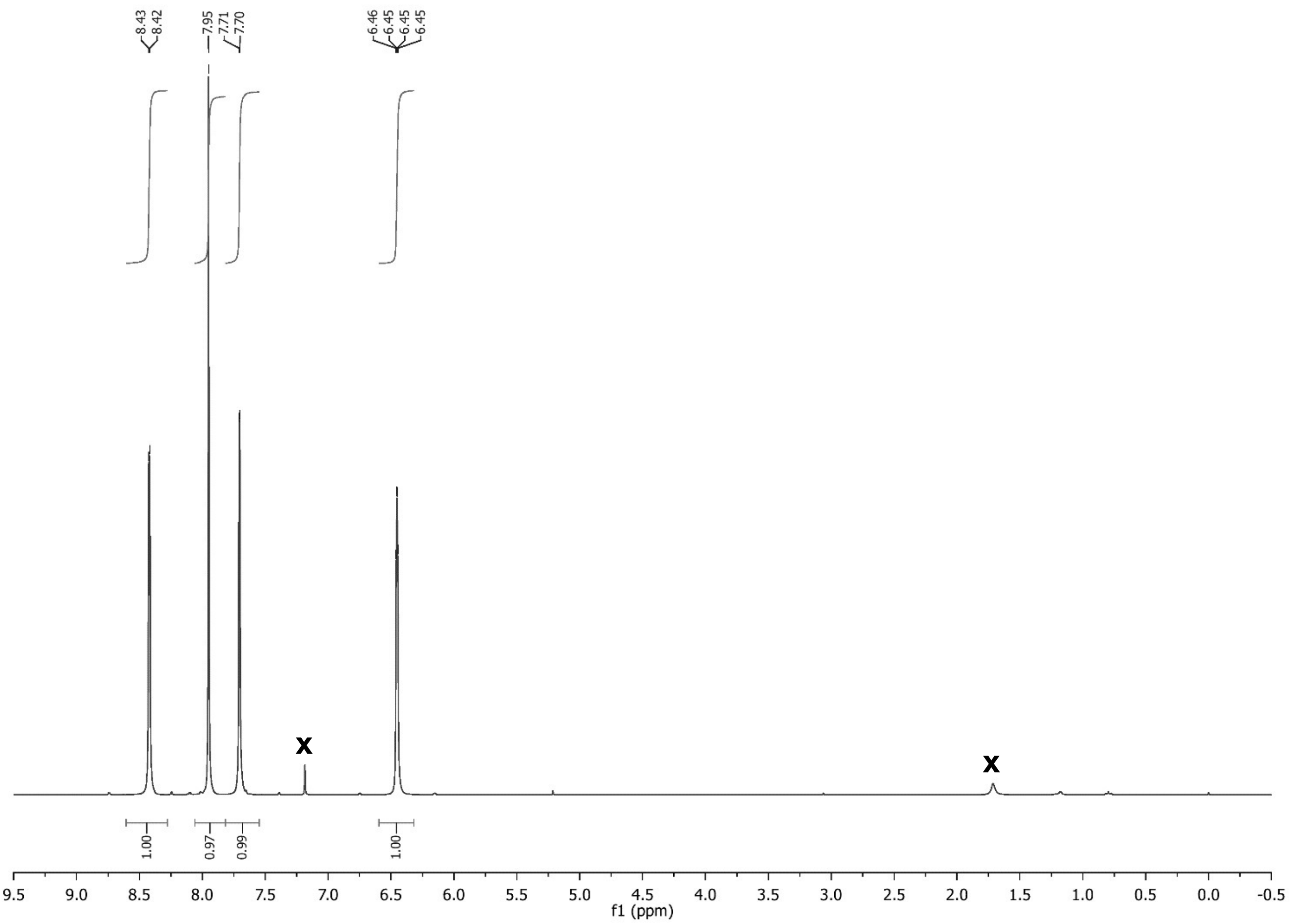

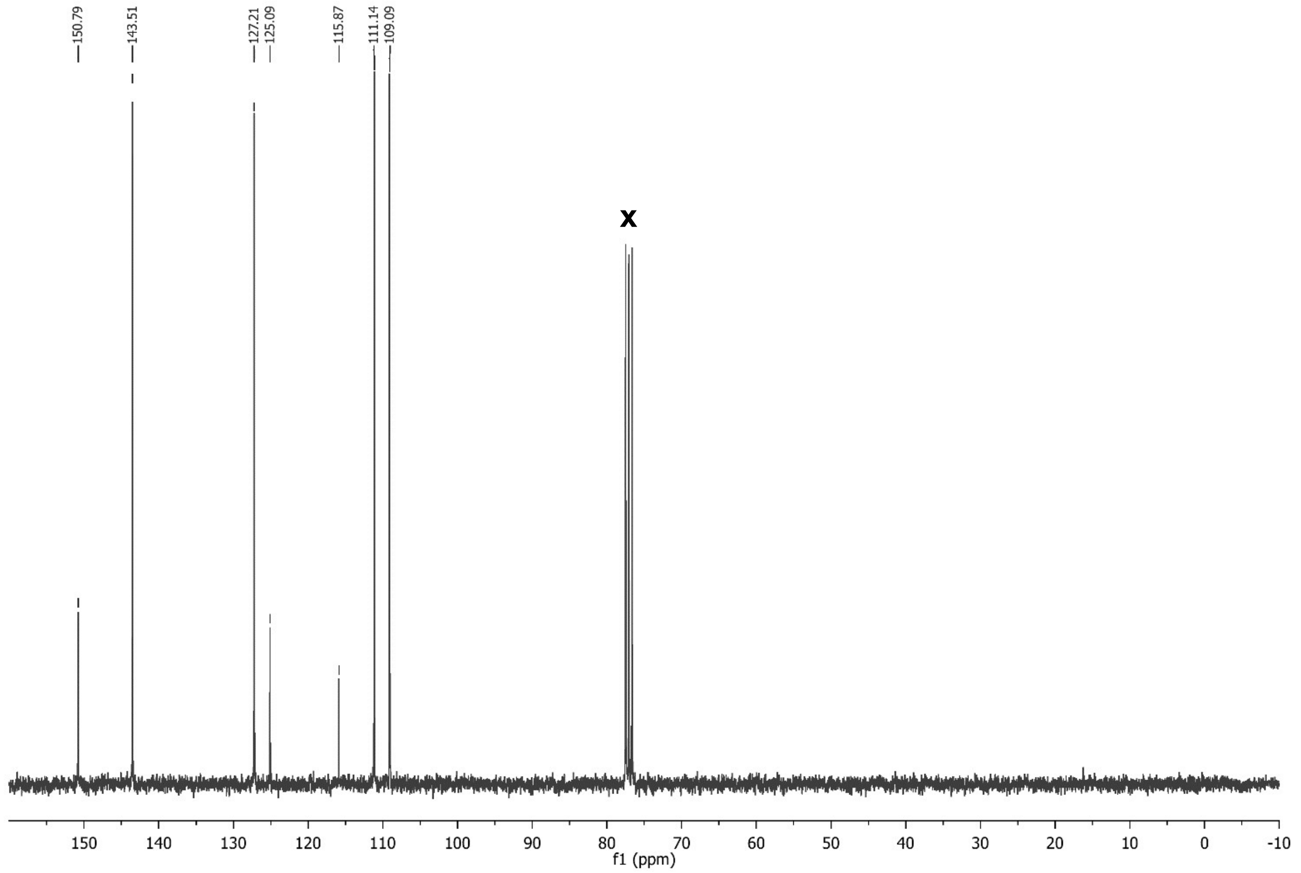

Figure S1 ^{1}H and ^{13}C NMR spectra of L ($CDCl_3$).[1]

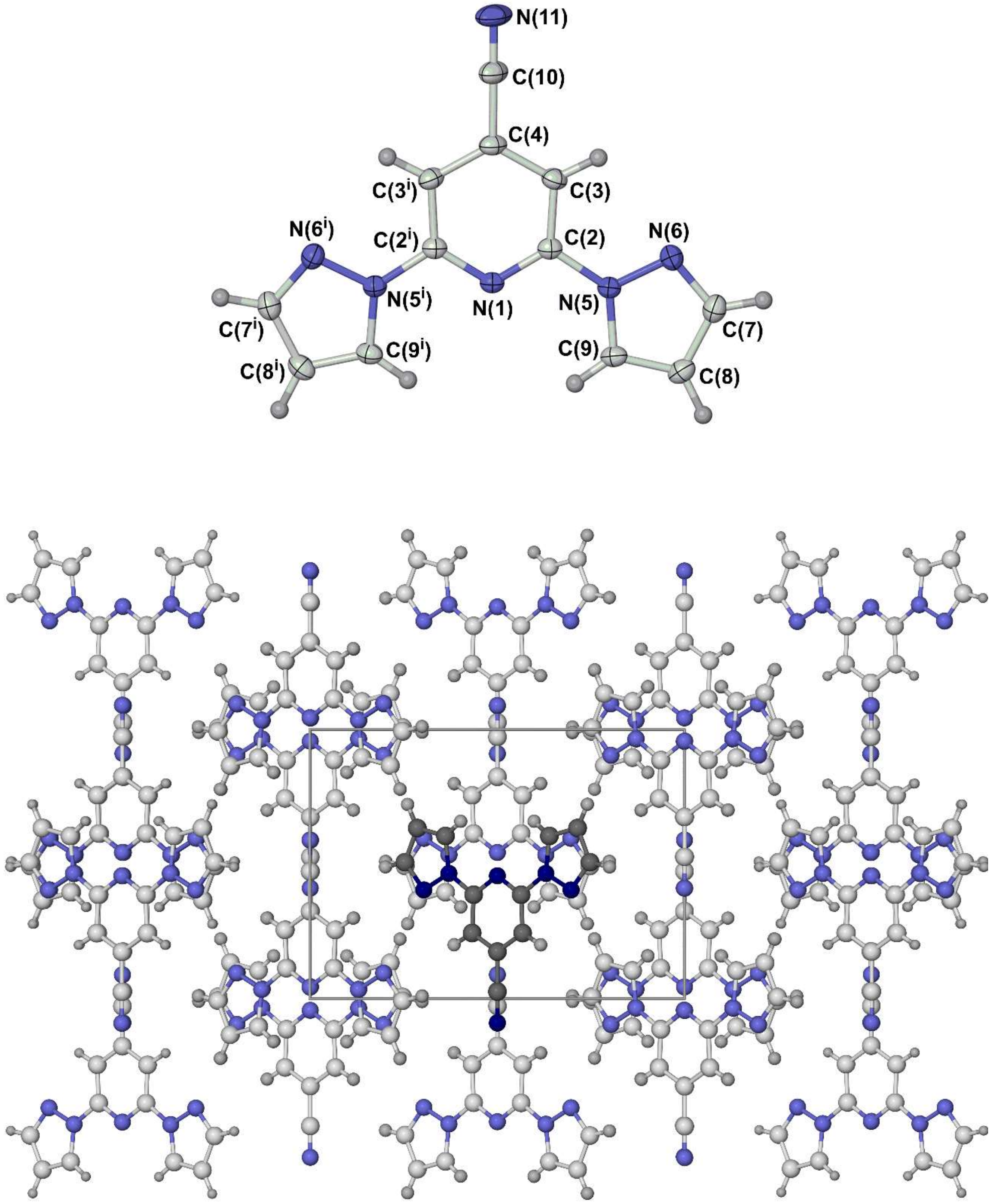

Figure S2 Top: view of the molecule in the crystal structure of L. Displacement ellipsoids are at the 50 % probability level except for H atoms which have arbitrary radii. Bottom: packing diagram of L, viewed parallel to the [001] crystal vector with *a* horizontal. One molecule in the diagram is highlighted with dark coloration, and all atoms in this view have arbitrary radii.

Symmetry code: (i) 1–*x*, *y*, $^{3}/_{2}$–*z*. Color code: C, white or dark gray; H, pale gray; N, pale or dark blue.

Molecules of *L* pack through can der Waals contacts into layers oriented in the $(10\overline{1})$ plane. Molecules in adjacent layers are related by crystallographic inversion symmetry and thus are coplanar, and separated by an interplanar distance of 3.246(2) Å.

Angular Distortion Parameters Used to Describe the Molecular Geometries of $[FeL_2]^{2+}$ Salts.

θ is the dihedral angle between the least squares planes of the two L ligands in a mononuclear $[FeL_2]^{2+}$ complex, while ϕ is the *trans*-N{pyridyl}–Fe–N{pyridyl} bond angle (Scheme S1).[15,16] These parameters are often significantly lower than their ideal values in high-spin $[Fe(bpp)_2]^{2+}$ derivatives and related complexes (*ie* $\theta < 90$ and/or $\phi < 180°$).[17,18]

Large changes in ϕ, particularly, between the spin states can lead to highly cooperative spin-transitions.[19-22] However, spin-crossover can be inhibited if θ and ϕ deviate more strongly from their ideal values in the high-spin state,[16,18] because the associated rearrangement to a more regular low-spin coordination geometry ($\theta \approx 90°$, $\phi \approx 180°$) cannot be accommodated by a rigid solid lattice.[23]

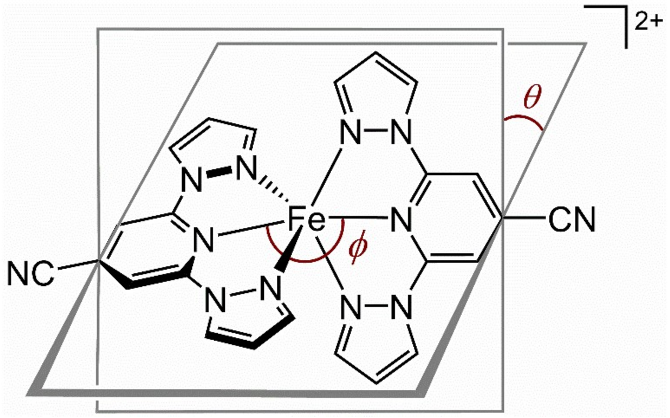

Scheme S1 θ and ϕ, used to discuss the structures of $[FeL_2]^{2+}$ and other $[Fe(bpp)_2]^{2+}$ derivatives.[15-18]

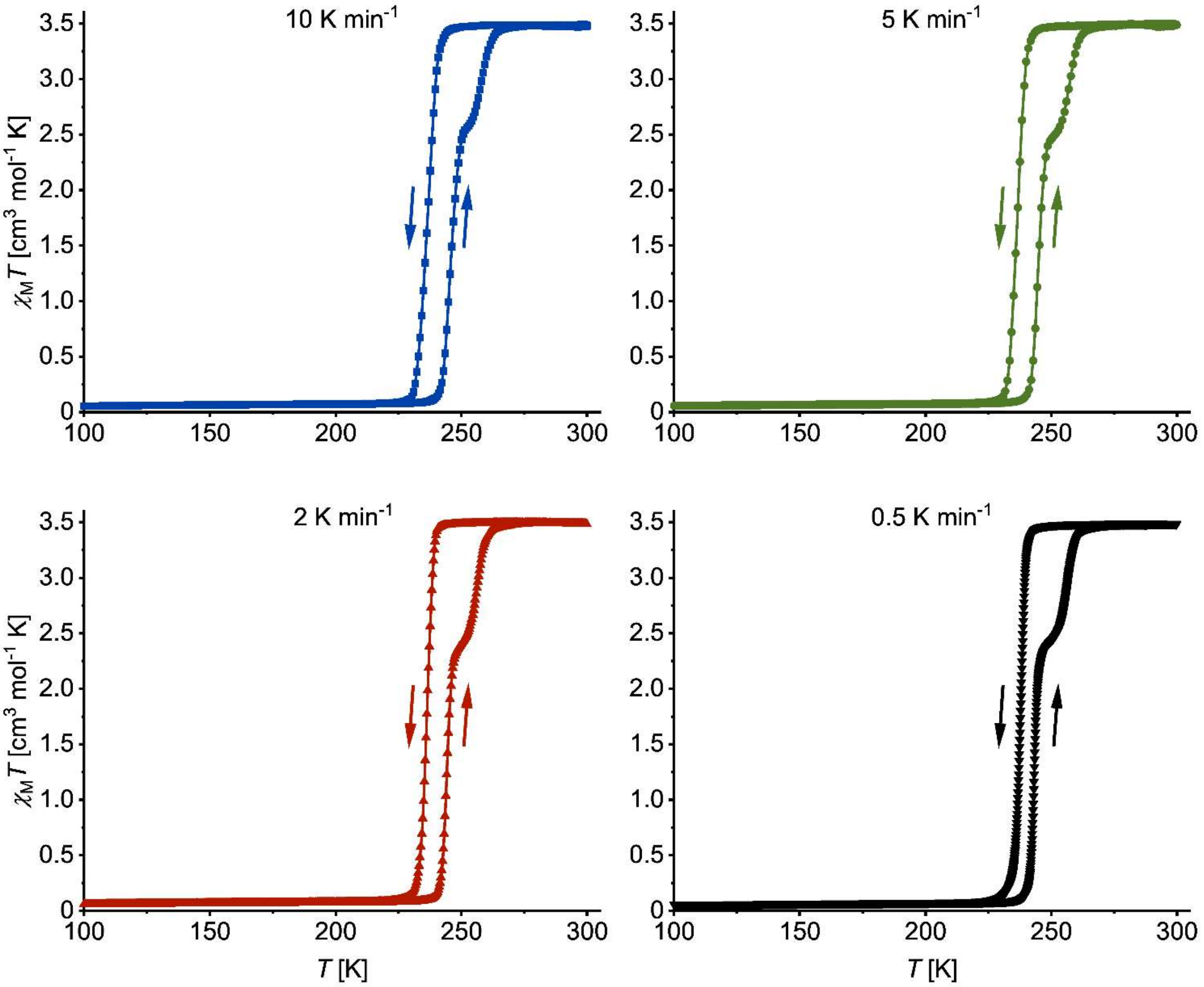

Figure S3 Magnetic susceptibility data for polycrystalline $[FeL_2][BF_4]_2$ at different thermal scan rates (Figure 1, main article). Four different samples from the same batch of material were measured in cooling and heating modes. The data are reproduced from Figure 1(a) and are color-coded as in that Figure, with datapoints connected by spline curves for clarity.

The transition parameters range between $T_{½}\downarrow$ = 238 K and $T_{½}\uparrow$ = 244, 256 K at 0.5 K min^{-1}; and, $T_{½}\downarrow$ = 236 K and $T_{½}\uparrow$ = 247, 258 K at 10 K min^{-1}. That represents an unusually small temperature dependence for a first order, hysteretic spin-transition.[24]

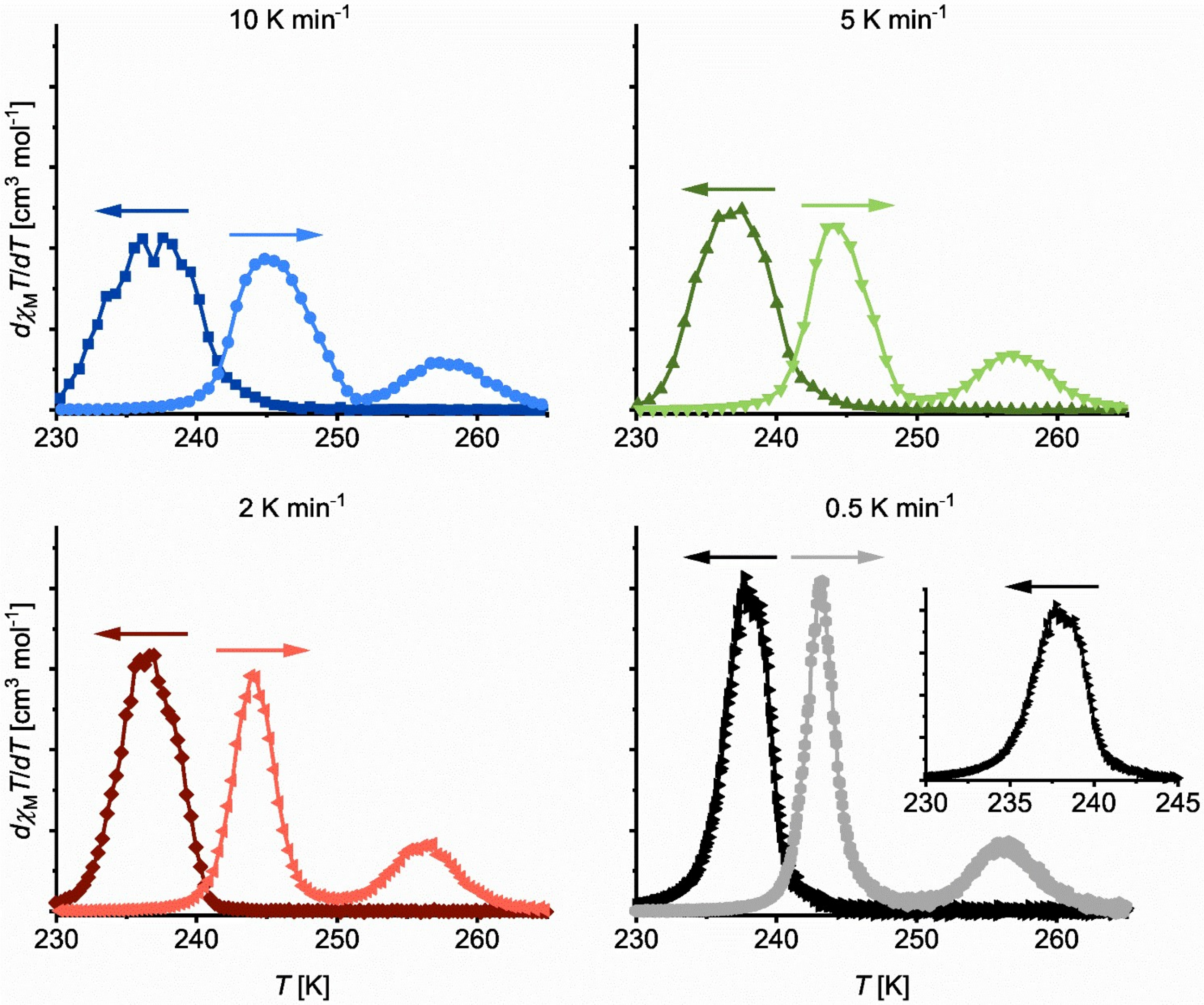

Figure S4 First derivative plots of the magnetic susceptibility data in Figure S3. An expansion of the cooling scan at 0.5 K min^{-1} is shown.

Although the data are sparser at faster scan rates, the cooling branch of each scan has structure which is not apparent in the raw data. This is clearest in the 0.5 K min^{-1} measurement, where the high→low-spin transition appears to be split into two closely-spaced components. That is consistent with the existence of two structural phases in the high-spin sample, undergoing SCO at (almost) identical temperatures on cooling.

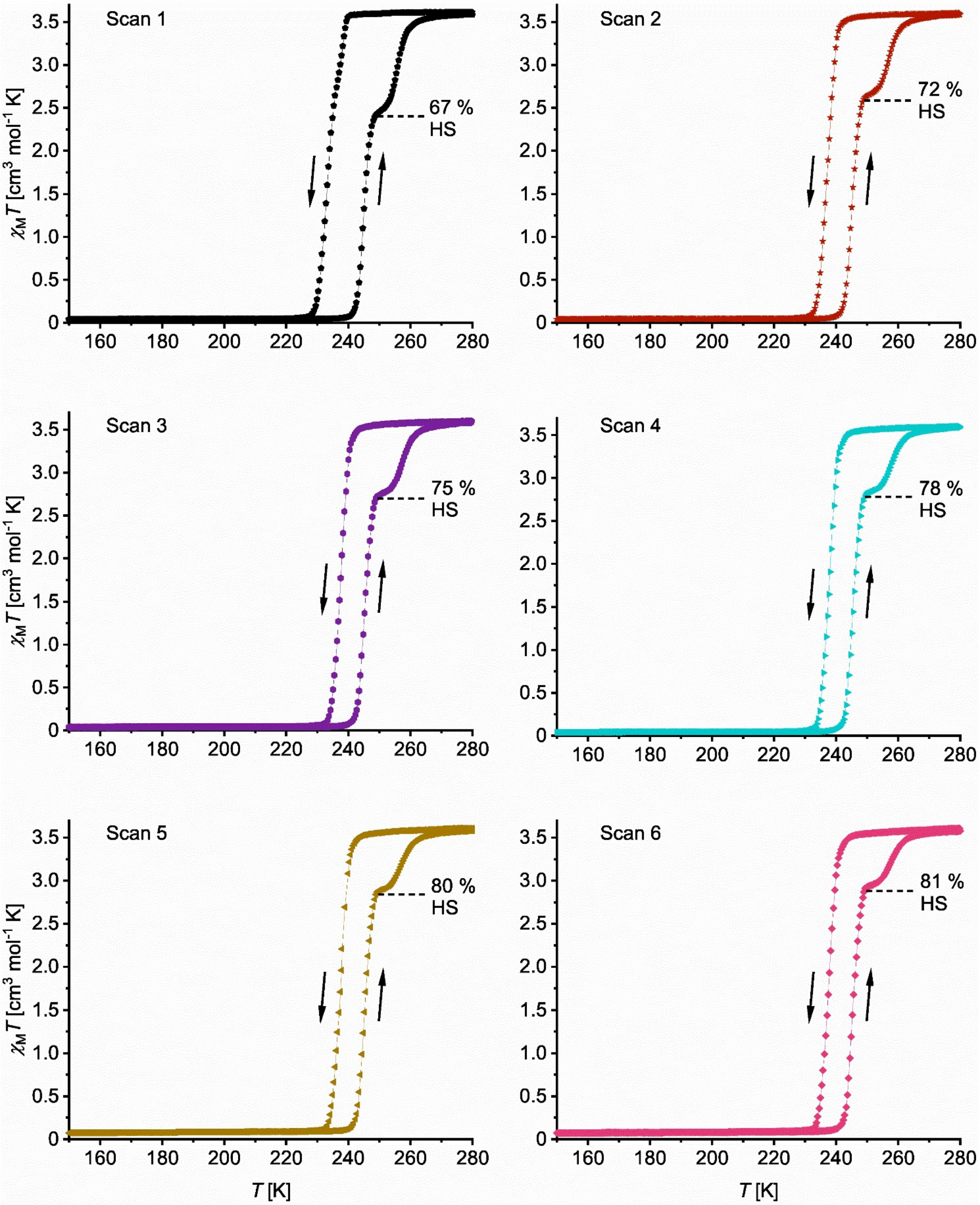

Figure S5 Magnetic susceptibility data for the same sample of polycrystalline $[FeL_2][BF_4]_2$, measured across ten 300→150→300 K thermal cycles at scan rate 5 K min^{-1}. The data are reproduced from Figure 1(b) and are color-coded as in that Figure, with datapoints connected by spline curves for clarity.

The high-spin fraction at the onset of the step in the warming curves is shown, which is a measure of the phase composition of the sample in each scan. These values are plotted in Figure 7 of the main article.

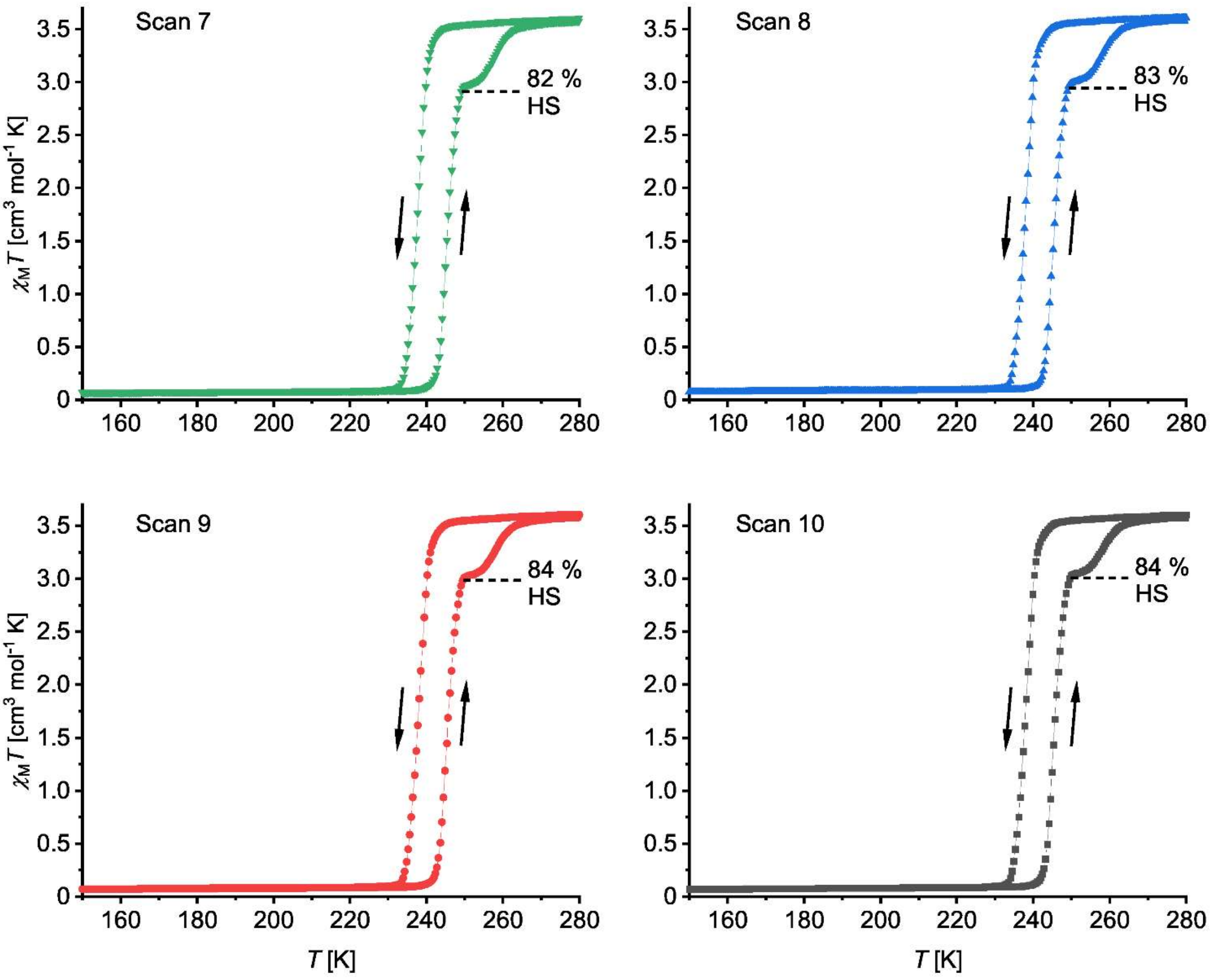

Figure S5 continued.

There are two notable aspects of these data. Firstly, the hysteresis on scan 1 is 5 K wider with a more pronounced shoulder on the cooling branch, than for other samples measured in this work ($T_{½}$↓ = 233, 238 K and $T_{½}$↑ = 245, 256 K). That suggests this sample contained larger or better quality crystallites than the other samples investigated in this study.[24] In scans 2-10 the data revert to the narrower hysteresis observed in Figure S3 for this scan rate ($T_{½}$↓ = 238 K and $T_{½}$↑ = 245, 257 K).

Secondly the intensity of the shoulder on the warming branch of the hysteresis loop decreases with each thermal scan. It reflects the enrichment of HS1/LS3 over the HS2/LS4 phases as the sample is cycled repeatedly about its spin transition, which was observed in the powder diffraction data.

A multiple scanning magnetic measurement is also reported in ref. [1], which shows the same trends as in this Figure. Scan 1 in this Figure is equivalent to sample **1a** in ref. [1], whereas at the end of the measurement the data resemble their sample **1b**.

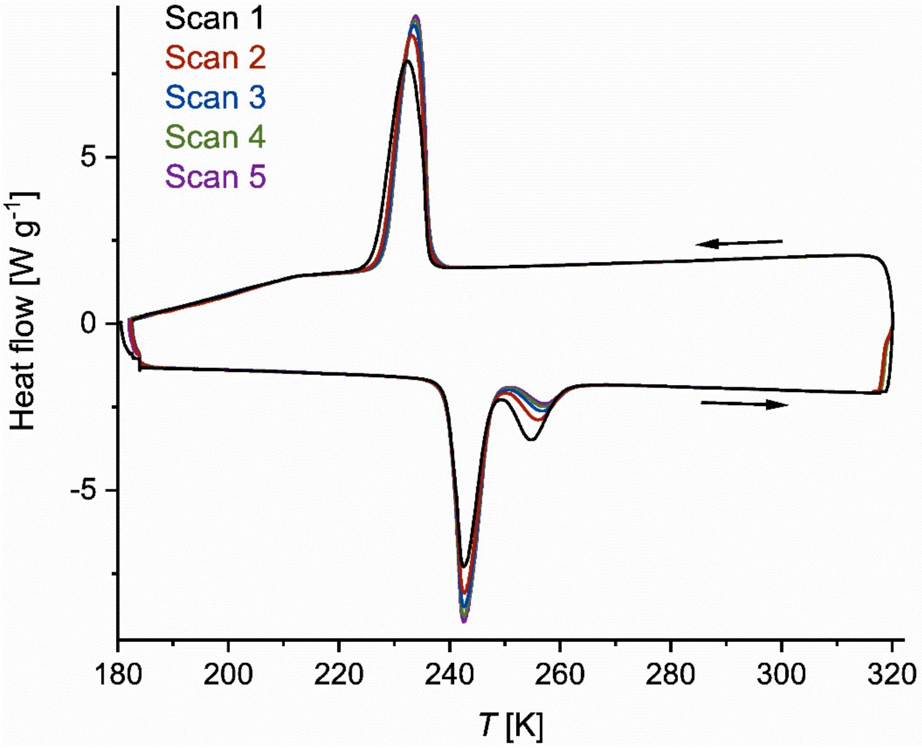

Figure S6 Differential scanning calorimetry (DSC) data for polycrystalline $[FeL_2][BF_4]_2$. The sample was cycled five times about its SCO transition, at a scan rate of 10 K min^{-1}.

A DSC measurement is also reported in ref. [1] which shows the same trends as in this Figure, but was not analyzed in detail.

Table S2 SCO parameters from the DSC data in Figure S6. The composition of the sample in each scan (% HS1) is estimated as the ratio of ΔH for peak 1 on the heating scan, against ΔH for the cooling mode peak.

	Heating						Cooling			
	Peak 1			Peak 2						
Cycle	*T* [K]	ΔH [kJ mol^{-1}]	Δ*S* [J mol^{-1} K^{-1}]	*T* [K]	ΔH [kJ mol^{-1}]	Δ*S* [J mol^{-1} K^{-1}]	*T* [K]	ΔH [kJ mol^{-1}]	Δ*S* [J mol^{-1} K^{-1}]	% HS1[a]
1	245.5	9.15	37.3	258.4	2.99	11.6	235.4	14.90	63.3	61.4
2	245.6	11.05	45.0	259.6	1.93	7.4	236.2	14.87	63.0	74.2
3	245.6	12.02	48.9	260.3	1.52	5.8	236.5	14.93	63.1	80.7
4	245.5	12.57	51.2	260.2	1.29	5.0	236.7	14.91	63.0	84.4
5	245.6	13.05	53.1	260.3	1.10	4.2	236.9	14.99	63.3	87.6

[a] The % HS1 data are included in Figure 7 of the main article.

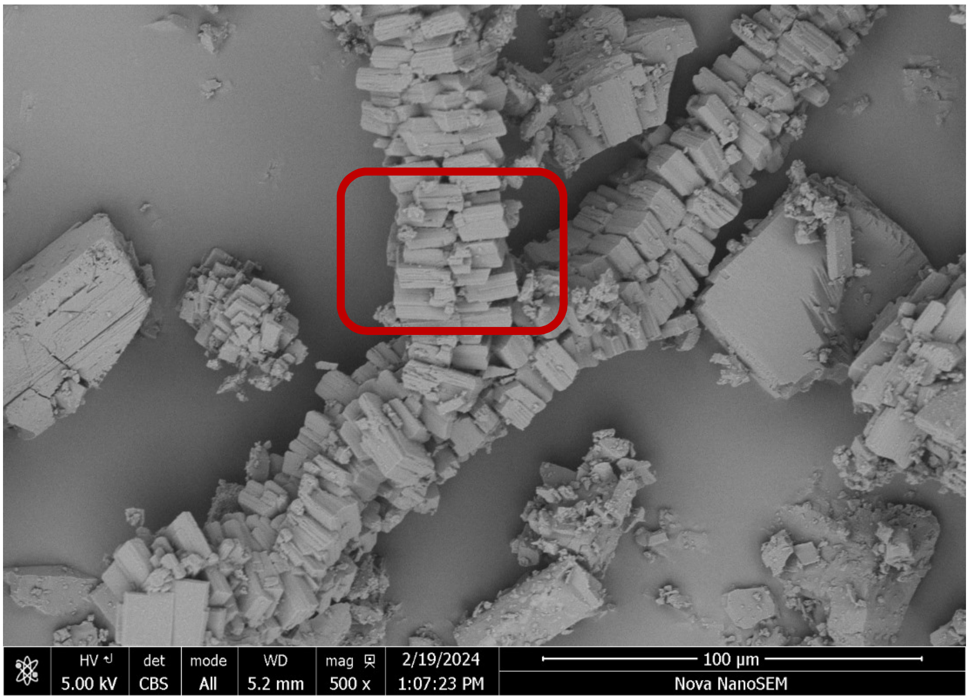

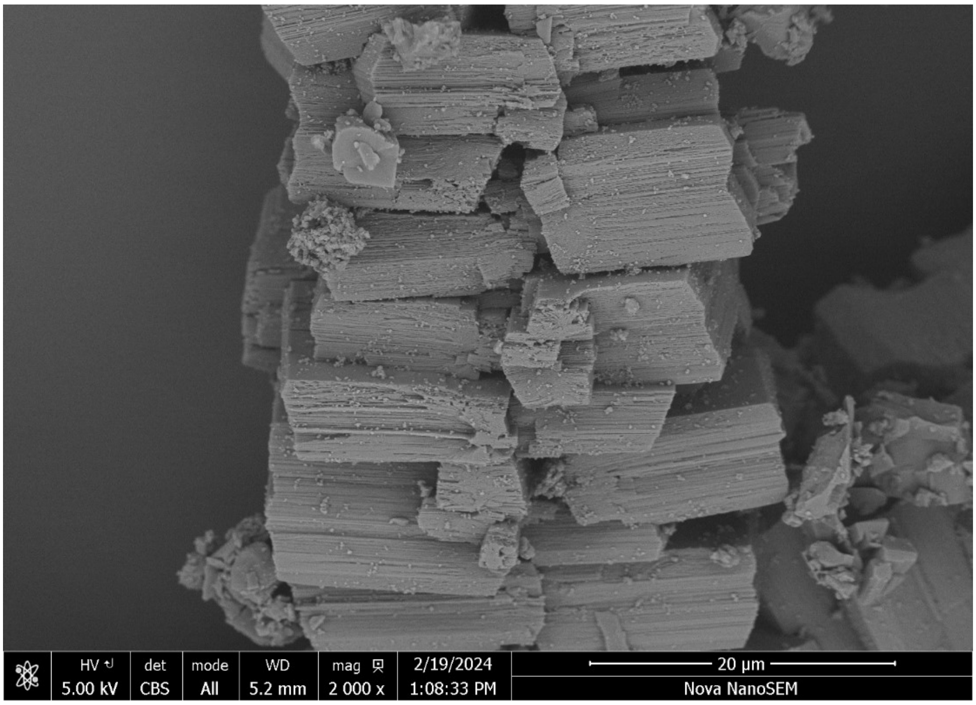

Figure S7 Scanning electron microscope images of a typical polycrystalline sample of $[FeL_2][BF_4]_2$. The bottom image is an expansion of the highlighted area in the top image, showing that the apparently three-dimensional crystals are composed of stacks of thin plates.

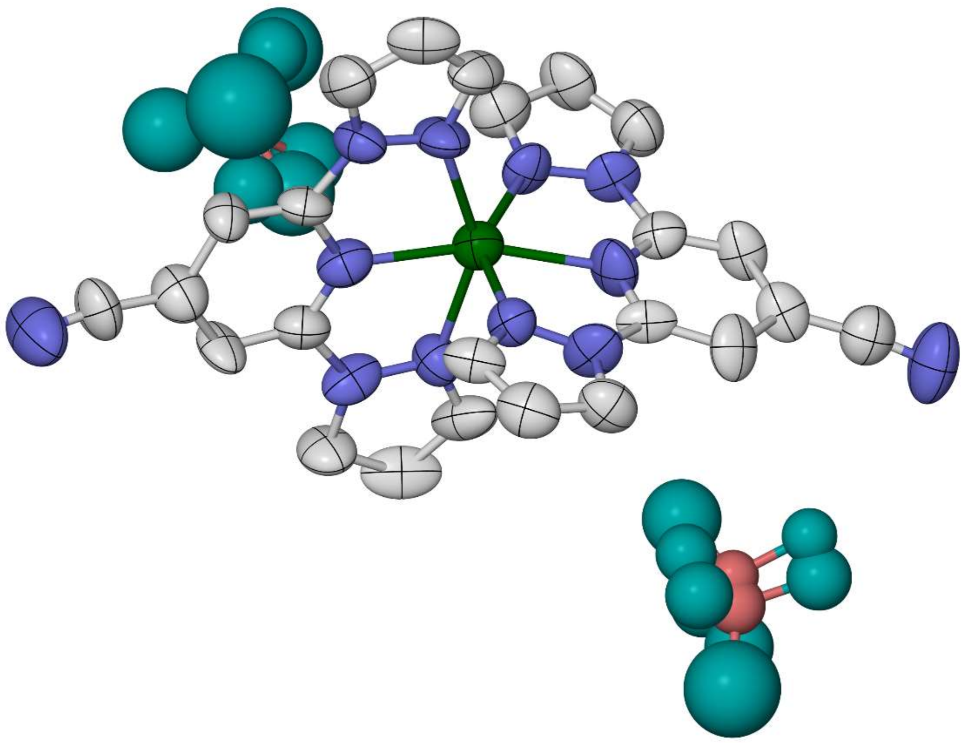

Figure S8 The asymmetric unit of phase HS1 of $[FeL_2][BF_4]_2$ at 300 K. Displacement ellipsoids are at the 50 % probability level, and H atoms are omitted for clarity. Anion disorder is included in the Figure.

Color code: C, white; B, pink; F, cyan; Fe, green; N, blue.

The complex is high-spin at this temperature. While the analysis has low precision, the iron coordination geometry in this phase is significantly distorted from ideal D_{2d} symmetry [ϕ = 161.3(7)°, θ = 84.20(17)°; page S9]. Cooperative spin transitions in $[Fe(bpp)_2]^{2+}$ derivatives are often associated with 'ϕ' distortions of this type in the high-spin state.[16-19]

Complete diffraction data were recorded from this crystal at 300, 250, 200, 150, 250, 275 and 300 K, at a temperature ramp rate of 120 K hr^{-1} with a 5 minute dwell time prior to data collection to ensure thermal equilibration. Diffraction quality was poor particularly following the HS-LS transition, resulting in resolution cutoffs being applied based on diffraction quality. In spite of this poor quality, it was possible to resolve the HS1 and LS3 phases from this crystal over the complete temperature range (Table S5, below).

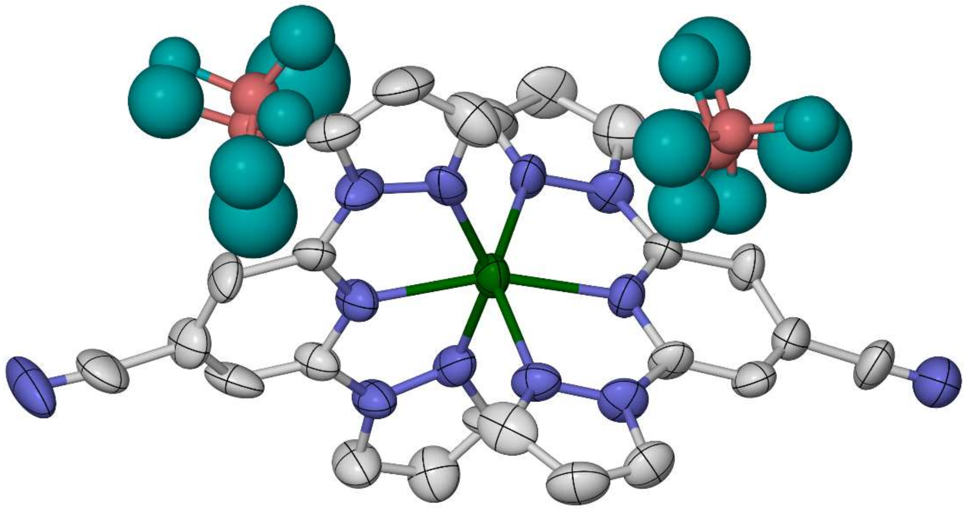

Figure S9 The asymmetric unit of phase HS2 of $[FeL_2][BF_4]_2$ at 300 K. Displacement ellipsoids are at the 50 % probability level, and H atoms are omitted for clarity. Anion disorder is included in the Figure.

Color code: C, white; B, pink; F, cyan; Fe, green; N, blue.

The iron complex in HS2 is also high-spin, with a similar coordination geometry to HS1 [ϕ = 160.6(6)°, θ = 84.53(19)°; page S9].

This crystal was also measured with the same variable temperature protocol as for HS1. While the existence of the HS2→LS4 transformation was clear, it was challenging to structurally resolve LS4, leading to only lattice parameters being derived for that phase at 200, 150 and 250 K (Table S6, below). The final temperature measurements 275 and 300 K from this crystal resolved as the HS1 phase, not HS2.

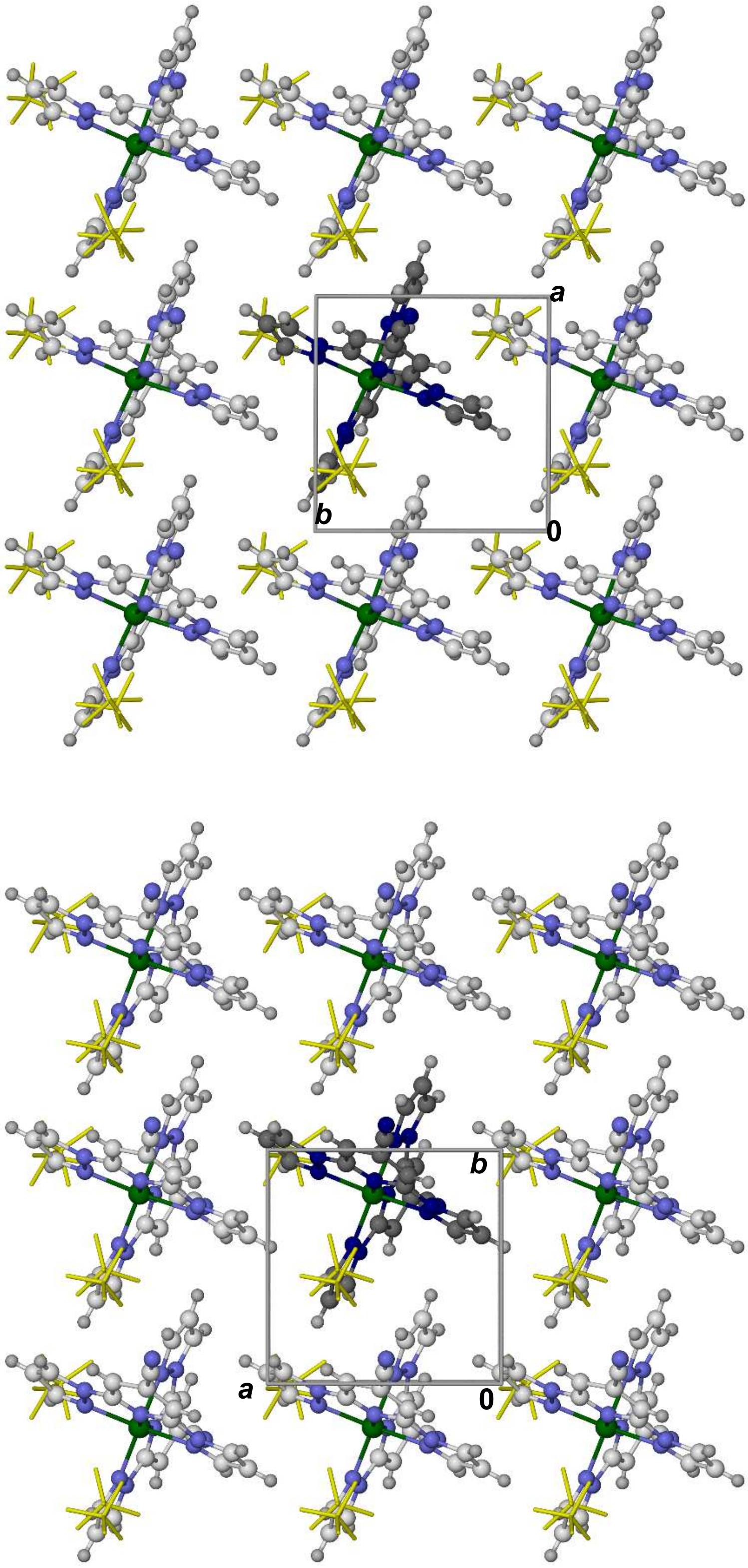

Figure S10 A terpyridine embrace cation layer in phases HS1 (top) and HS2 (bottom) of $[FeL_2][BF_4]_2$ at 120 K, viewed parallel to the [001] crystal vector. One environment for each cation is shown in dark coloration, while the anions are de-emphasized for clarity. All atoms have arbitrary radii.

Color code: C, white or dark gray; H, pale gray; Fe, green; N, pale or dark blue; BF_4^-, yellow.

Full packing diagrams for these structures are shown in Figure 2 of the main article.

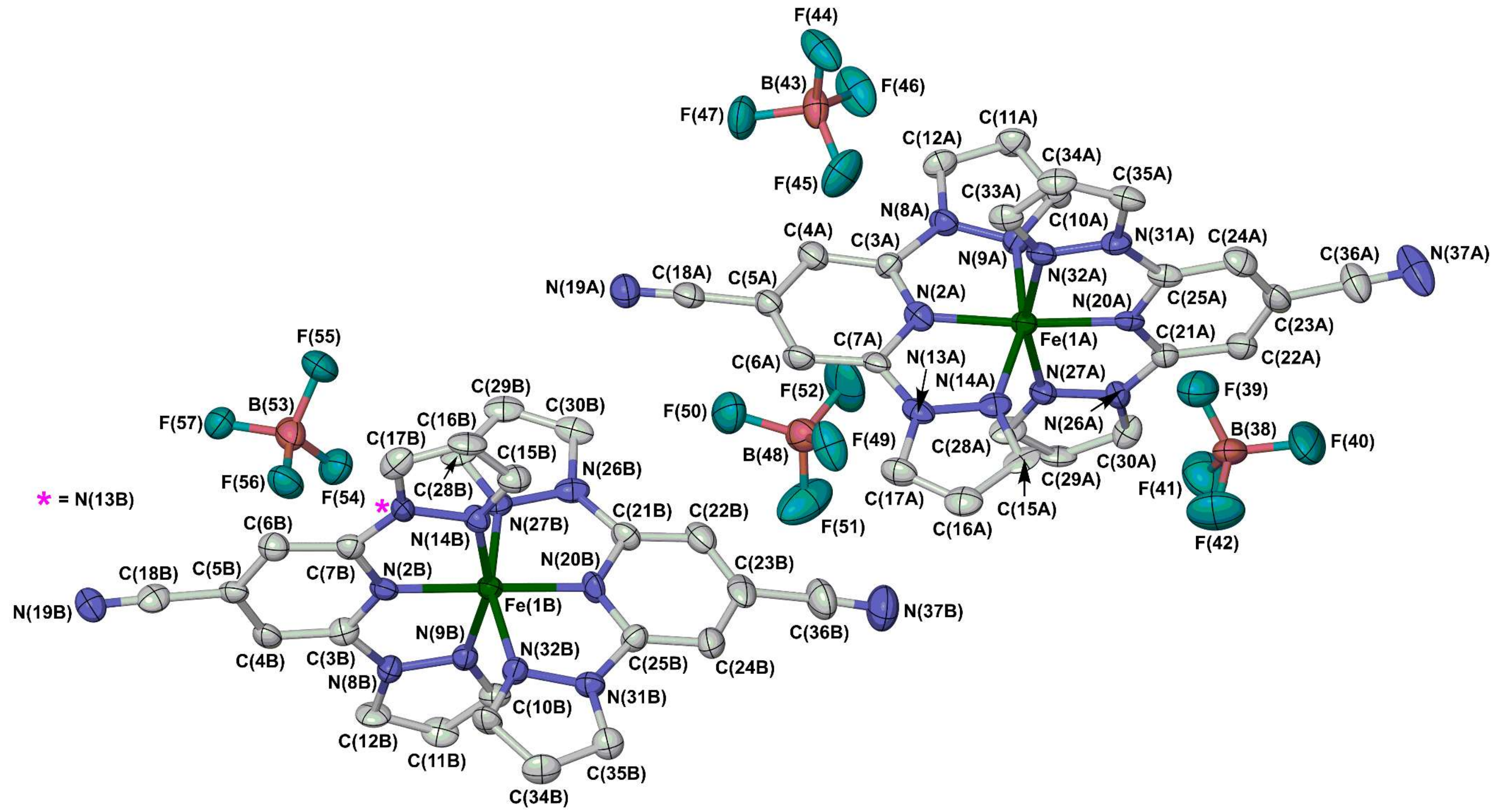

Figure S11 The asymmetric unit of $[FeL_2][BF_4]_2$ phase LS3 at 120 K, showing the full atom numbering scheme. Displacement ellipsoids are at the 50 % probability level, and H atoms are omitted for clarity.

Color code: C, white; B, pink; F, cyan; Fe, green; N, blue.

Table S3 Selected bond lengths [Å] and angles [deg] for $[FeL_2][BF_4]_2$ phase LS3 at 120 K. See Figure S11 for the atom numbering scheme, while the structural parameters at the foot of the Table are defined on page S9.

	Molecule A	Molecule B
Fe(1)–N(2)	1.896(6)	1.905(6)
Fe(1)–N(9)	1.969(7)	1.978(7)
Fe(1)–N(14)	1.993(7)	1.955(7)
Fe(1)–N(20)	1.891(6)	1.877(6)
Fe(1)–N(27)	1.959(6)	1.973(7)
Fe(1)–N(32)	1.977(7)	1.973(7)
N(2)–Fe(1)–N(9)	80.4(3)	80.3(3)
N(2)–Fe(1)–N(14)	79.8(3)	80.0(3)
N(2)–Fe(1)–N(20) (φ)	173.6(3)	178.6(3)
N(2)–Fe(1)–N(27)	99.6(3)	98.6(3)
N(2)–Fe(1)–N(32)	99.3(3)	99.9(3)
N(9)–Fe(1)–N(14)	160.2(3)	160.2(3)
N(9)–Fe(1)–N(20)	93.2(3)	98.4(3)
N(9)–Fe(1)–N(27)	89.8(3)	91.0(3)
N(9)–Fe(1)–N(32)	92.2(3)	91.5(3)
N(14)–Fe(1)–N(20)	106.6(3)	101.4(3)
N(14)–Fe(1)–N(27)	92.5(3)	91.6(3)
N(14)–Fe(1)–N(32)	92.0(3)	92.2(3)
N(20)–Fe(1)–N(27)	80.3(3)	81.0(3)
N(20)–Fe(1)–N(32)	80.8(3)	80.6(3)
N(27)–Fe(1)–N(32)	161.0(3)	161.5(3)
θ	87.73(6)	89.90(6)

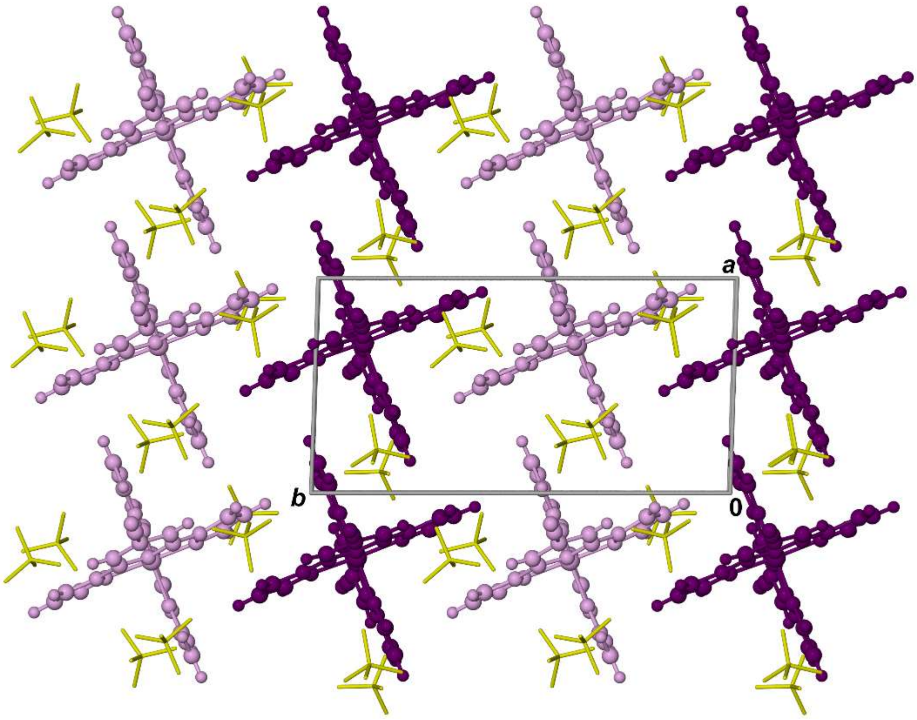

Figure S12 A terpyridine embrace cation layer in phase LS3 of $[FeL_2][BF_4]_2$ at 120 K, viewed perpendicular to the (001) crystal plane. The A and B cation sites are shown with pale and dark purple coloration, respectively, while the BF_4^- ions (yellow) are de-emphasized for clarity.

A full packing diagram for this structure is shown in Figure 4 (main article).

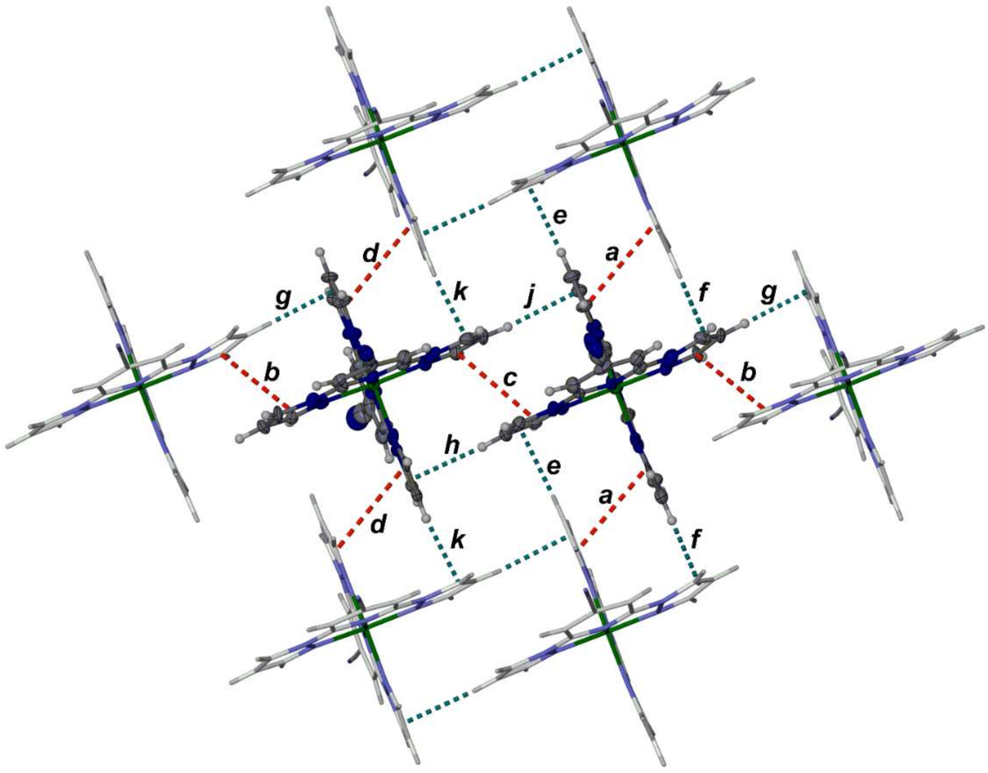

Figure S13 The intermolecular $\pi\cdots\pi$ (red) and C–H$\cdots\pi$ (cyan) contacts in a terpyridine embrace layer of phase LS3 at 120 K. The view is the same as in Figure S12. One A and one B cation is shown in dark coloration with 50 % displacement ellipsoids, while their neighbor molecules are de-emphasized for clarity. The letter labels for each interaction correspond to those in Table S4.

Color code: C, white or dark gray; H, pale gray; Fe, green; N, pale or dark blue.

Two pyrazolyl C–H groups in molecule B (on the left) have larger lateral offsets to their nearest neighbors in the layers, and do not form C–H$\cdots\pi$ contacts.

Table S4 Intermolecular interactions within the cation layers in the crystal lattice of phase LS3 at 120 K [Å, deg]. The letter labels correspond to those in Figure S13, while the atom numbering is shown in Figure S11. Each C–H$\cdots\pi$ contact is formed to the centroid of a pyrazolyl C–C bond.[a]

$\pi\cdots\pi$ interactions	Dihedral angle [°]	Interplanar distance [Å]	Horizontal offset [Å]
[N(8A)–C(12A)]$\cdots$[N(13A^{ii})–C(17A^{ii})] (***a***)	2.0(4)	3.49(2)	1.70
[N(26A)–C(30A)]$\cdots$[N(8B^{iii})–C(12B^{iii})] (***b***)	6.0(5)	3.21(3)	1.76
[N(31A)–C(35A)]$\cdots$[N(13B^{iv})–C(17B^{iv})] (***c***)	2.2(5)	3.45(3)	1.63
[N(26B)–C(30B)]$\cdots$[N(31B^{ii})–C(35B^{ii})] (***d***)	2.5(4)	3.35(3)	2.19

C–H$\cdots\pi$ interactions	C–H [Å]	H$\cdots$X [Å]	C$\cdots$X [Å]	C–H$\cdots$X [°]
C(11A)–H(11A)$\cdots$[C(33A^{ii}), C(34A^{ii})] (***e***)	0.95	2.65	3.59	169.2
C(16A)–H(16A)$\cdots$[C(28A^{v}), C(29A^{v})] (***f***)	0.95	2.79	3.72	168.7
C(29A)–H(29A)$\cdots$[C(33B^{iii}), C(34B^{iii})] (***g***)	0.95	2.61	3.55	171.0
C(34A)–H(34A)$\cdots$[C(28B^{iv}), C(29B^{iv})] (***h***)	0.95	2.77	3.71	173.1
C(16B)–H(16B)$\cdots$[C(10A^{iv}), C(11A^{iv})] (***j***)	0.95	2.78	3.69	161.6
C(29B)–H(29B)$\cdots$[C(16B^{ii}), C(17B^{ii})] (***k***)	0.95	2.79	3.72	168.4

[a]Symmetry codes: (ii) 1+*x*, *y*, *z*; (iii) 1–*x*, –*y*, 1–*z*; (iv) 1–*x*, 1–*y*, 1–*z*; (v) –1+*x*, *y*, *z*.

For comparison, the sum of the Pauling van der Waals radii of two aromatic rings is 3.4 Å, and for one H atom and one aromatic ring is 2.9 Å.[25]

Table S5 Variable temperature single crystal unit cell parameters for a crystal of the HS1 phase.

T [K]	300	250	200	150	250	275	300
Phase	HS1	HS1	LS3	LS3	HS1	HS1	HS1
Crystal system	Triclinic	Triclinic	Triclinic	Triclinic	Triclinic	Triclinic	Triclinic
Space group	$P\bar{1}$	$P\bar{1}$	$P\bar{1}$	$P\bar{1}$	$P\bar{1}$	$P\bar{1}$	$P\bar{1}$
Z	2	2	4	4	2	2	2
a [Å]	8.4864(13)	8.458(2)	8.556(3)	8.558(3)	8.453(2)	8.4716(16)	8.4820(17)
b [Å]	8.5043(12)	8.472(2)	16.865(6)	16.764(5)	8.464(2)	8.4826(15)	8.4911(15)
c [Å]	19.871(3)	19.800(5)	19.483(8)	19.465(9)	19.791(6)	19.832(4)	19.868(4)
α [deg]	81.785(11)	81.718(19)	95.44(3)	95.61(3)	81.61(2)	81.791(13)	81.805(15)
β [deg]	87.899(10)	87.815(18)	90.69(3)	90.58(2)	87.607(19)	87.794(12)	87.796(13)
γ [deg]	89.859(9)	89.897(18)	91.51(2)	91.49(2)	89.705(17)	89.814(12)	89.834(13)
V [Å^3]	1418.4(4)	1403.1(6)	2797.5(17)	2778.2(18)	1399.7(7)	1409.5(4)	1415.3(5)

Table S6 Variable temperature single crystal unit cell parameters for a crystal of the HS2 phase.

T [K]	300	250	200	150	250	275	300
Phase	HS2	HS2	LS4[a]	LS4[a]	LS4[a]	HS1	HS1
Crystal system	Tetragonal	Tetragonal	Monoclinic	Monoclinic	Monoclinic	Triclinic	Triclinic
Space group	$P4_3$	$P4_3$	$P2_1/n$	$P2_1/n$	$P2_1/n$	$P\bar{1}$	$P\bar{1}$
Z	4	4	4	4	4	2	2
a [Å]	8.4905(4)	8.4613(4)	8.468(6)	8.234(6)	8.452(2)	8.4747(9)	8.486(2)
b [Å]	8.4905(4)	8.4613(4)	38.70(2)	38.10(2)	38.522(12)	8.4802(9)	8.493(2)
c [Å]	39.283(3)	39.175(3)	8.559(6)	8.634(7)	8.461(3)	19.835(2)	19.869(5)
α [deg]	90	90	90	90	90	81.994(6)	81.893(15)
β [deg]	90	90	90.50(3)	94.65(5)	90.091(16)	87.882(7)	88.042(19)
γ [deg]	90	90	90	90	90	89.948(7)	89.977(17)
V [Å^3]	2831.9(3)	2804.7(3)	2805(3)	2700(3)	2755.0(15)	1410.6(3)	1416.9(7)

[a] While its approximate unit cell dimensions were clear, the crystal class and space group of the LS4 phase were not unambiguously identified from these data, or from the powder diffraction measurements. The monoclinic symmetry and space group of LS4 were defined by the synchrotron powder diffraction measurements, which were used as a basis for these unit cell refinements.

The larger canting of the unit cell β angle in LS4 at lower temperatures has precedent in some other compounds related to $[FeL_2][BF_4]_2$.[26,27]

Sample 1

300 K (inital – HS1 + HS2)

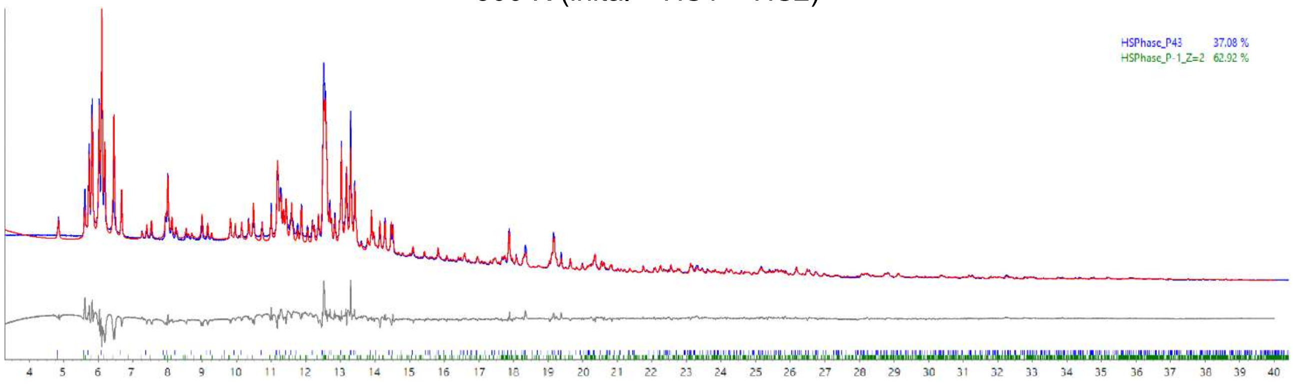

150 K (LS3 + LS4)

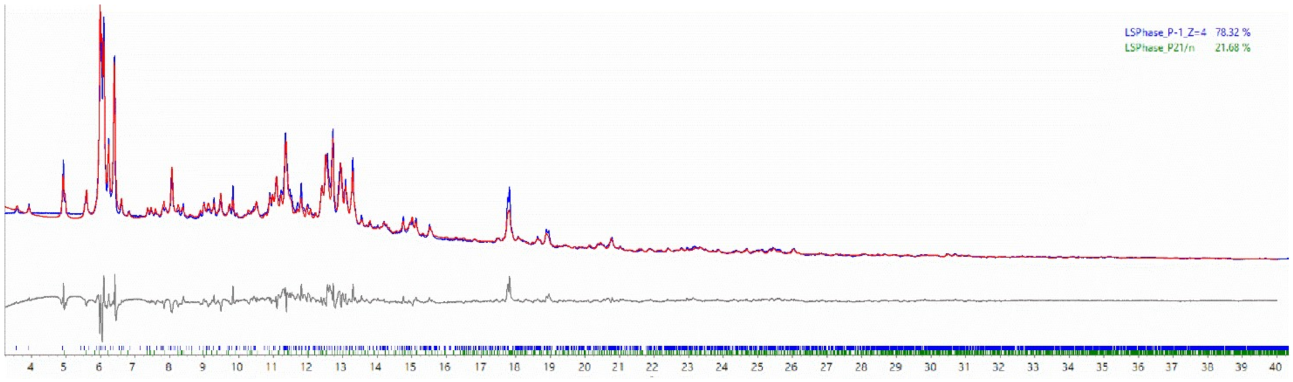

300 K (final – HS1 + HS2)

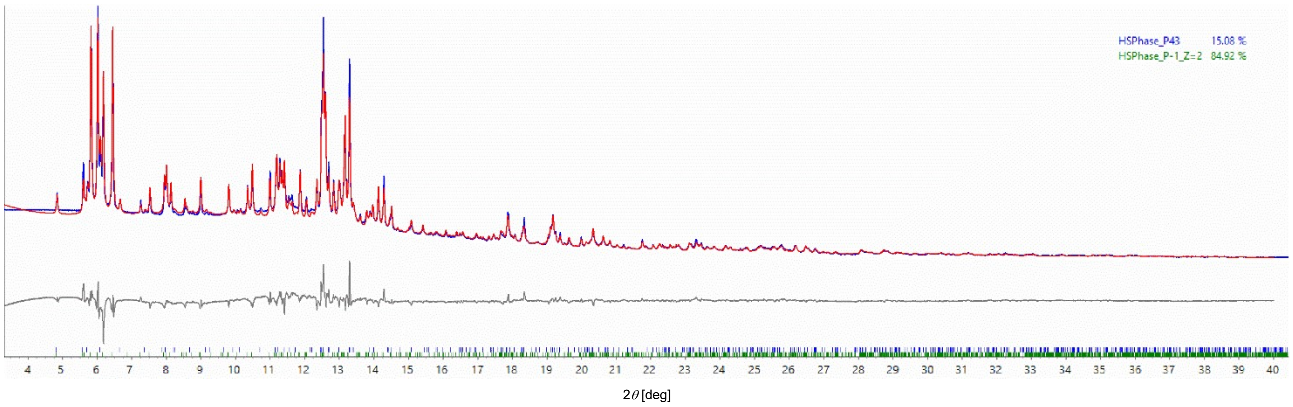

Figure S14 Rietveld refinement fits of $[FeL_2][BF_4]_2$ in its high-spin and low-spin states, from synchrotron powder diffraction data. Two different samples of the material were measured with a 300→150→300 K temperature cycle. Parameters from these fits are listed in Table S7.

Data from sample 2 are plotted on the next page.

Sample 2

300 K (inital – HS1 + HS2)

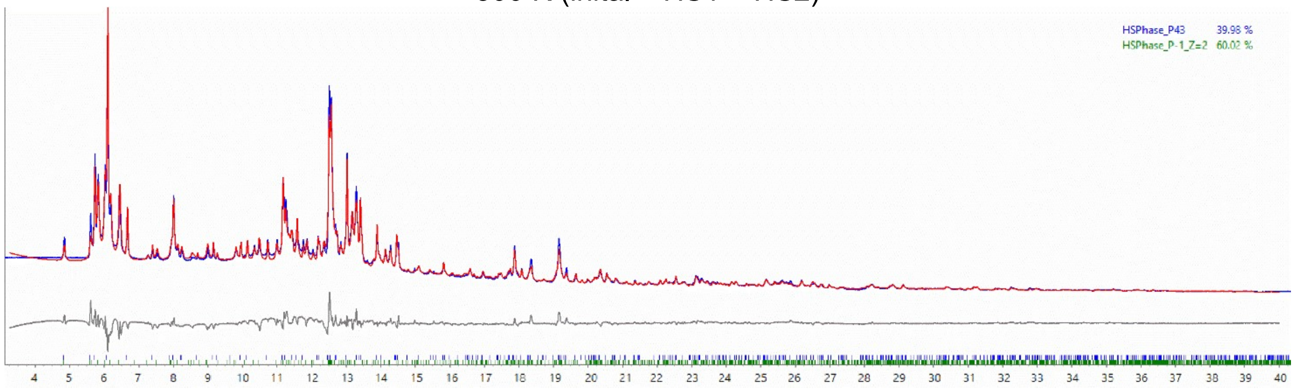

150 K (LS3 + LS4)

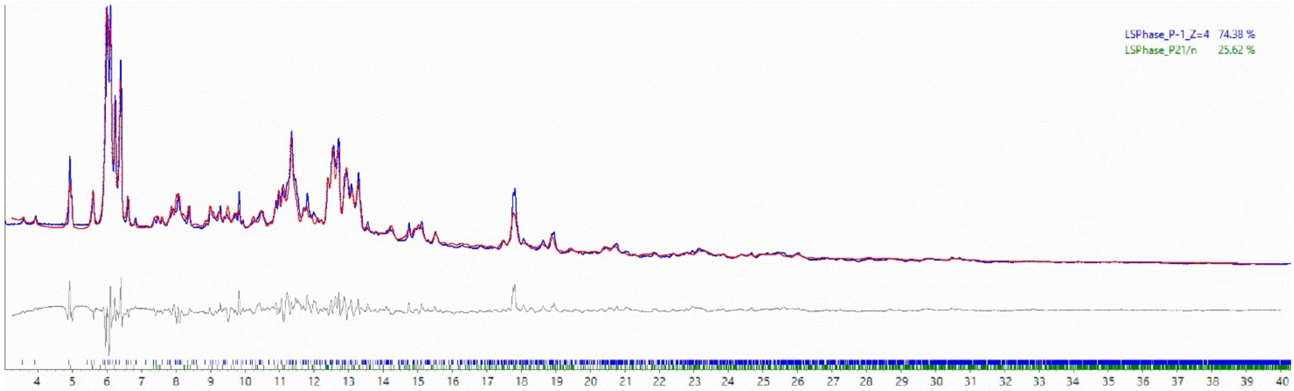

300 K (final – HS1 + HS2)

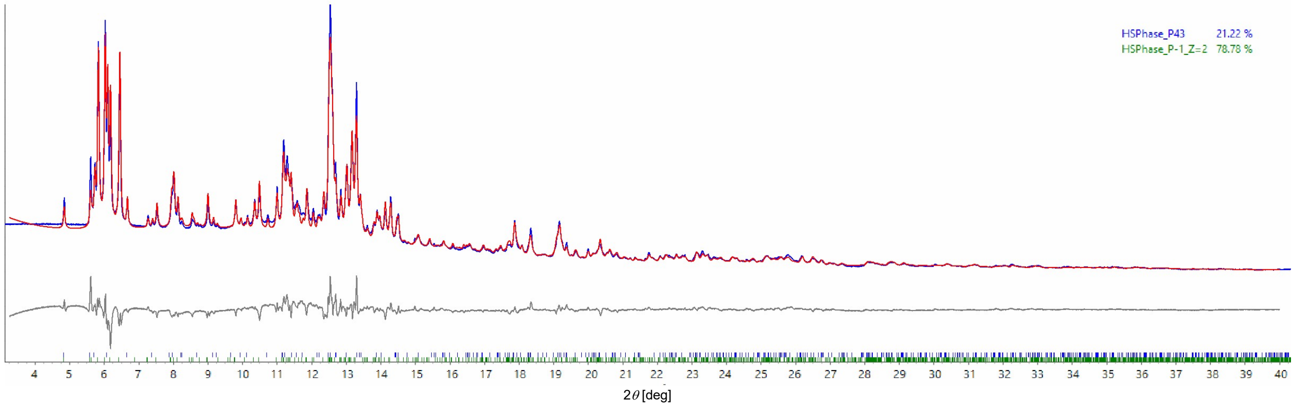

Figure S14 continued.

Table S7 Rietveld refinement results from the synchrotron X-ray powder diffraction data for $[FeL_2][BF_4]_2$ in its high-spin and low-spin forms (Figure S14). Two different samples of the material were measured with a 300→150→300 K temperature cycle. These parameters were used as a starting point for the fits of the Cu-K_α X-ray powder diffraction data in Tables S8-S10.

													Parameters						
T [K]	Phase	Space Group	V [Å^3]	a [Å]	b [Å]	c [Å]	α [deg]	β [deg]	γ [deg]	%	R_{wp}	$R_{wp'}$	Total	Back-ground	Lattice	Displace-ment	Peak Shapes	Scale	Zero Point
Sample 1																			
300	HS1	$P\bar{1}$	1412.49(8)	8.4768(3)	8.4935(3)	19.8318(7)	81.858(3)	87.881(3)	89.821(3)	62.9(2)	0.0462	0.1898	29	9	8	0	9	2	1
	HS2	$P4_3$	2820.4(2)	8.4791(2)	8.4791(2)	39.229(1)	90	90	90	37.1(2)									
150	LS3	$P\bar{1}$	2765.2(4)	8.5529(7)	16.758(1)	19.381(2)	95.223(4)	90.628(4)	91.525(4)	78.3(5)	0.0492	0.1993	49	9	10	18	9	2	1
	LS4	$P2_1/n$	2767.1(7)	8.3392(9)	38.162(8)	8.741(1)	90	95.87(1)	90	21.7(5)									
300	HS1	$P\bar{1}$	1412.10(8)	8.4805(3)	8.4875(3)	19.8304(7)	81.882(2)	87.899(2)	89.697(2)	84.9(3)	0.0427	0.1745	29	9	8	0	9	2	1
	HS2	$P4_3$	2821.5(6)	8.4814(5)	8.4814(5)	39.223(6)	90	90	90	15.1(3)									
Sample 2																			
300	HS1	$P\bar{1}$	1412.1(1)	8.4756(4)	8.4889(4)	19.836(1)	81.952(5)	87.919(5)	89.876(5)	60.0(2)	0.0464	0.1670	29	9	8	0	9	2	1
	HS2	$P4_3$	2821.2(2)	8.4794(2)	8.4794(2)	39.238(2)	90	90	90	40.0(2)									
150	LS3	$P\bar{1}$	2776.0(5)	8.5388(7)	16.826(1)	19.409(3)	95.287(7)	90.477(8)	91.064(6)	74.4(5)	0.0581	0.1943	49	9	10	18	9	2	1
	LS4	$P2_1/n$	2780.2(7)	8.3557(9)	38.239(7)	8.7440(9)	90	95.67(1)	90	25.6(5)									
300	HS1	$P\bar{1}$	1411.5(2)	8.4776(6)	8.4837(5)	19.834(1)	81.950(3)	87.973(3)	89.761(3)	78.8(3)	0.0456	0.1609	29	9	8	0	9	2	1
	HS2	$P4_3$	2821.0(5)	8.4822(6)	8.4822(6)	39.210(4)	90	90	90	21.2(3)									

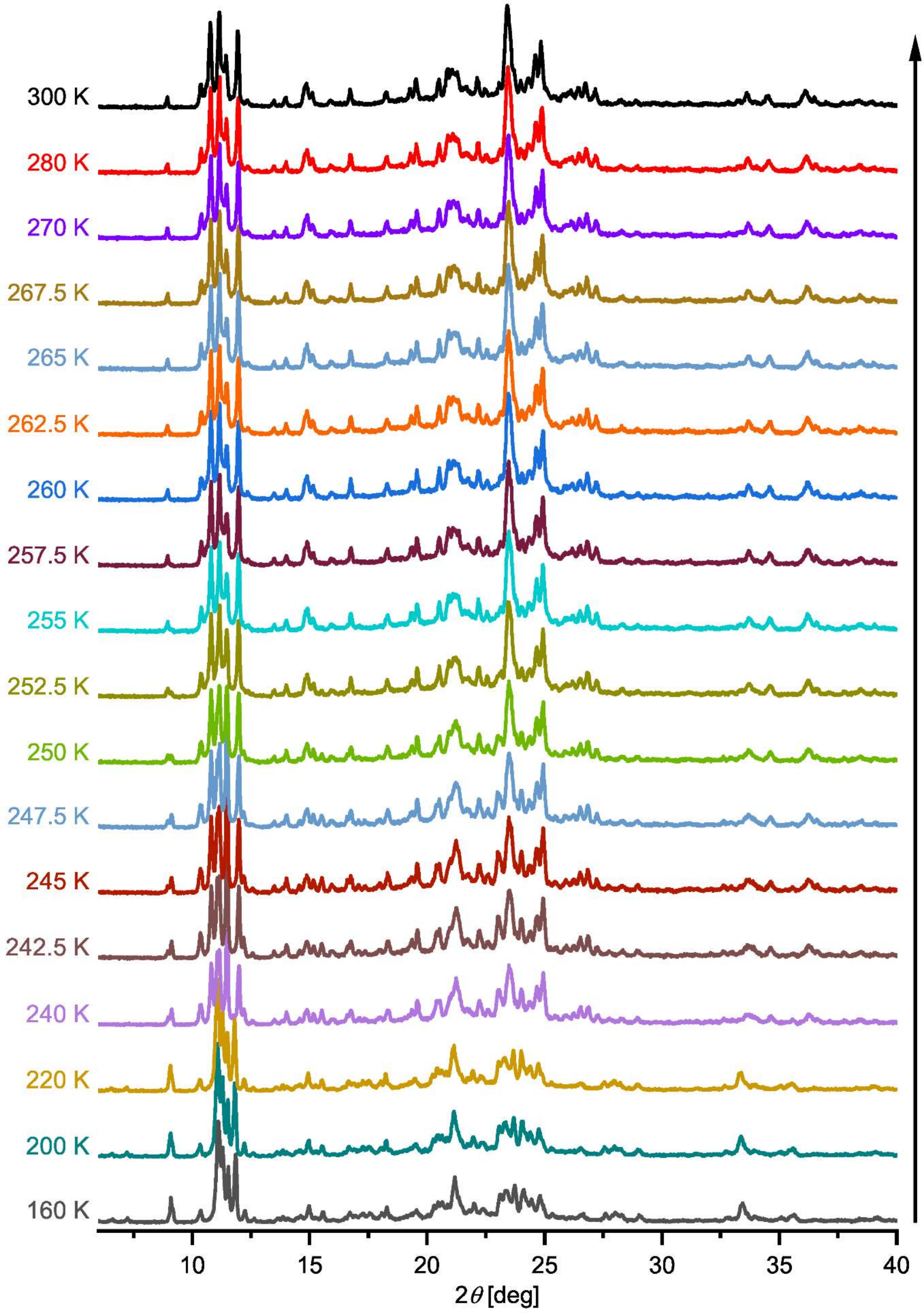

Figure S15 Variable temperature powder X-ray diffraction data from $[FeL_2][BF_4]_2$, measured with Cu-K_α radiation in warming mode. The plot contains data from Figure 5 (main article), with additional temperatures included.

Unlike the cooling mode data, where the high→low-spin transition occurs abruptly between 240 and 220 K (Figure 5), the high→low-spin transition powder pattern evolves back to its original form between 240 and 260 K on warming.

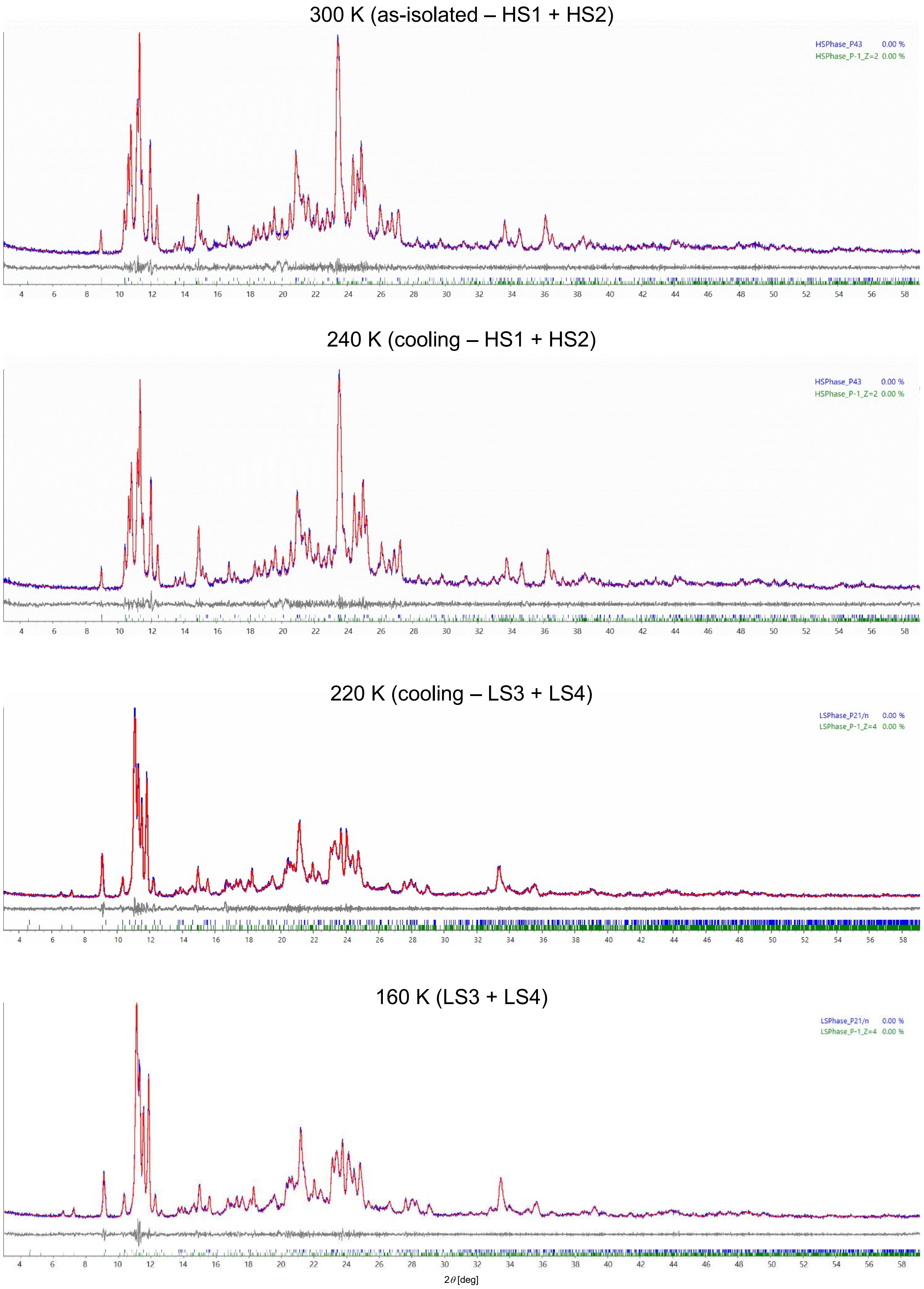

Figure S16 Representative Pawley refinement fits of powder diffraction data from Figures 5 (main article) and S15. Measured data are plotted in dark blue, the refinement fits are in red and the difference plot is gray.

Unit cell parameters derived from these fits are listed in Table S8.

220 K (warming – LS3 + LS4)

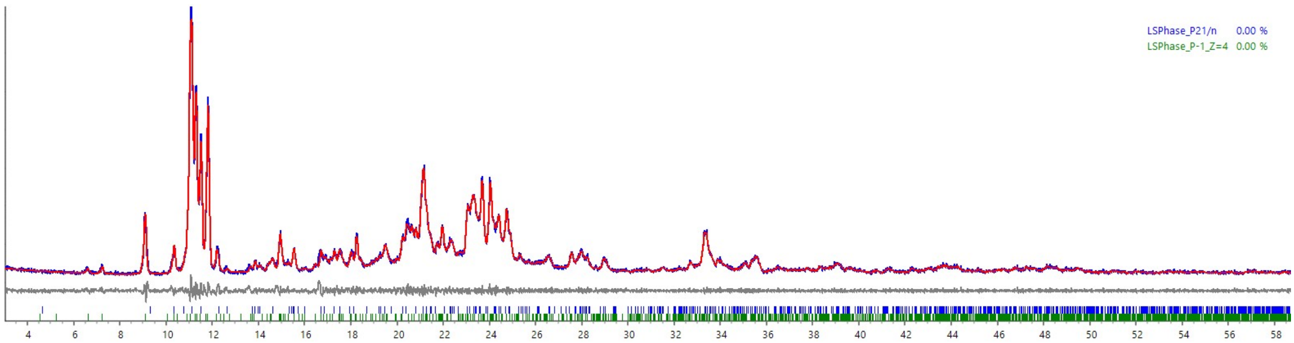

235 K (warming – HS1 + LS3 + LS4)

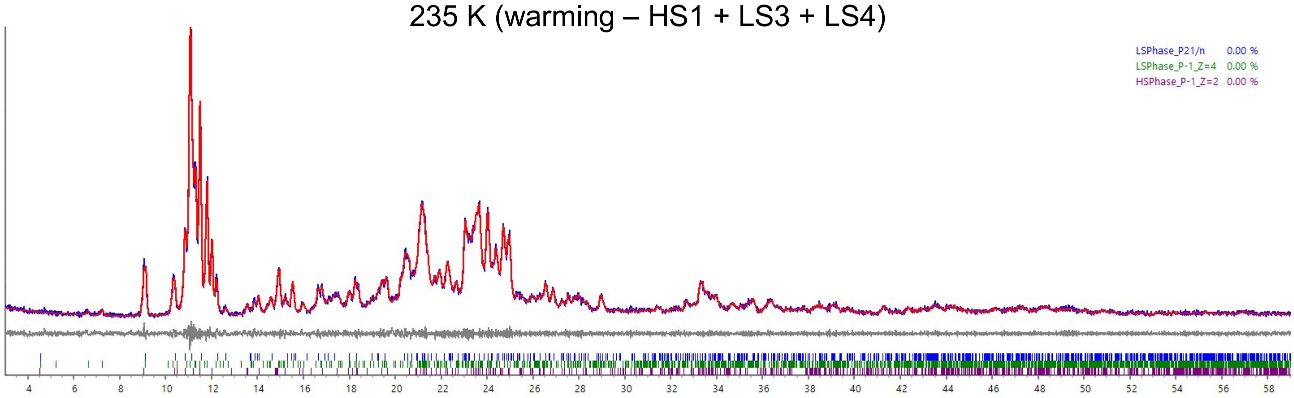

240 K (warming – HS1 + LS3 + LS4)

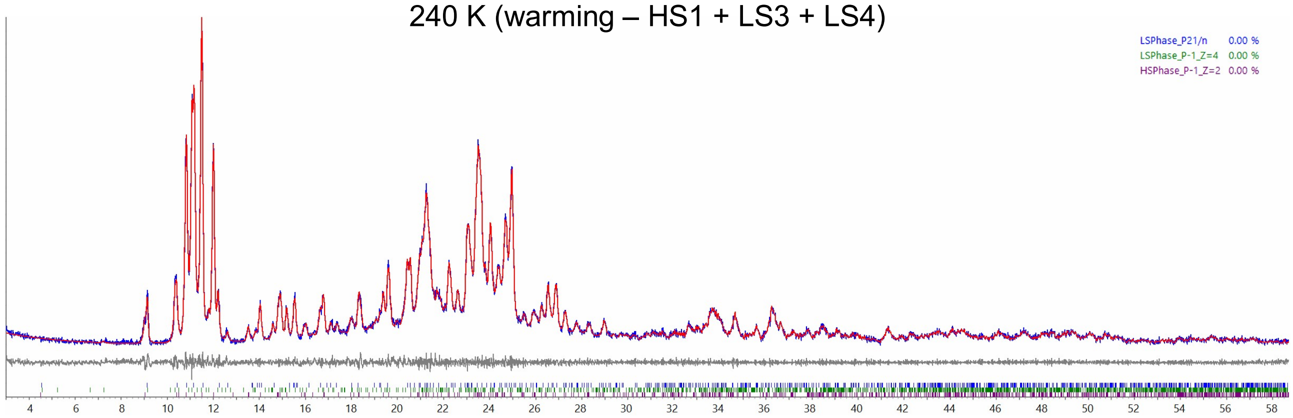

245 K (warming – HS1 + LS3 + LS4)

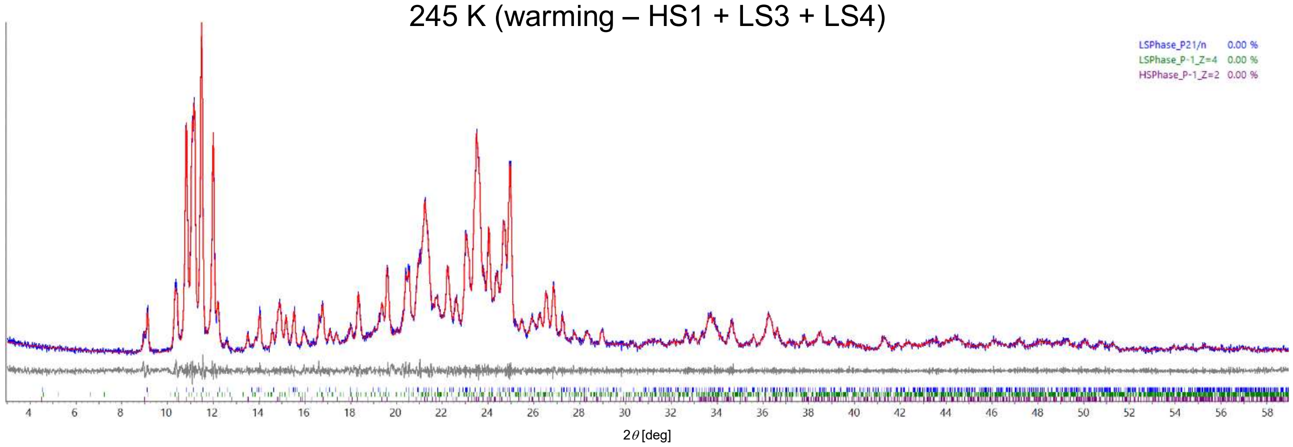

Figure S16 continued.

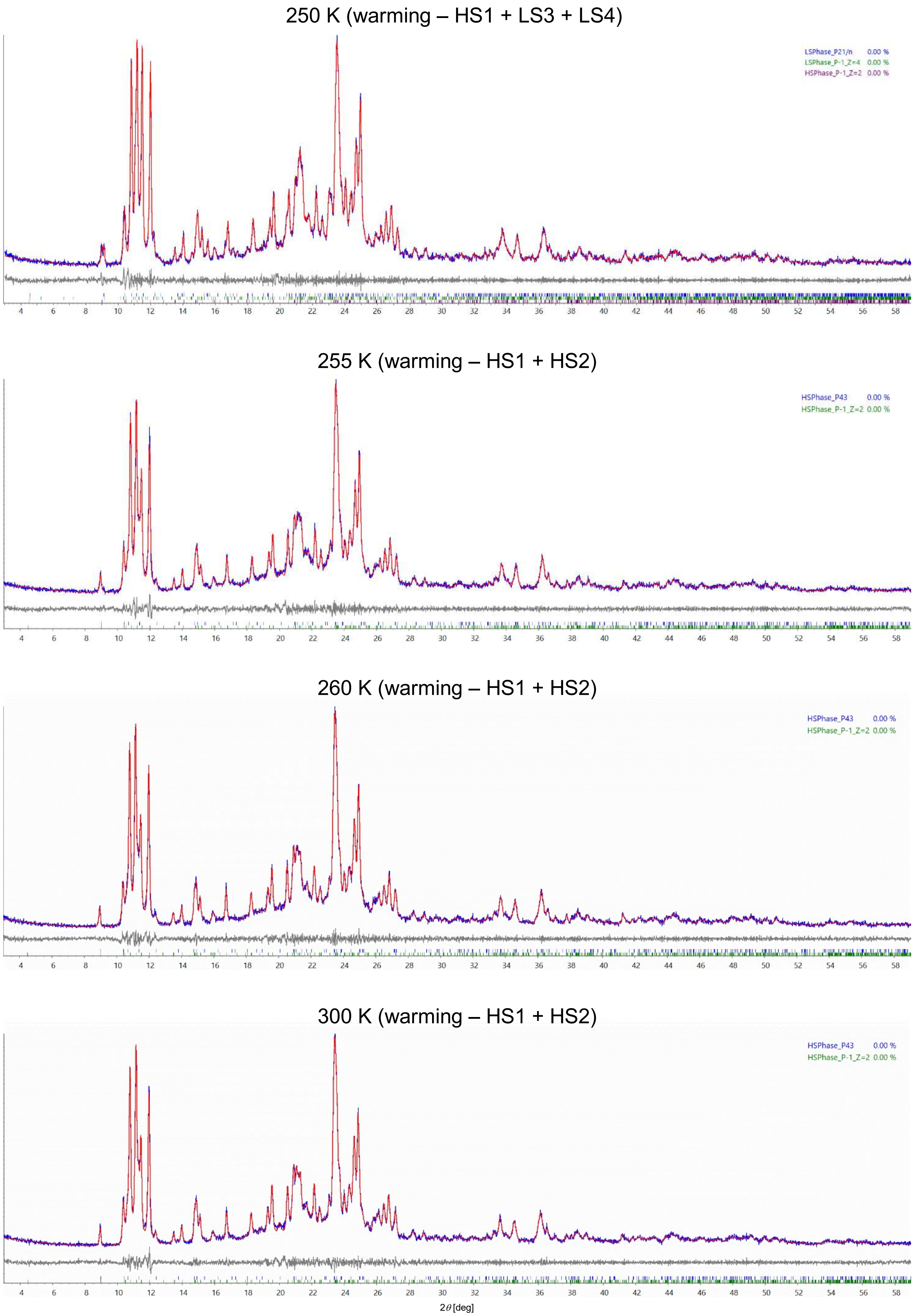

Figure S16 continued.

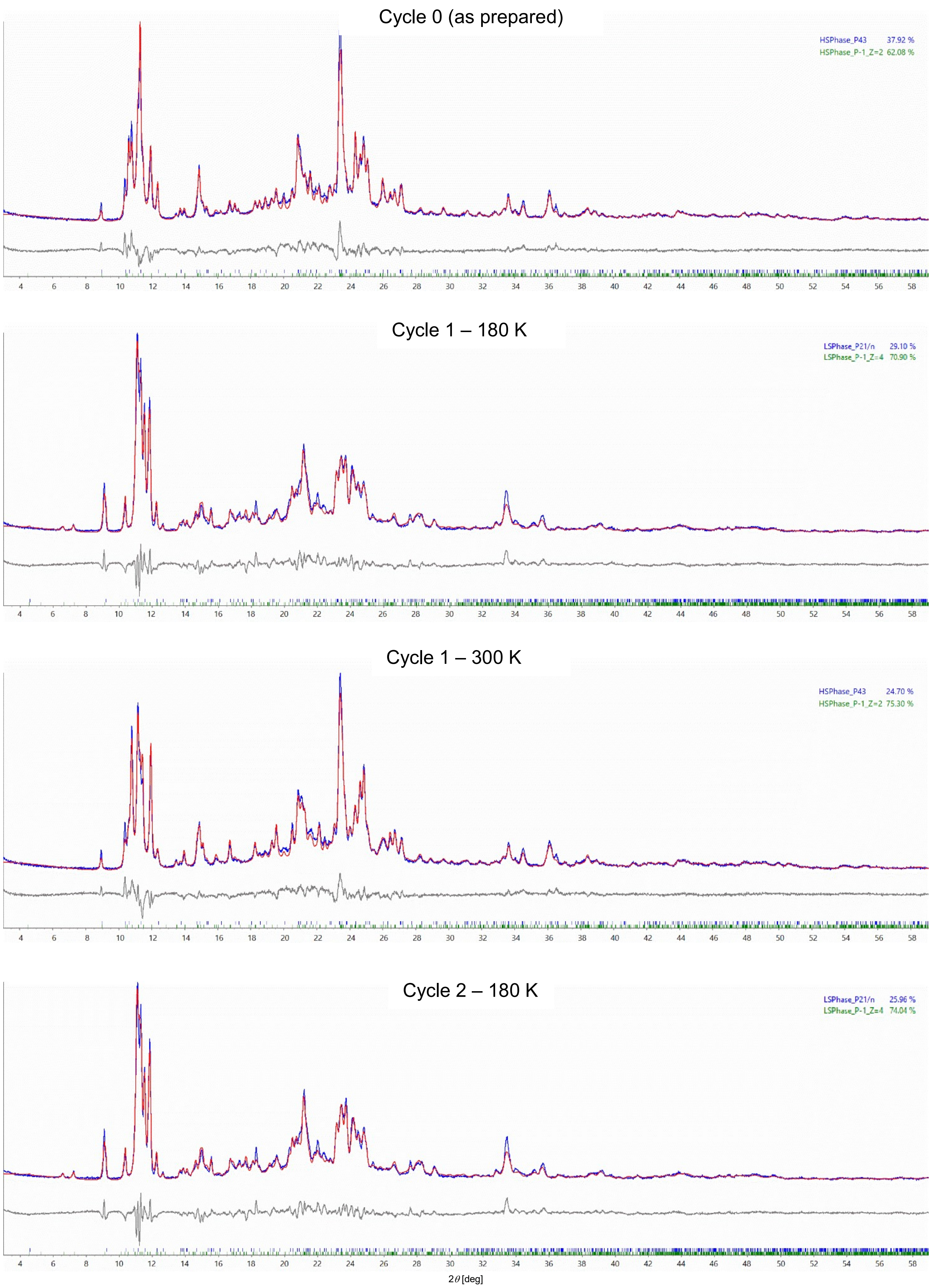

Figure S17 Measured data (blue), Rietveld refinement fits (red) and difference plots (gray) giving the phase composition of high-spin and low-spin $[FeL_2][BF_4]_2$ in successive thermal cycles, and after thermal annealing (Table S10). The sample was cycled about four 300→180→300 K temperature cycles, then annealed at 398 K for 24 hrs.

Cycle 2 – 300 K

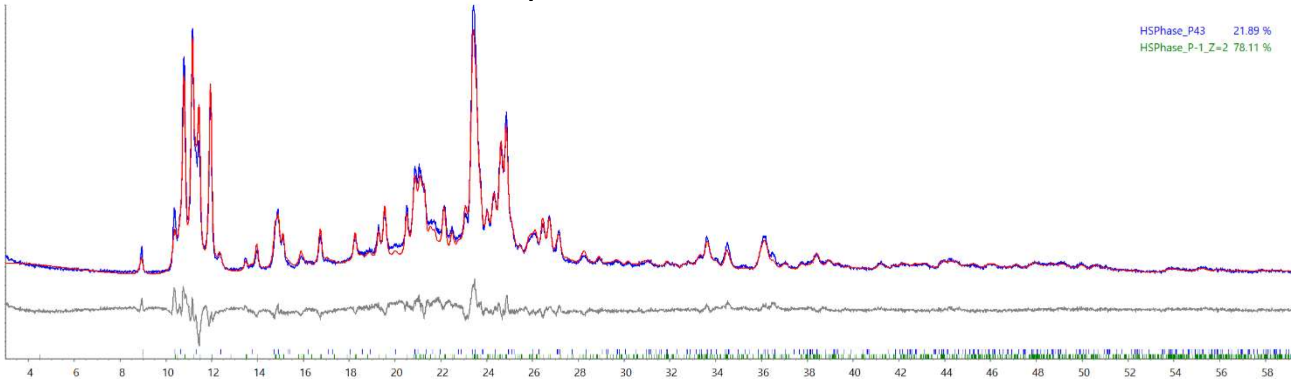

Cycle 3 – 180 K

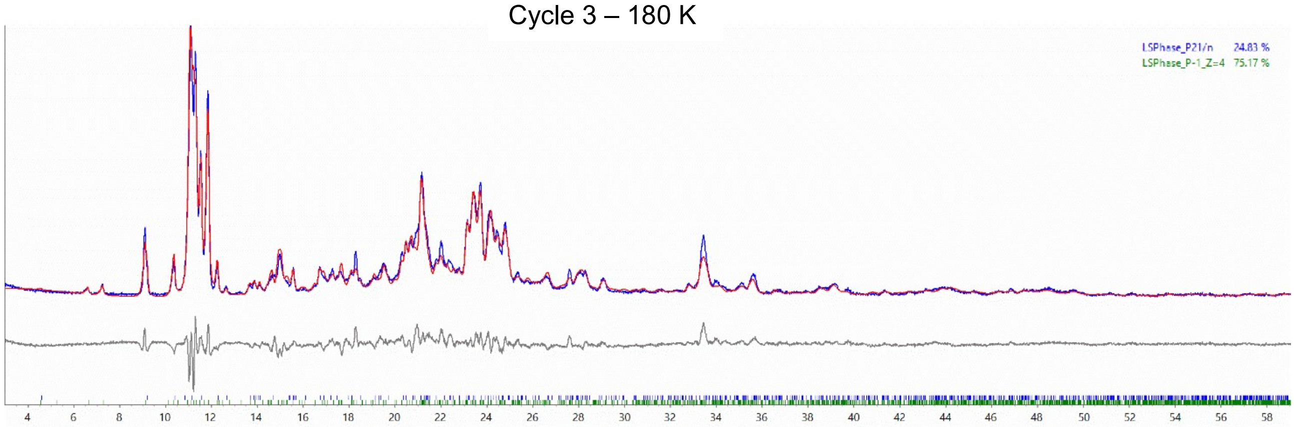

Cycle 3 – 300 K

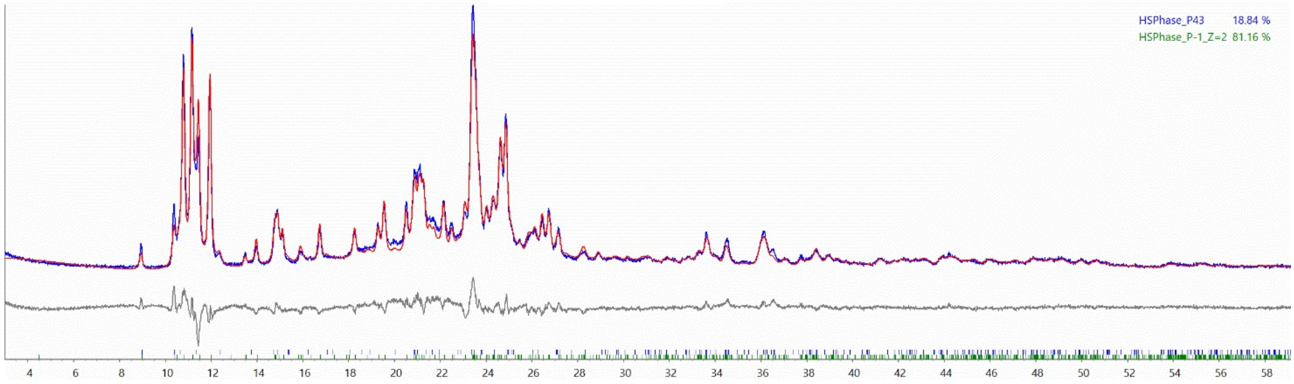

Cycle 4 – 180 K

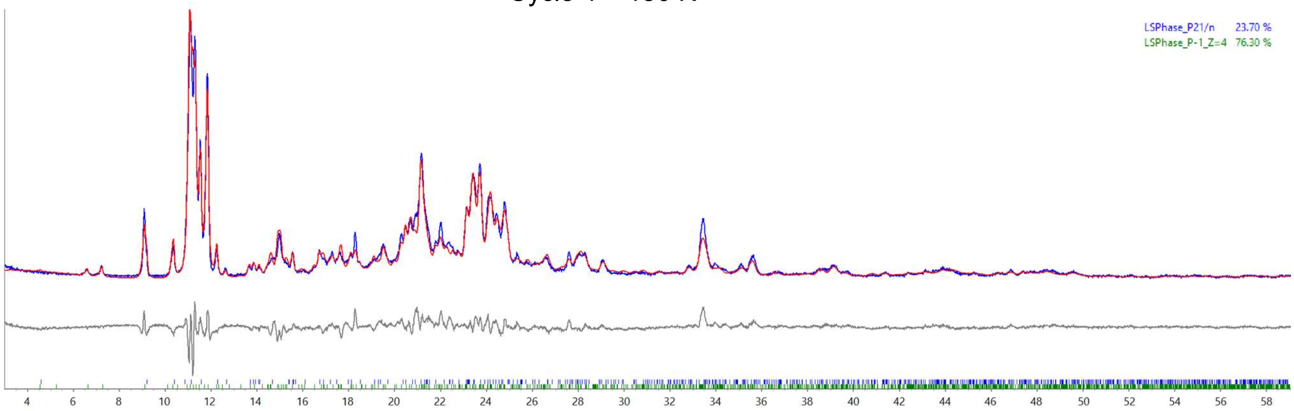

Figure S17 continued.

Cycle 4 – 300 K

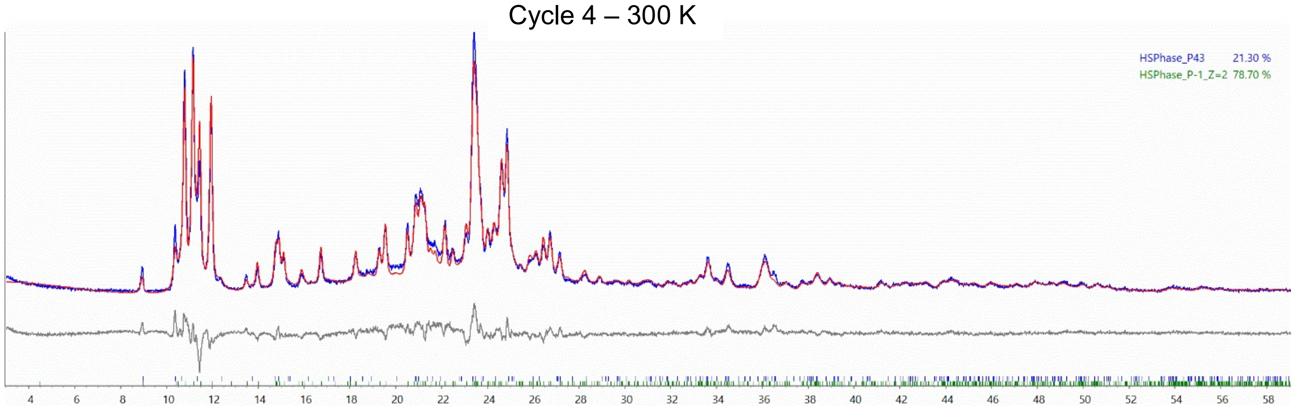

Before annealing

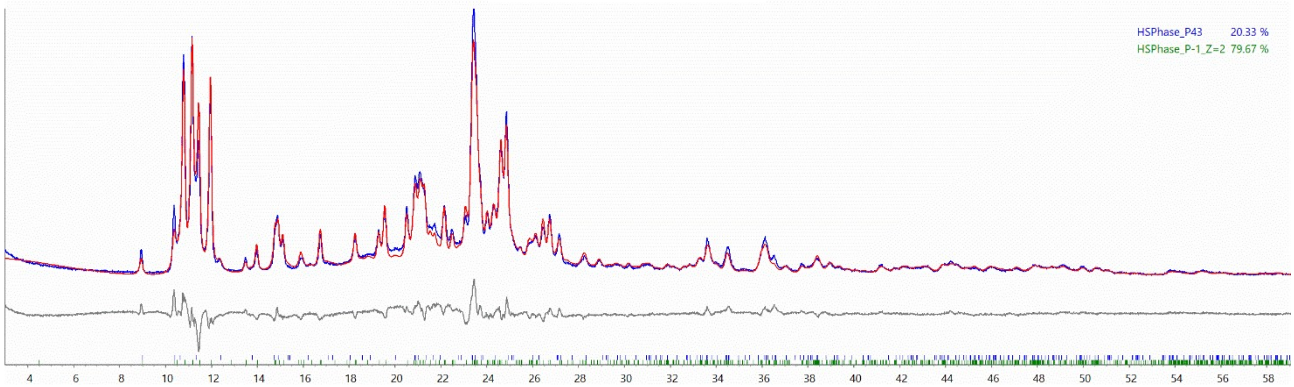

After annealing

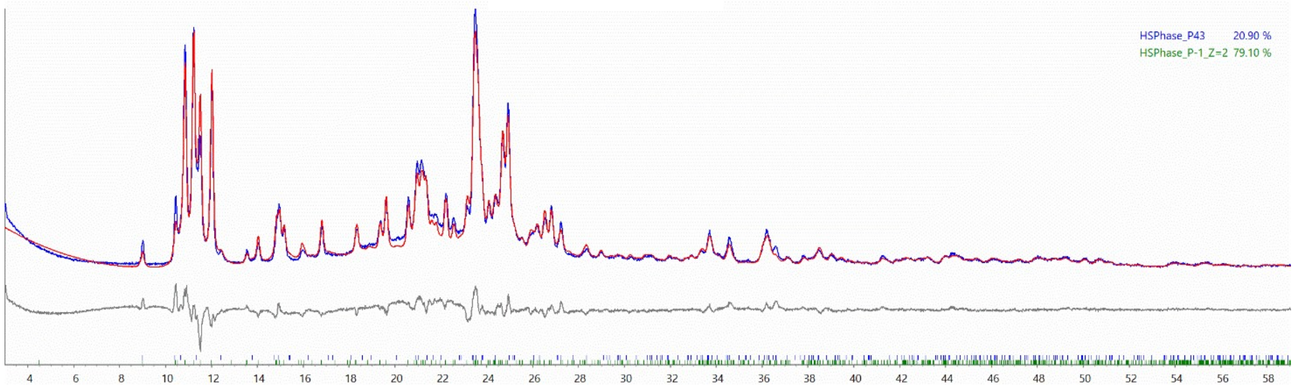

Figure S17 continued.

Table S8 Pawley phase fits of the variable temperature Cu-K_α X-ray powder diffraction data for $[FeL_2][BF_4]_2$ (Figures 5 and S16) − full thermal cycle.

T [K]	Phase	Space Group	V [Å^3]	a [Å]	b [Å]	c [Å]	α [deg]	β [deg]	γ [deg]	R_{wp}	$R_{wp'}$	Total Parameters	Parameters Excl Peaks	Background Parameters	Lattice Parameters	Peak Shapes	Zero Point
300	HS1	$P\bar{1}$	1420.2(2)	8.4903(5)	8.5082(5)	19.872(1)	81.881(4)	87.916(4)	89.82(4)	0.0686	0.1061	1297	27	9	8	9	1
	HS2	$P4_3$	2834.1(3)	8.4917(4)	8.4917(4)	39.304(2)	90	90	90								
280	HS1	$P\bar{1}$	1415.9(1)	8.4817(4)	8.4981(5)	19.856(1)	81.890(4)	87.873(4)	89.82(5)	0.0673	0.0985	1298	27	9	8	9	1
	HS2	$P4_3$	2826.2(3)	8.4822(4)	8.4822(4)	39.282(3)	90	90	90								
260	HS1	$P\bar{1}$	1412.5(1)	8.4777(4)	8.4926(4)	19.832(1)	81.873(4)	87.799(4)	89.83(4)	0.0687	0.0978	1297	27	9	8	9	1
	HS2	$P4_3$	2821.0(3)	8.4759(4)	8.4759(4)	39.268(2)	90	90	90								
240	HS1	$P\bar{1}$	1405.5(2)	8.4605(5)	8.4763(5)	19.812(1)	81.902(4)	87.768(4)	89.80(5)	0.0681	0.1004	1290	27	9	8	9	1
	HS2	$P4_3$	2805.8(3)	8.4618(4)	8.4618(4)	39.186(2)	90	90	90								
220	LS3	$P\bar{1}$	2810.6(2)	8.5551(4)	16.9196(7)	19.5055(9)	95.264(3)	90.290(3)	91.400(3)	0.0642	0.0856	2493	29	9	10	9	1
	LS4	$P2_1/n$	2832.3(4)	8.4153(6)	38.566(4)	8.7718(5)	90	95.799(6)	90								
200	LS3	$P\bar{1}$	2809.9(2)	8.5611(3)	16.9059(7)	19.5026(8)	95.265(2)	90.311(2)	91.353(3)	0.0623	0.0717	2489	29	9	10	9	1
	LS4	$P2_1/n$	2828.7(3)	8.4098(5)	38.556(3)	8.7693(5)	90	95.839(5)	90								
180	LS3	$P\bar{1}$	2816.9(2)	8.5711(3)	16.9169(7)	19.5160(9)	95.290(3)	90.327(3)	91.330(3)	0.0627	0.0826	2498	29	9	10	9	1
	LS4	$P2_1/n$	2830.3(4)	8.4189(5)	38.503(3)	8.7773(5)	90	95.865(7)	90								
160	LS3	$P\bar{1}$	2800.1(2)	8.5579(3)	16.8772(6)	19.4774(8)	95.326(2)	90.384(3)	91.410(3)	0.0616	0.0803	2485	29	9	10	9	1
	LS4	$P2_1/n$	2811.8(3)	8.3973(5)	38.399(3)	8.7658(5)	90	95.852(5)	90								
200	LS3	$P\bar{1}$	2812.0(2)	8.5590(4)	16.9158(7)	19.5084(9)	95.226(2)	90.261(3)	91.271(3)	0.0654	0.0769	2493	29	9	10	9	1
	LS4	$P2_1/n$	2811.0(4)	8.4381(6)	38.109(3)	8.7871(6)	90	95.845(7)	90								
210	LS3	$P\bar{1}$	2812.4(2)	8.5563(4)	16.9254(8)	19.5080(9)	95.255(3)	90.306(3)	91.340(3)	0.0656	0.0907	2492	29	9	10	9	1
	LS4	$P2_1/n$	2825.0(4)	8.4284(5)	38.368(4)	8.7818(6)	90	95.872(7)	90								
220	LS3	$P\bar{1}$	2816.6(2)	8.5533(4)	16.9414(7)	19.5248(9)	95.250(3)	90.150(3)	91.343(3)	0.0673	0.0808	2497	29	9	10	9	1
	LS4	$P2_1/n$	2806.0(3)	8.4392(5)	37.961(3)	8.8025(5)	90	95.709(6)	90								
230	LS3	$P\bar{1}$	2816.9(2)	8.5510(4)	16.9400(8)	19.5320(9)	95.193(3)	90.123(3)	91.306(3)	0.0700	0.0871	2500	29	9	10	9	1
	LS4	$P2_1/n$	2784.2(3)	8.3709(5)	37.963(2)	8.8021(5)	90	95.518(5)	90								
235	HS1	$P\bar{1}$	1411.5(1)	8.5004(4)	8.4986(3)	19.7536(8)	81.851(3)	87.777(3)	89.407(3)	0.0583	0.0646	3337	39	9	16	13	1
	LS3	$P\bar{1}$	2802.8(2)	8.5629(3)	16.9170(7)	19.4273(8)	95.012(3)	90.126(2)	91.262(3)								
	LS4	$P2_1/n$	2823.7(5)	8.386(1)	38.745(4)	8.7414(7)	90	96.16(1)	90								

Table S8 continued.

T [K]	Phase	Space Group	V [Å^3]	a [Å]	b [Å]	c [Å]	α [deg]	β [deg]	γ [deg]	R_{wp}	$R_{wp'}$	Total Parameters	Parameters Excl Peaks	Background Parameters	Lattice Parameters	Peak Shapes	Zero Point
	HS1	$P\bar{1}$	1416.06(9)	8.5019(3)	8.5034(3)	19.8041(8)	81.815(3)	87.744(3)	89.434(3)								
240	LS3	$P\bar{1}$	2800.4(1)	8.5587(2)	16.9190(5)	19.4174(5)	94.997(2)	90.027(2)	91.249(2)	0.0589	0.0565	3352	39	9	16	13	1
	LS4	$P2_1/n$	2824.2(2)	8.3849(3)	38.766(1)	8.7388(3)	90	96.142(2)	90								
	HS1	$P\bar{1}$	1421.2(1)	8.4993(3)	8.5154(4)	19.8560(8)	81.811(2)	87.676(3)	89.477(3)								
245	LS3	$P\bar{1}$	2805.6(2)	8.5623(3)	16.9392(5)	19.4229(6)	95.010(2)	90.003(2)	91.301(2)	0.0602	0.673	3355	39	9	16	13	1
	LS4	$P2_1/n$	2829.5(2)	8.3878(3)	38.797(1)	8.7449(3)	90	96.136(3)	90								
	HS1	$P\bar{1}$	1420.1(1)	8.4977(4)	8.5157(4)	19.8441(8)	81.782(4)	87.705(3)	89.433(3)								
250	LS3	$P\bar{1}$	2804.9(2)	8.5625(3)	16.9393(7)	19.4173(7)	94.990(3)	90.020(3)	91.312(3)	0.0613	0.0796	3357	39	9	16	13	1
	LS4	$P2_1/n$	2827.2(2)	8.3842(4)	38.794(2)	8.7419(4)	90	96.116(4)	90								
255	HS1	$P\bar{1}$	1408.2(1)	8.4716(5)	8.4739(5)	19.826(1)	81.945(3)	87.815(3)	89.713(3)	0.0672	0.0823	1290	27	9	8	9	1
	HS2	$P4_3$	2804.2(5)	8.4657(6)	8.4657(6)	39.127(4)	90	90	90								
260	HS1	$P\bar{1}$	1411.2(1)	8.4767(4)	8.4818(4)	19.838(1)	81.939(3)	87.805(3)	89.730(3)	0.0647	0.0825	1297	27	9	8	9	1
	HS2	$P4_3$	2813.5(6)	8.4715(8)	8.4715(8)	39.203(5)	90	90	90								
265	HS1	$P\bar{1}$	1411.5(1)	8.4764(4)	8.4829(5)	19.840(1)	81.941(3)	87.851(3)	89.727(3)	0.0662	0.0817	1297	27	9	8	9	1
	HS2	$P4_3$	2815.3(5)	8.4712(6)	8.4712(6)	39.232(4)	90	90	90								
270	HS1	$P\bar{1}$	8.4804(4)	8.4865(5)	19.844(1)	81.930(4)	87.838(4)	89.726(3)	8.4804(4)	0.0663	0.0973	1297	27	9	8	9	1
	HS2	$P4_3$	8.472(1)	8.472(1)	39.27(1)	90	90	90	8.472(1)								
275	HS1	$P\bar{1}$	1413.9(1)	8.4810(5)	8.4891(5)	19.849(1)	81.927(3)	87.873(3)	89.724(4)	0.0668	0.0785	1297	27	9	8	9	1
	HS2	$P4_3$	2821.6(4)	8.4755(5)	8.4755(5)	39.279(3)	90	90	90								
280	HS1	$P\bar{1}$	1414.2(1)	8.4810(5)	8.4903(5)	19.850(1)	81.930(3)	87.887(3)	89.762(4)	0.0666	0.0880	1297	27	9	8	9	1
	HS2	$P4_3$	2821.4(5)	8.4754(5)	8.4754(5)	39.278(4)	90	90	90								
290	HS1	$P\bar{1}$	1417.2(1)	8.4866(5)	8.4975(5)	19.862(1)	81.927(3)	87.903(3)	89.752(4)	0.0677	0.0866	1298	27	9	8	9	1
	HS2	$P4_3$	2826.3(4)	8.4811(4)	8.4811(4)	39.292(4)	90	90	90								
300	HS1	$P\bar{1}$	1419.5(1)	8.4908(5)	8.5031(5)	19.871(1)	81.929(3)	87.926(4)	89.781(4)	0.0671	0.1014	1297	27	9	8	9	1
	HS2	$P4_3$	2830.8(6)	8.4870(8)	8.4870(8)	39.302(5)	90	90	90								

Table S9 Pawley phase fits of the variable temperature the Cu-K_α X-ray powder diffraction data for $[FeL_2][BF_4]_2$ − multiple thermal cycles.

T [K]	Phase	Space Group	*V* [Å^3]	*a* [Å]	*b* [Å]	*c* [Å]	*α* [deg]	*β* [deg]	*γ* [deg]	R_{wp}	$R_{wp'}$	Total Parameters	Parameters Excl Peaks	Background Parameters	Lattice Parameters	Peak Shapes	Zero Point
300	HS1	$P\bar{1}$	1423.3(2)	8.4951(6)	8.5166(5)	19.880(1)	81.965(3)	87.951(3)	89.955(3)	0.0491	0.0624	1299	27	9	8	9	1
	HS2	$P4_3$	2844.5(4)	8.4987(6)	8.4987(6)	39.382(3)	90	90	90								
Cycle 1																	
250	HS1	$P\bar{1}$	1413.2(2)	8.4756(6)	8.4890(7)	19.850(2)	81.989(4)	87.770(4)	89.944(5)	0.0682	0.0920	1297	27	9	8	9	1
	HS2	$P4_3$	2822.1(5)	8.4774(6)	8.4774(6)	39.268(4)	90	90	90								
180	LS3	$P\bar{1}$	2806.4(2)	8.5652(3)	16.9045(7)	19.4765(9)	95.421(3)	90.539(3)	91.394(3)	0.0379	0.0533	2486	29	9	10	9	1
	LS4	$P2_1/n$	2813.0(2)	8.3888(4)	38.441(2)	8.7642(4)	90	95.543(5)	90								
235	LS3	$P\bar{1}$	2825.4(3)	8.5735(5)	16.959(1)	19.526(1)	95.383(4)	90.588(4)	91.431(4)	0.0603	0.0786	2505	29	9	10	9	1
	LS4	$P2_1/n$	2837.5(3)	8.4150(5)	38.583(2)	8.7836(5)	90	95.735(4)	90								
250	HS1	$P\bar{1}$	1418.9(1)	8.4882(4)	8.5053(4)	19.8644(9)	81.933(4)	87.891(4)	90.119(4)	0.0588	0.0721	3336	39	9	16	13	1
	LS3	$P\bar{1}$	2794.8(2)	8.5470(3)	16.9212(6)	19.4097(7)	95.113(2)	90.118(2)	91.569(2)								
	LS4	$P2_1/n$	2781.0(3)	8.3580(4)	38.207(2)	8.7486(4)	90	95.450(4)	90								
275	HS1	$P\bar{1}$	1412.8(2)	8.4784(6)	8.4820(6)	19.852(1)	82.009(3)	87.943(4)	89.776(4)	0.0630	0.0683	1297	27	9	8	9	1
	HS2	$P4_3$	2820.4(5)	8.4751(6)	8.4751(6)	39.266(4)	90	90	90								
300	HS1	$P\bar{1}$	1419.0(3)	8.4914(8)	8.4987(9)	19.869(2)	81.985(4)	87.999(4)	89.804(4)	0.0451	0.0753	1298	27	9	8	9	1
	HS2	$P4_3$	2832.8(6)	8.4858(8)	8.4858(8)	39.339(4)	90	90	90								
Cycle 2																	
250	HS1	$P\bar{1}$	1406.7(2)	8.4665(7)	8.4689(7)	19.824(2)	82.020(4)	87.879(4)	89.778(3)	0.0638	0.0707	1290	27	9	8	9	1
	HS2	$P4_3$	2807.5(6)	8.4610(7)	8.4610(7)	39.217(4)	90	90	90								
180	LS3	$P\bar{1}$	2804.6(2)	8.5631(3)	16.8984(8)	19.4764(9)	95.427(3)	90.521(3)	91.386(3)	0.0411	0.0578	2490	29	9	10	9	1
	LS4	$P2_1/n$	2808.9(3)	8.3871(5)	38.415(3)	8.7610(5)	90	95.655(6)	90								
235	LS3	$P\bar{1}$	2814.7(2)	8.5519(3)	16.9334(7)	19.5189(8)	95.106(2)	90.137(3)	91.277(3)	0.0610	0.0719	2499	29	9	10	9	1
	LS4	$P2_1/n$	2834.9(6)	8.3577(9)	39.000(6)	8.7385(8)	90	95.59(1)	90								
250	HS1	$P\bar{1}$	1418.4(1)	8.4879(4)	8.5014(4)	19.867(1)	81.918(4)	87.9543(4)	90.097(4)	0.0590	0.0541	3331	39	9	16	13	1
	LS3	$P\bar{1}$	2789.6(2)	8.5409(3)	16.9114(6)	19.3979(7)	95.096(2)	90.181(2)	91.589(2)								
	LS4	$P2_1/n$	2778.7(2)	8.3598(3)	38.191(1)	8.7423(4)	90	95.418(3)	90								

Table S9 continued.

T [K]	Phase	Space Group	*V* [Å^3]	*a* [Å]	*b* [Å]	*c* [Å]	*α* [deg]	*β* [deg]	*γ* [deg]	R_{wp}	$R_{wp'}$	Total Parameters	Parameters Excl Peaks	Background Parameters	Lattice Parameters	Peak Shapes	Zero Point
275	HS1	$P\bar{1}$	1411.8(4)	8.478(1)	8.481(1)	19.842(3)	81.994(4)	87.899(4)	89.760(4)	0.0625	0.0953	1295	27	9	8	9	1
	HS2	$P4_3$	2815(1)	8.467(2)	8.467(2)	39.26(1)	90	90	90								
300	HS1	$P\bar{1}$	1420.6(1)	8.4946(4)	8.5015(4)	19.878(1)	81.978(3)	87.965(3)	89.797(3)	0.0427	0.0689	1298	27	9	8	9	1
	HS2	$P4_3$	2833(1)	8.487(1)	8.487(1)	39.32(1)	90	90	90								
Cycle 3																	
250	HS1	$P\bar{1}$	1408.5(2)	8.4727(8)	8.4736(8)	19.826(2)	81.987(4)	87.840(4)	89.746(4)	0.0643	0.0812	1297	27	9	8	9	1
	HS2	$P4_3$	2806.8(6)	8.4616(7)	8.4616(7)	39.202(4)	90	90	90								
180	LS3	$P\bar{1}$	2805.2(2)	8.5665(3)	16.8970(7)	19.4743(9)	95.442(3)	90.556(2)	91.374(3)	0.0397	0.0572	2491	29	9	10	9	1
	LS4	$P2_1/n$	2810.2(3)	8.3896(5)	38.404(3)	8.7633(5)	90	95.565(6)	90								
235	LS3	$P\bar{1}$	2823.6(2)	8.5642(4)	16.9384(8)	19.544(1)	95.015(3)	90.081(3)	91.257(3)	0.0648	0.0899	2503	29	9	10	9	1
	LS4	$P2_1/n$	2773.8(4)	8.3269(7)	38.292(4)	8.7398(7)	90	95.514(9)	90								
250	HS1	$P\bar{1}$	1417.3(1)	8.4817(4)	8.5120(5)	19.844(1)	81.836(4)	88.008(4)	89.990(4)	0.0617	0.0793	3335	39	9	16	13	1
	LS3	$P\bar{1}$	2795.6(2)	8.5520(3)	16.9283(7)	19.3882(8)	94.892(3)	90.140(3)	91.498(3)								
	LS4	$P2_1/n$	2783.9(3)	8.3681(5)	38.228(2)	8.7452(4)	90	95.662(5)	90								
275	HS1	$P\bar{1}$	1413.6(2)	8.4822(5)	8.4843(5)	19.848(1)	82.002(4)	87.911(5)	89.770(4)	0.0627	0.1046	1295	27	9	8	9	1
	HS2	$P4_3$	2819(2)	8.4664(0)	8.466(2)	39.33(2)	90	90	90								
300	HS1	$P\bar{1}$	1419.1(1)	8.4916(4)	8.4979(4)	19.873(1)	81.985(3)	87.983(3)	89.790(3)	0.0428	0.0645	1298	27	9	8	9	1
	HS2	$P4_3$	2829.2(4)	8.4842(5)	8.4842(5)	39.305(4)	90	90	90								
Cycle 4																	
250	HS1	$P\bar{1}$	1408.8(2)	8.4730(6)	8.4731(6)	19.830(1)	81.995(4)	87.859(4)	89.753(4)	0.0646	0.0888	1295	27	9	8	9	1
	HS2	$P4_3$	2812.1(4)	8.4668(5)	8.4668(5)	39.227(3)	90	90	90								
180	LS3	$P\bar{1}$	2799.4(2)	8.5582(3)	16.8844(6)	19.4662(8)	95.412(3)	90.526(2)	91.320(2)	0.0394	0.0532	2503	29	9	10	9	1
	LS4	$P2_1/n$	2806.1(3)	8.3860(5)	38.384(3)	8.7568(5)	90	95.418(6)	90								
235	LS3	$P\bar{1}$	2831.8(3)	8.5737(5)	16.9515(9)	19.565(1)	95.062(4)	90.191(4)	91.153(4)	0.0691	0.0885	2512	29	9	10	9	1
	LS4	$P2_1/n$	2763.7(3)	8.2977(6)	38.251(3)	8.7500(6)	90	95.658(6)	90								
250	HS1	$P\bar{1}$	1419.4(1)	8.5033(5)	8.4898(4)	19.868(1)	81.996(4)	87.923(5)	89.820(5)	0.0579	0.0934	3333	39	9	16	13	1
	LS3	$P\bar{1}$	2797.2(3)	8.5654(4)	16.9174(9)	19.380(1)	94.871(4)	90.332(4)	91.478(4)								
	LS4	$P2_1/n$	2799.8(3)	8.4307(5)	38.186(3)	8.7388(5)	90	95.632(6)	90								

Table S9 continued.

T [K]	Phase	Space Group	V [Å^3]	a [Å]	b [Å]	c [Å]	α [deg]	β [deg]	γ [deg]	R_{wp}	$R_{wp'}$	Total Parameters	Parameters Excl Peaks	Background Parameters	Lattice Parameters	Peak Shapes	Zero Point
275	HS1	$P\bar{1}$	1413.0(2)	8.4807(5)	8.4827(5)	19.848(1)	81.993(4)	87.900(4)	89.752(4)	0.0642	0.1010	1290	27	9	8	9	1
	HS2	$P4_3$	2810(2)	8.453(2)	8.453(2)	39.32(1)	90	90	90								
300	HS1	$P\bar{1}$	1420.5(1)	8.4952(3)	8.5008(4)	19.8770(9)	81.974(3)	87.977(3)	89.772(3)	0.0420	0.0689	1298	27	9	8	9	1
	HS2	$P4_3$	2833(2)	8.477(2)	8.477(2)	39.43(2)	90	90	90								

Table S10 Rietveld refinement results from the Cu-K_α X-ray powder diffraction data for $[FeL_2][BF_4]_2$ during the thermal cycles in Table S9, and after subsequent annealing at 397 K for 24 hrs (Figure S17). Displacement parameters were not refined during these Rietveld fits.

T [K]	Phase	Space Group	V [Å^3]	a [Å]	b [Å]	c [Å]	α [deg]	β [deg]	γ [deg]	%	R_{wp}	$R_{wp'}$	Total Parameters	Background Param.	Lattice Param.	Peak Shapes	Scale Param..	Zero Point
300	HS1	$P\bar{1}$	1418.8(3)	8.488(1)	8.501(1)	19.870(3)	81.98(1)	87.95(1)	89.92(1)	62.1(5)	0.0878	0.1641	29	9	8	9	2	1
	HS2	$P4_3$	2833.8(6)	8.4928(7)	8.4928(7)	39.289(5)	90	90	90	37.9(5)								
Cycle 1																		
180	LS3	$P\bar{1}$	2796.0(8)	8.559(1)	16.874(2)	19.448(4)	95.31(1)	90.69(1)	91.10(1)	70.9(6)	0.1004	0.1800	31	9	10	9	2	1
	LS4	$P2_1/n$	2802.8(9)	8.379(1)	38.37(1)	8.762(1)	90	95.76(2)	90	29.1(6)								
300	HS1	$P\bar{1}$	1417.5(3)	8.4895(9)	8.4936(9)	19.864(3)	81.973(7)	87.991(8)	89.804(8)	75.3(7)	0.0838	0.1568	29	9	8	9	2	1
	HS2	$P4_3$	2834(1)	8.495(1)	8.495(1)	39.27(1)	90	90	90	24.7(7)								
Cycle 2																		
180	LS3	$P\bar{1}$	2794.2(7)	8.560(1)	16.864(2)	19.445(4)	95.31(1)	90.70(1)	91.159(9)	74.0 (6)	0.1022	0.1831	31	9	10	9	2	1
	LS4	$P2_1/n$	2800.7(9)	8.379(1)	38.35(1)	8.761(1)	90	95.75(2)	90	26.0(6)								
300	HS1	$P\bar{1}$	1418.8(2)	8.4921(8)	8.4967(8)	19.871(2)	81.967(7)	87.988(7)	89.788(7)	78.1(8)	0.0839	0.1565	29	9	8	9	2	1
	HS2	$P4_3$	2838(1)	8.499(1)	8.499(1)	39.29(2)	90	90	90	21.9(8)								

Table S10 continued.

T [K]	Phase	Space Group	V [Å^3]	a [Å]	b [Å]	c [Å]	α [deg]	β [deg]	γ [deg]	%	R_{wp}	$R_{wp'}$	Total Parameters	Background Param.	Lattice Param.	Peak Shapes	Scale Param..	Zero Point
Cycle 3																		
180	LS3	$P\bar{1}$	2794.0(7)	8.5618(9)	16.860(2)	19.446(4)	95.309(9)	90.71(1)	91.196(9)	75.2(5)	0.1010	0.1813	31	9	10	9	2	1
	LS4	$P2_1/n$	2801(1)	8.379(1)	38.34(1)	8.761(1)	90	95.73(2)	90	24.8(5)								
300	HS1	$P\bar{1}$	1418.4(2)	8.4911(8)	8.4962(8)	19.868(2)	81.972(6)	87.980(7)	89.783(7)	81.2(8)	0.0857	0.1600	29	9	8	9	2	1
	HS2	$P4_3$	2838(2)	8.499(2)	8.499(2)	39.29(2)	90	90	90	18.8(8)								
Cycle 4																		
180	LS3	$P\bar{1}$	2792.8(7)	8.5615(9)	16.854(2)	19.444(3)	95.306(9)	90.72(1)	91.217(8)	76.3(5)	0.1008	0.1804	31	9	10	9	2	1
	LS4	$P2_1/n$	2799(1)	8.378(1)	38.33(1)	8.760(1)	90	95.74(2)	90	23.7(5)								
300	HS1	$P\bar{1}$	1419.6(2)	8.4935(8)	8.4986(8)	19.874(2)	81.966(6)	87.985(7)	89.778(7)	78.7(8)	0.0858	0.1592	29	9	8	9	2	1
	HS2	$P4_3$	2843(3)	8.501(2)	8.501(2)	39.34(3)	90	90	90	21.3(8)								
Before annealing																		
300	HS1	$P\bar{1}$	1419.2(2)	8.4934(7)	8.4982(7)	19.870(2)	81.955(6)	87.974(6)	89.768(6)	79.7(8)	0.0795	0.1520	29	9	8	9	2	1
	HS2	$P4_3$	2839(2)	8.500(2)	8.500(2)	39.30(3)	90	90	90	20.3(8)								
After annealing																		
300	HS1	$P\bar{1}$	1415.9(3)	8.4849(8)	8.4930(8)	19.856(2)	81.937(7)	87.973(7)	89.796(7)	79.1(9)	0.0846	0.1671	29	9	8	9	2	1
	HS2	$P4_3$	2832(3)	8.494(2)	8.494(2)	39.25(3)	90	90	90	20.9(9)								

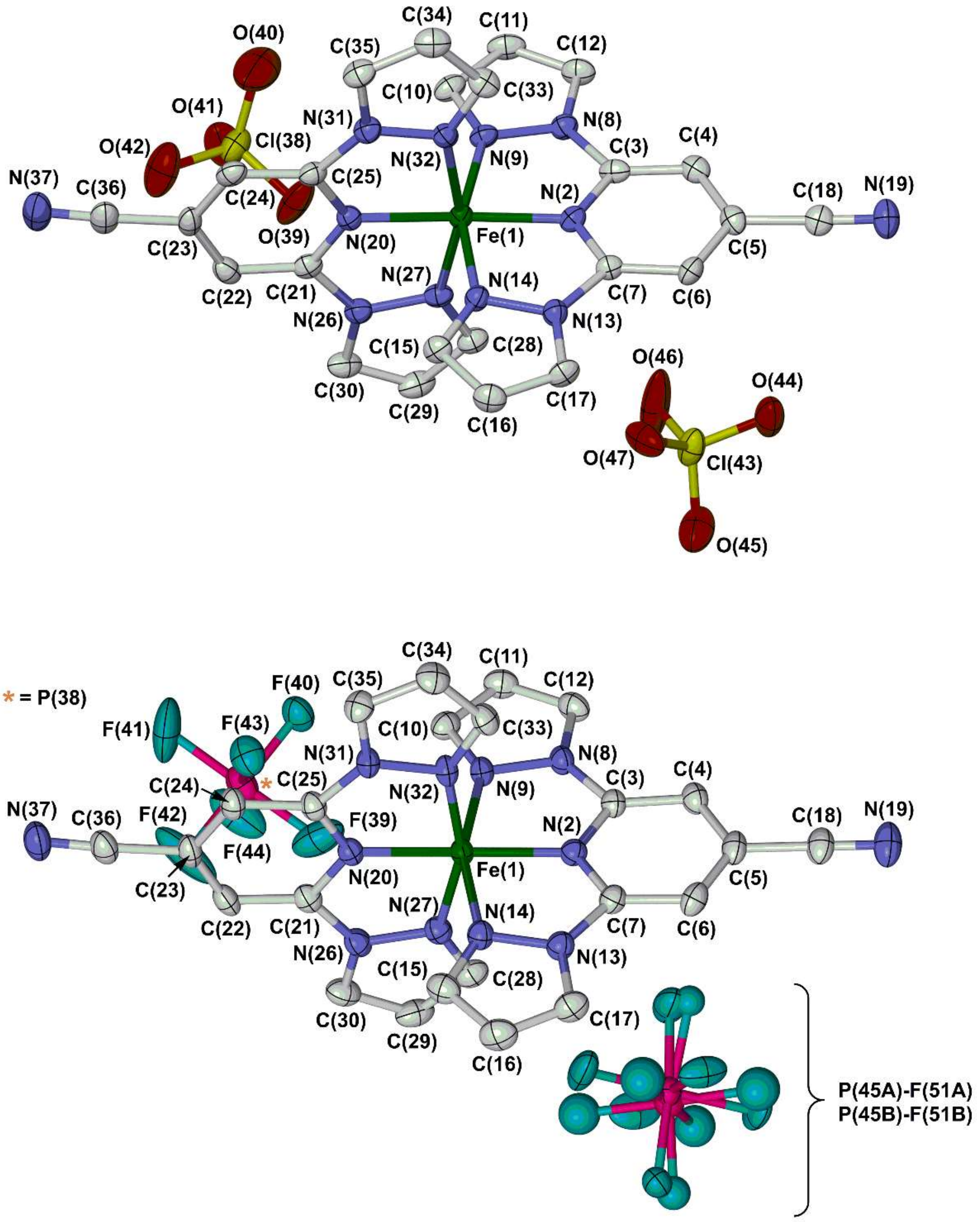

Figure S18 The asymmetric units of isomorphous $[FeL_2][ClO_4]_2$ (top) and $[FeL_2][PF_6]_2$ (bottom), showing the full atom numbering scheme. Displacement ellipsoids are at the 50 % probability level, and H atoms are omitted for clarity.

Color code: C, white; Cl, yellow; F, cyan; Fe, green; N, blue; O, red; P, pink.

A room temperature crystal structure of $[FeL_2][ClO_4]_2$ is included in ref. [1].

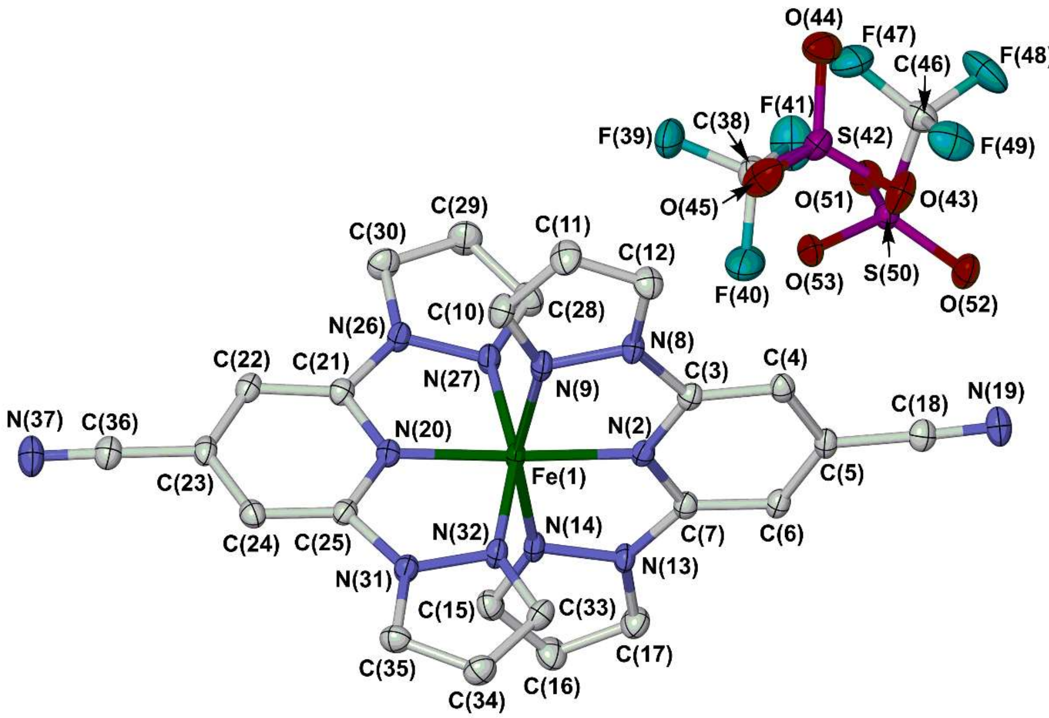

Figure S19 The asymmetric unit of $[FeL_2][CF_3SO_3]_2$, showing the full atom numbering scheme. Displacement ellipsoids are at the 50 % probability level, and H atoms are omitted for clarity.

Color code: C, white; F, cyan; Fe, green; N, blue; O, red; S, purple.

Table S11 Selected bond lengths [Å] and angles [deg] for the other salts of $[FeL_2]^{2+}$. See Figures S18 and S19 for the atom numbering scheme, while definitions of the structural parameters at the foot of the Table are on page S9.

	$[FeL_2][ClO_4]_2$[a]	$[FeL_2][PF_6]_2$	$[FeL_2][CF_3SO_3]_2$
Fe(1)–N(2)	1.890(4)	1.886(2)	1.8967(16)
Fe(1)–N(9)	1.983(4)	1.972(2)	1.9677(19)
Fe(1)–N(14)	1.961(4)	1.965(2)	1.9748(18)
Fe(1)–N(20)	1.892(4)	1.882(2)	1.8944(17)
Fe(1)–N(27)	1.952(4)	1.969(2)	1.9730(17)
Fe(1)–N(32)	1.955(4)	1.965(2)	1.9704(17)
N(2)–Fe(1)–N(9)	80.12(16)	80.50(9)	80.38(7)
N(2)–Fe(1)–N(14)	80.66(16)	80.44(9)	80.16(7)
N(2)–Fe(1)–N(20) (φ)	175.58(17)	177.08(10)	177.48(7)
N(2)–Fe(1)–N(27)	100.91(17)	101.82(9)	98.23(7)
N(2)–Fe(1)–N(32)	98.09(16)	97.54(9)	101.55(7)
N(9)–Fe(1)–N(14)	160.75(16)	160.86(9)	160.52(7)
N(9)–Fe(1)–N(20)	104.10(16)	101.67(9)	97.75(7)
N(9)–Fe(1)–N(27)	90.65(16)	89.80(9)	90.22(7)
N(9)–Fe(1)–N(32)	92.53(16)	92.29(9)	93.19(7)
N(14)–Fe(1)–N(20)	95.14(16)	97.43(9)	101.72(7)
N(14)–Fe(1)–N(27)	91.98(16)	92.30(9)	93.36(7)
N(14)–Fe(1)–N(32)	91.17(16)	92.01(9)	89.90(7)
N(20)–Fe(1)–N(27)	80.50(17)	80.21(9)	80.04(7)
N(20)–Fe(1)–N(32)	80.55(16)	80.49(9)	80.19(7)
N(27)–Fe(1)–N(32)	161.00(16)	160.61(9)	160.22(7)
θ	88.52(3)	87.604(19)	85.529(18)

[a] A room temperature structure determination of this compound is reported in ref. [1].

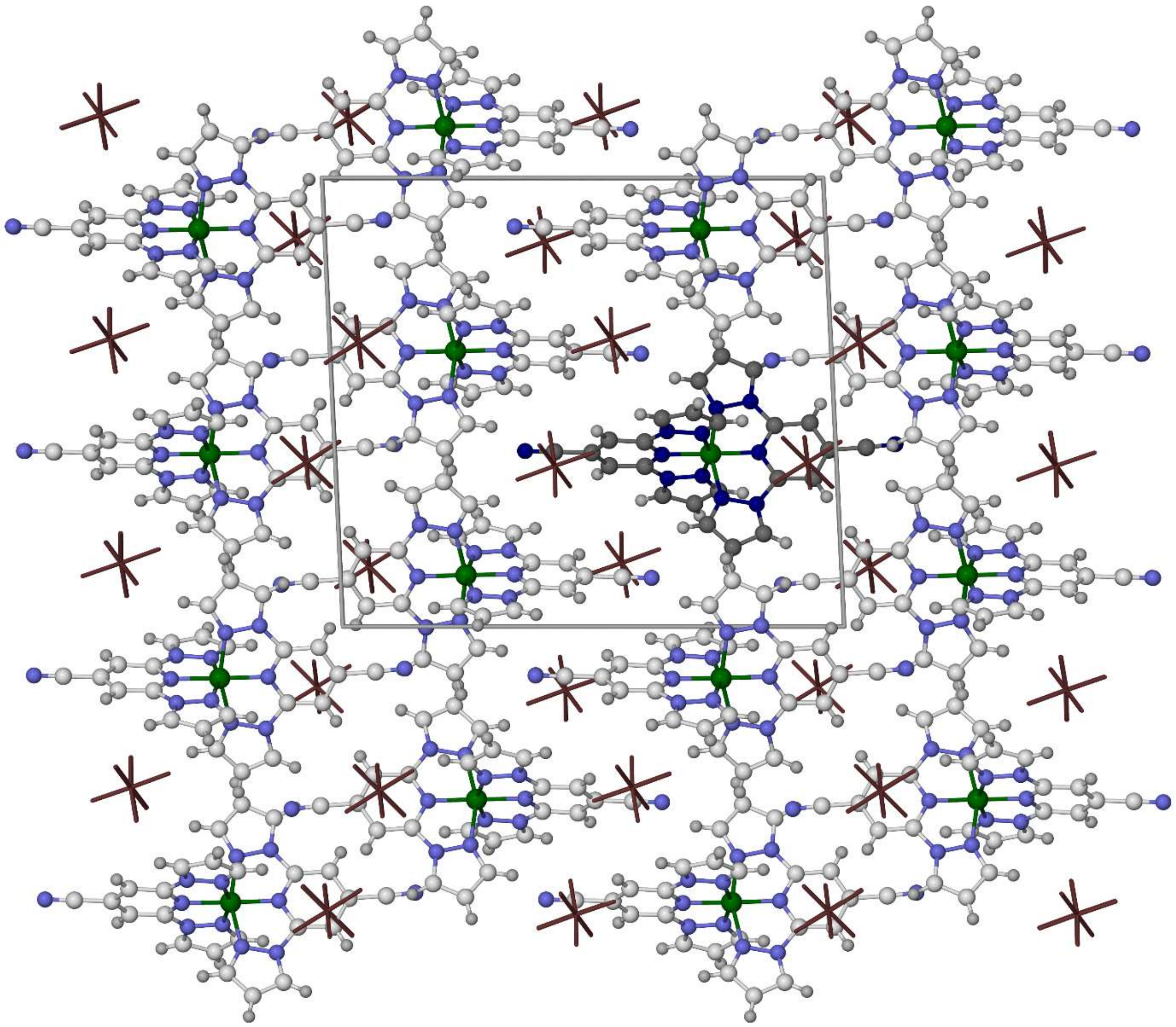

Figure S20 Packing diagram of $[FeL_2][PF_6]_2$, viewed parallel to the [010] crystal vector, with *a* horizontal. One cation is highlighted with dark coloration, and the PF_6^- ions are de-emphasized for clarity; only the major orientation of the disordered anion is included.

Color code: C, white or dark gray; H, pale gray; Fe, green; N, pale or dark blue; PF_6^-, brown.

While this lattice contains layers of cations in the (100) plane, it does not involve four-fold "terpyridine embrace" interdigitation of the cation pyrazolyl side-arms (Figure S21).

The crystal packing in $[FeL_2][ClO_4]_2$, which is isomorphous with this crystal, is described in ref. [1].

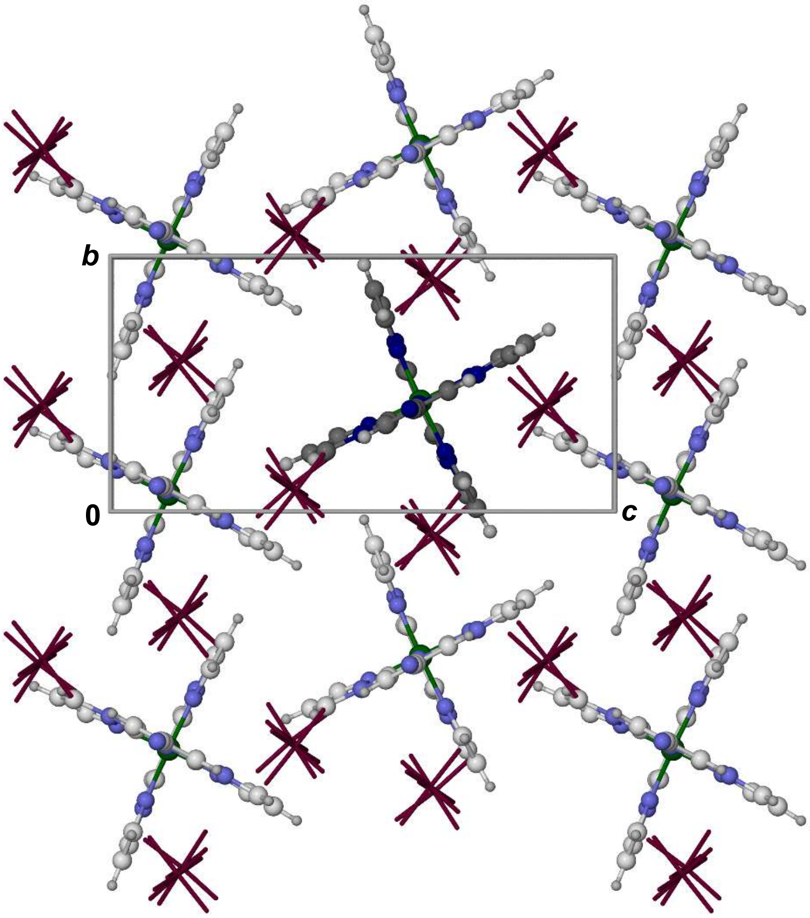

Figure S21 View of the nearest neighbor molecules in a cation layer in $[FeL_2][PF_6]_2$, viewed along the [100] crystal vector. Details as for Figure S20.

Color code: C, white or dark gray; H, pale gray; Fe, green; N, pale or dark blue; PF_6^-, brown.

The crystal packing in $[FeL_2][ClO_4]_2$, which is isomorphous with this crystal, is described in ref. [1].

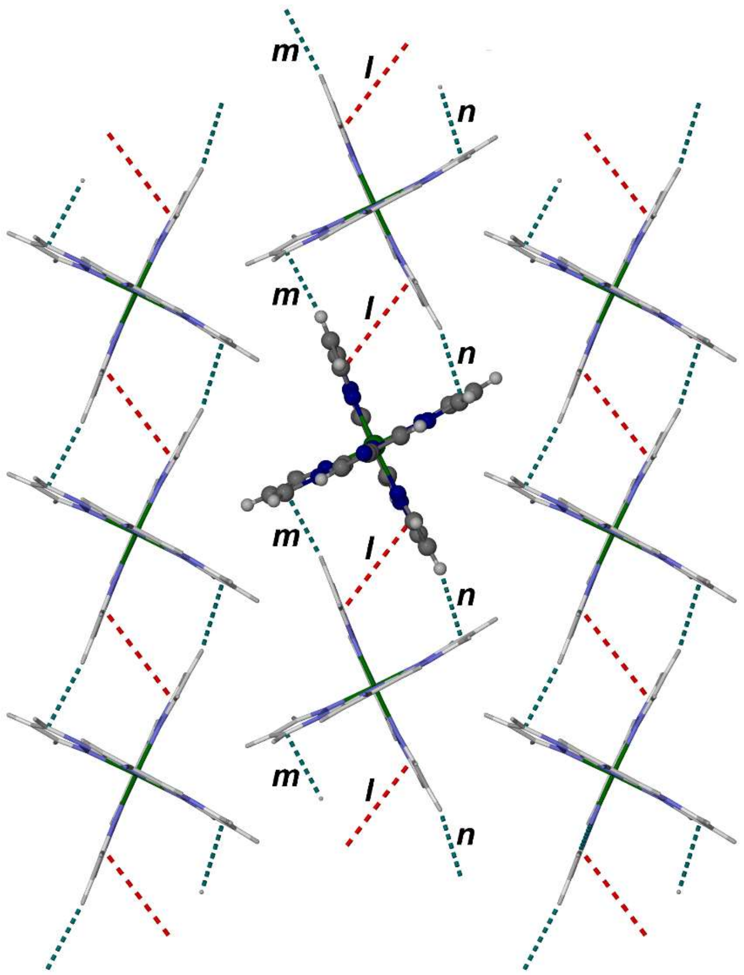

Figure S22 Intermolecular $\pi\cdots\pi$ (red) and C–H$\cdots\pi$ (cyan) contacts in $[FeL_2][PF_6]_2$. The view is the same as in Figure S21. One cation is shown in dark coloration with 50 % displacement ellipsoids, while their neighbor molecules are de-emphasized for clarity. The letter labels for each interaction correspond to those in Table S12.

Color code: C, white or dark gray; H, pale gray; Fe, green; N, pale or dark blue.

Table S12 Intermolecular interactions within the cation layers in the crystal structure of $[FeL_2][PF_6]_2$ [Å, deg]. The letter labels correspond to those in Figure S22, while the atom numbering is shown in Figure S18. Italicized values are for the corresponding interactions in the isomorphous $[FeL_2][ClO_4]_2$ crystal.[a]

$\pi\cdots\pi$ interaction	Dihedral angle [°]	Interplanar distance [Å]	Horizontal offset [Å]	
[N(8)–C(12)]⋯[N(13^{vi})–C(17^{vi})] (***l***)	8.42(15) [*5.9(3)*]	3.837(9) [*3.626(16)*]	1.68 [*1.71*]	
**C–H⋯π interactions**	**C–H [Å]**	**H⋯X [Å]**	**C⋯X [Å]**	**C–H⋯X [°]**
C(11A)–H(11A)⋯[C(33^{vi}), C(34^{vi})] (***m***)	0.95 [*0.95*]	2.94 [*3.00*]	3.89 [*3.95*]	173.3 [*171.9*]
C(16A)–H(16A)⋯[C(28^{vii}), C(29^{vii})] (***n***)	0.95 [*0.95*]	2.80 [*2.76*]	3.73 [*3.70*]	166.6 [*170.4*]

[a] Symmetry codes: (vi) *x*, 1+*y*, *z*; (vii) *x*, –1+*y*, *z*.

These interactions are consistently longer than in the $[FeL_2][BF_4]_2$ LS3 structure (Table S4).

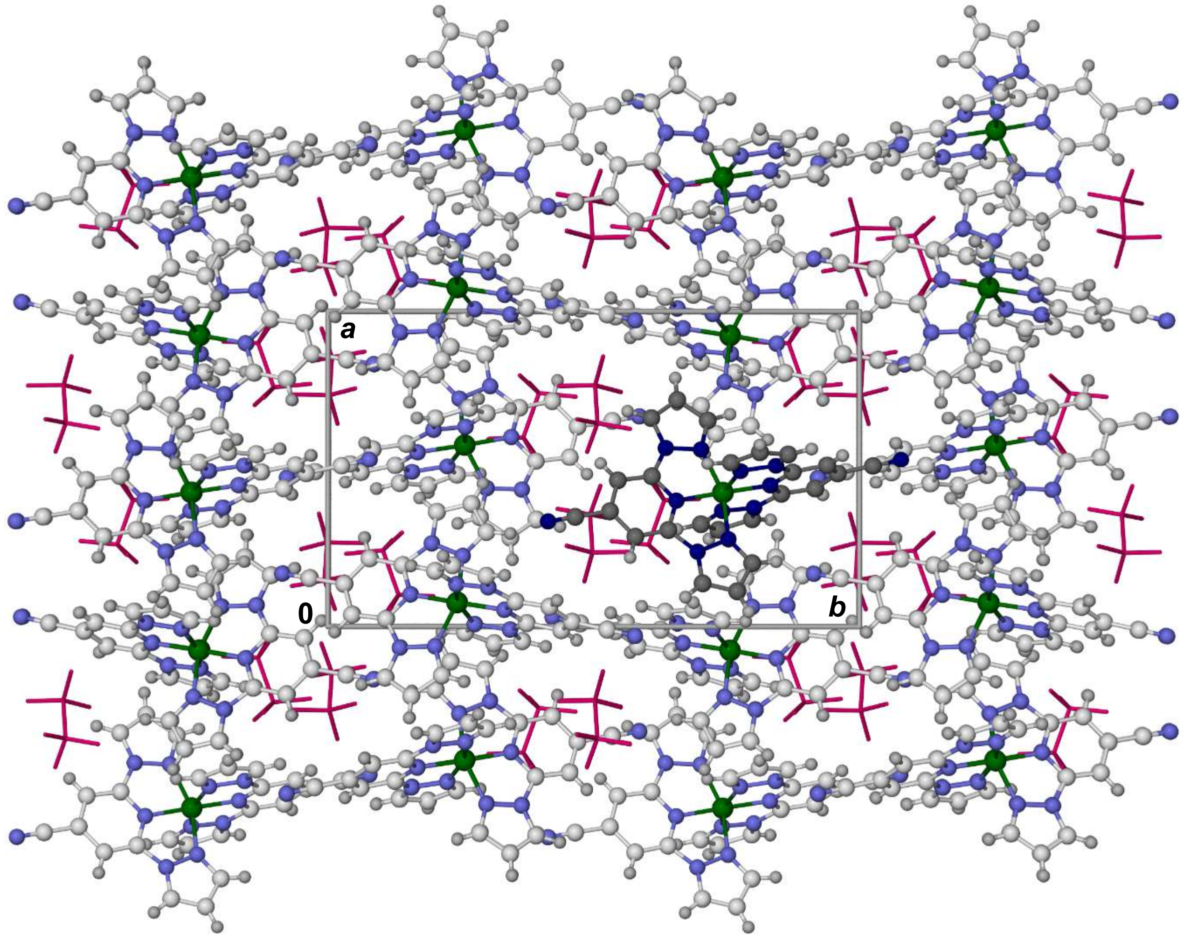

Figure S23 Packing diagram of $[FeL_2][CF_3SO_3]_2$ at 120 K, viewed parallel to the [001] crystal vector. One cation is highlighted with dark coloration, and the triflate ions being de-emphasized for clarity.

Color code: C, white or dark gray; H, pale gray; Fe, green; N, pale or dark blue; $CF_3SO_3^-$, pink.

While this structure also contains layers of cations in the (010) plane, it does not involve four-fold "terpyridine embrace" interdigitation of the cation pyrazolyl side-arms (Figure S24).

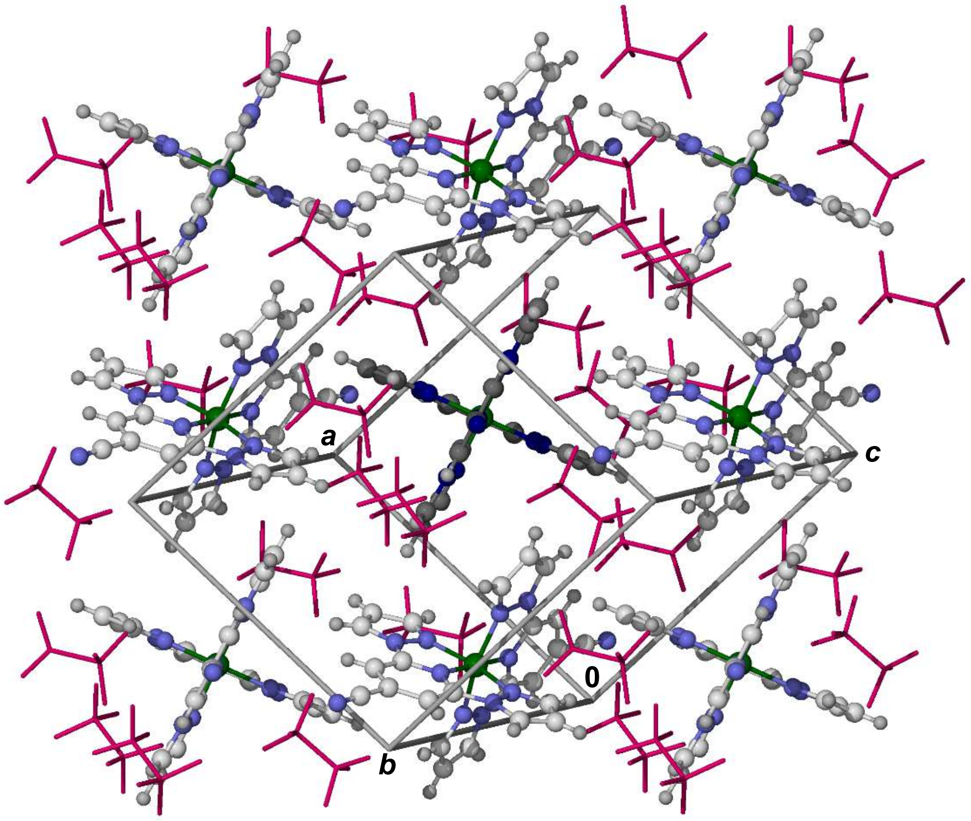

Figure S24 View of the nearest neighbor molecules in a cation layer in $[FeL_2][CF_3SO_3]_2$ at 120 K. The view is arbitrary, and is chosen to emphasize that neighboring cations in the layers are strongly canted with respect to each other. Other details as for Figure S23.

Color code: C, white or dark gray; H, pale gray; Fe, green; N, pale or dark blue; $CF_3SO_3^-$, pink.

There are no short intermolecular $\pi\cdots\pi$ or C–H$\cdots\pi$ contacts between the molecules in this structure.

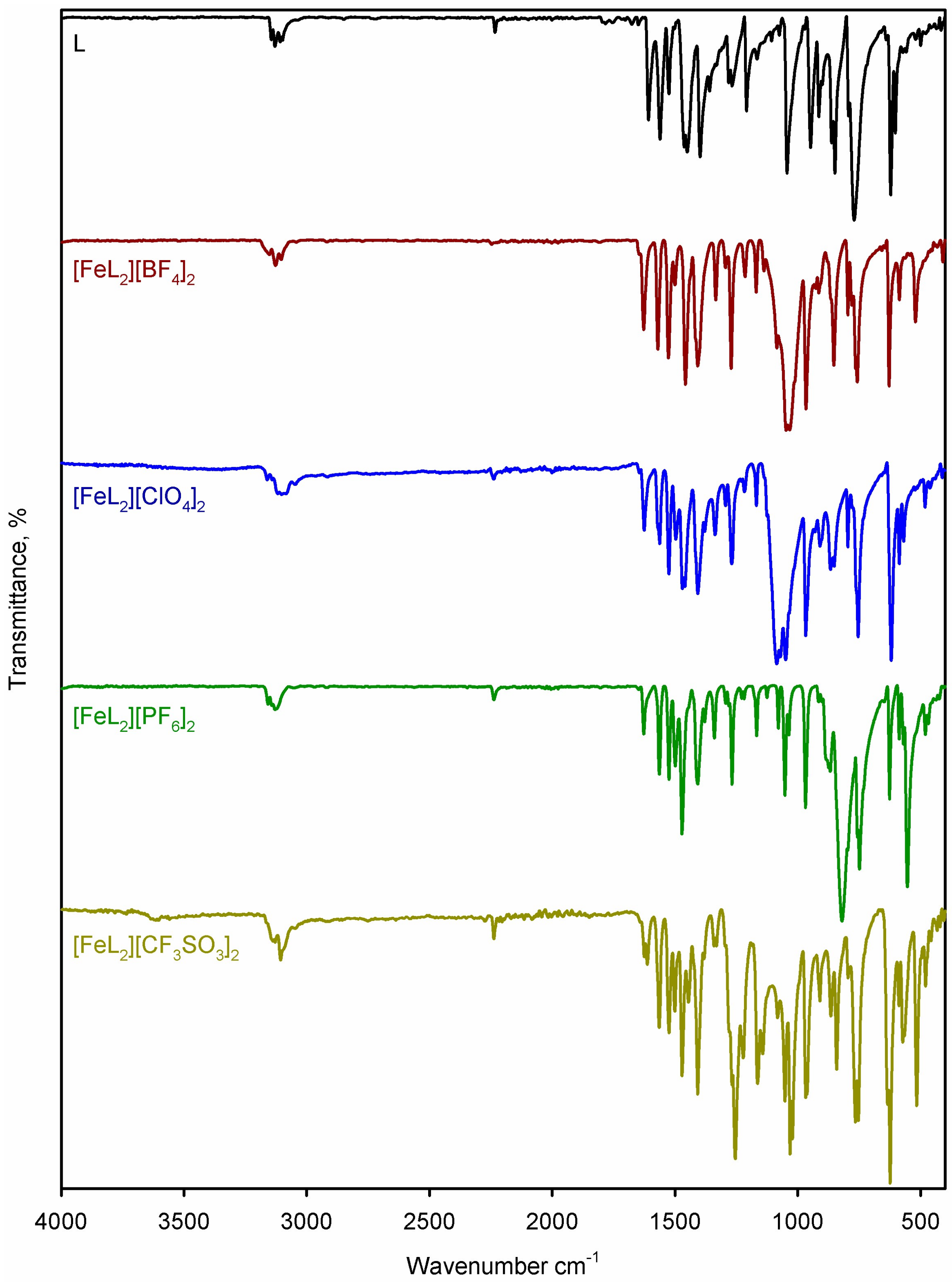

Figure S25 Comparison of the IR spectra of L (black) and the iron complex salts.

The ν\{C≡N\} vibration is too weak to measure accurately in $[FeL_2][BF_4]_2$, possibly because it contains a distribution of peaks arising from the polymorphs in the sample. All the other salts show ν\{C≡N\} = 2238 cm^{-1}, which is 5 cm^{-1} higher than the uncoordinated ligand.

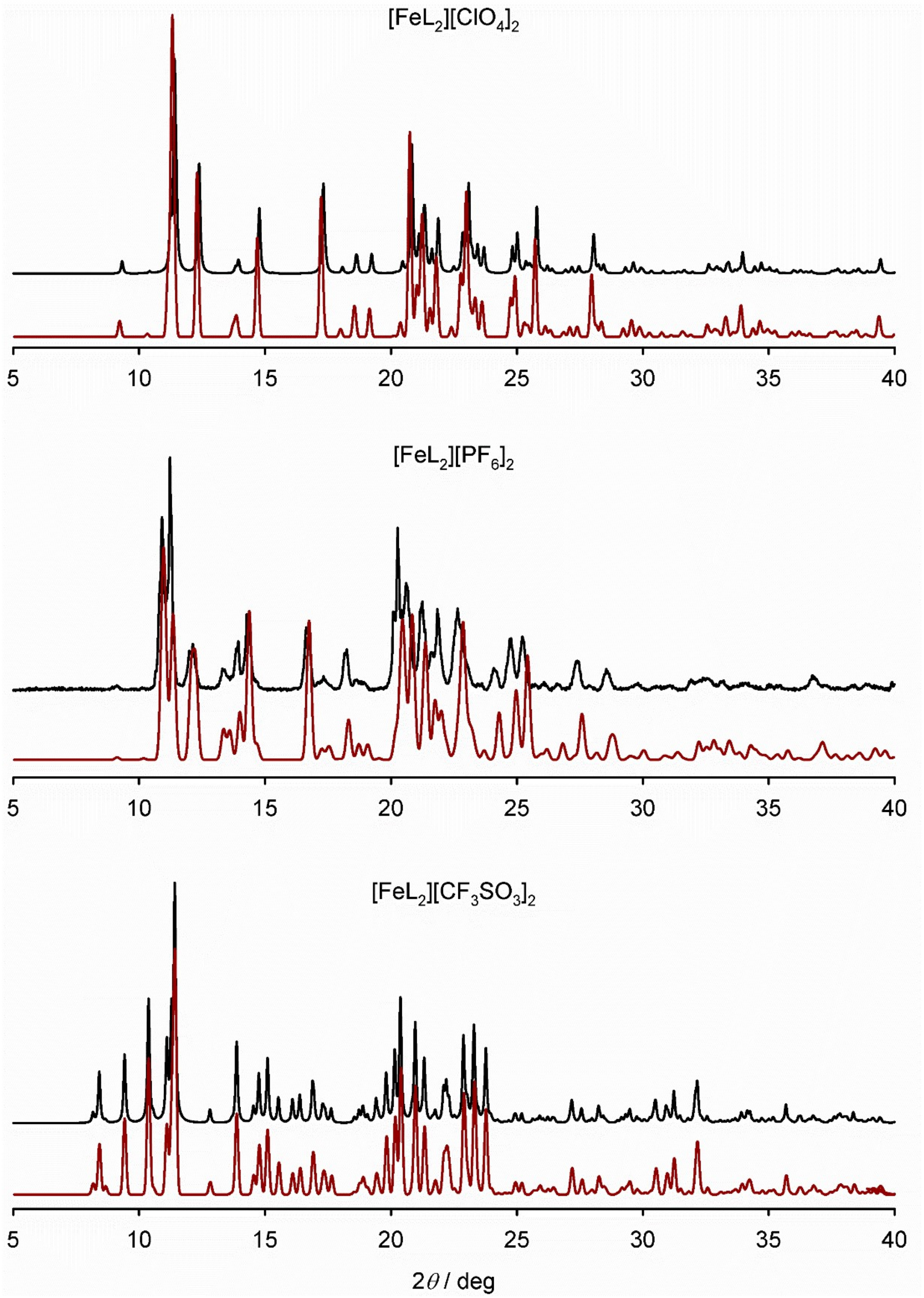

Figure S26 X-ray powder diffraction data for polycrystalline $[FeL_2][ClO_4]_2$, $[FeL_2][PF_6]_2$ and $[FeL_2][CF_3SO_3]_2$ at 290 K (black), and simulations from the single crystal structures of the compounds (red).

The simulation of $[FeL_2][ClO_4]_2$ is calculated from the room temperature crystal structure in ref. [1], which is a better match for the data than the low-temperature refinement in this study. That powder pattern is an excellent match for its crystallographic simulation, but is quite different from the SCO-active phase of $[FeL_2][ClO_4]_2$ reported in ref. [1].

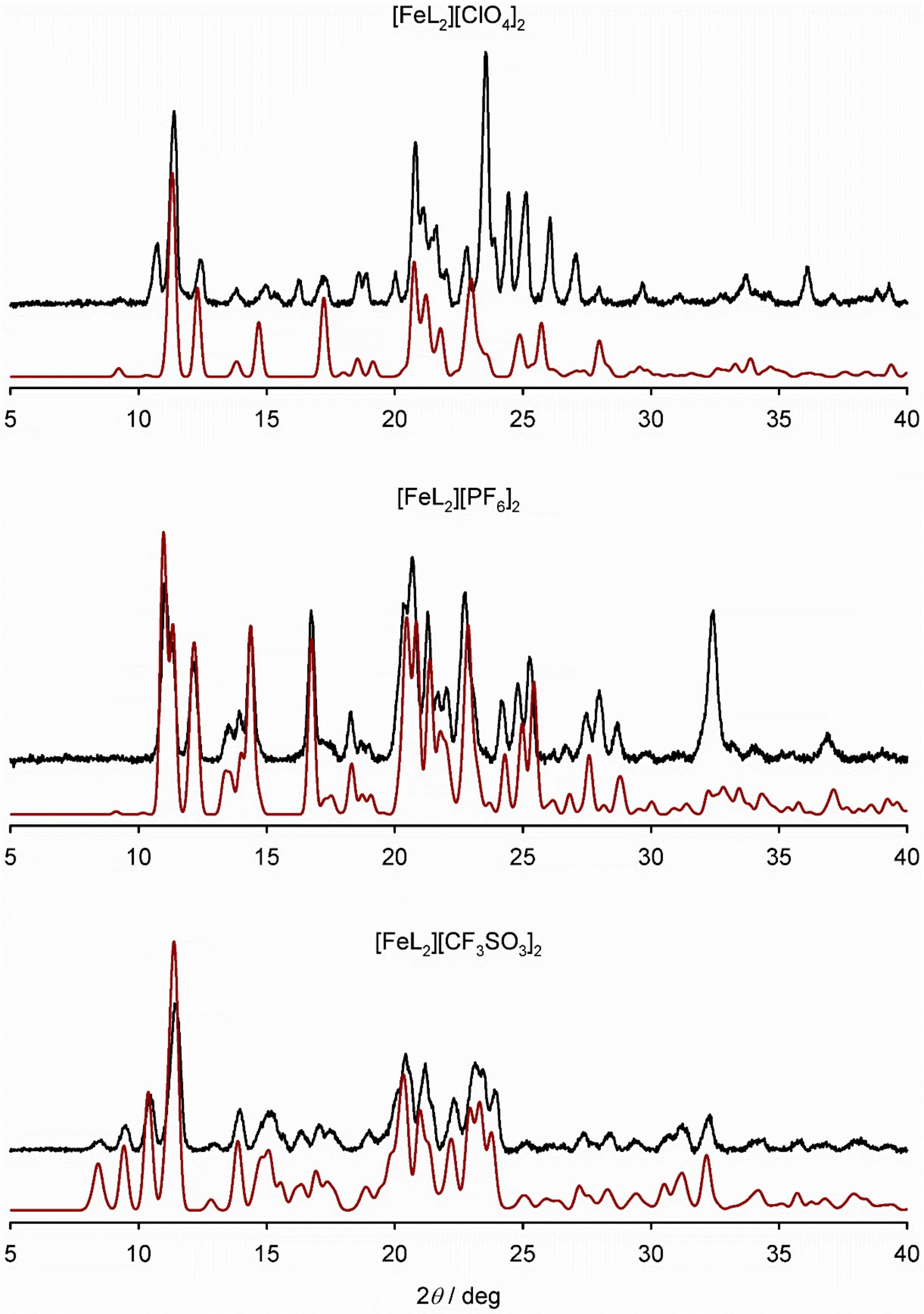

Figure S27 X-ray powder diffraction data for rapidly precipitated powder samples of $[FeL_2][ClO_4]_2$, $[FeL_2][PF_6]_2$ and $[FeL_2][CF_3SO_3]_2$ at 290 K (black), and simulations from the single crystal structures of the compounds (red).

The rapidly precipitated PF_6^- and triflate salts are isomorphous with the polycrystalline materials, and are apparently phase-pure (Figure S26). However $[FeL_2][ClO_4]_2$ shows some additional diffraction peaks that are not predicted by the crystallographic simulation, implying that sample contains a minor contaminent phase. That is supported by the magnetic data from this material (Figure S29).

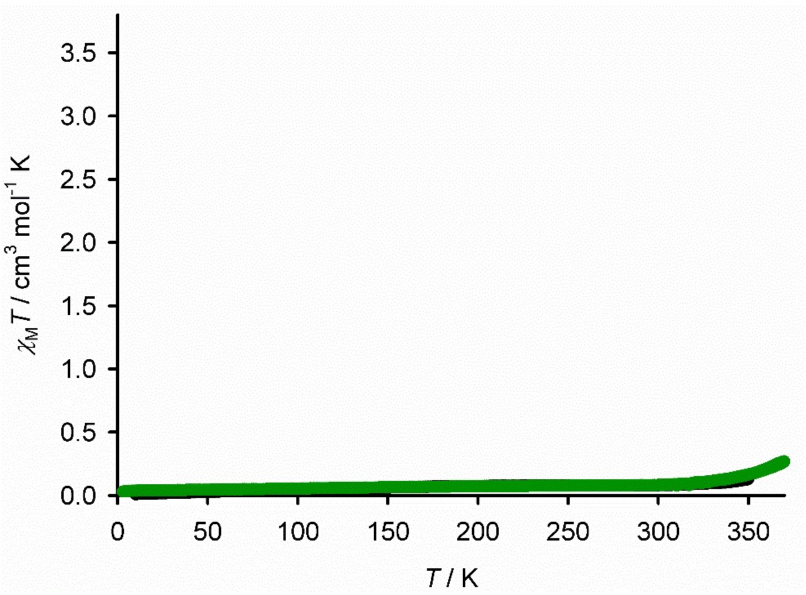

Figure S28 Variable temperature magnetic susceptibility data for crystalline $[FeL_2][PF_6]_2$ (black) and $[FeL_2][CF_3SO_3]_2$ (green). Data for the PF_6^- salt were measured on a 300→5→350→300 K temperature cycle, while the triflate salt was measured similarly but up to 370 K.

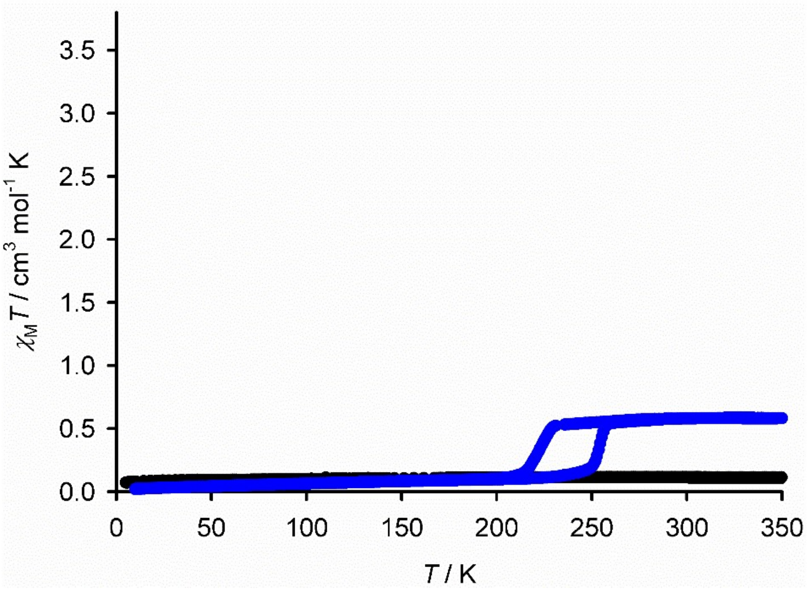

Figure S29 Variable temperature magnetic susceptibility data for crystalline (black) and powder (blue) samples of $[FeL_2][ClO_4]_2$, measured on a 300→5→350→300 K temperature cycle. Scan rate 5 K min^{-1}.

The single crystalline material is low-spin but the powder appears to contain a second phase, with an SCO-active phase comprising *ca* 20 % of the sample. That is consistent with the single crystal structure in this study, and with the X-ray powder diffraction data from the two samples (Figures S26 and S27).

The powder pattern and magnetic data from the pure SCO-active phase are reported in ref. [1].

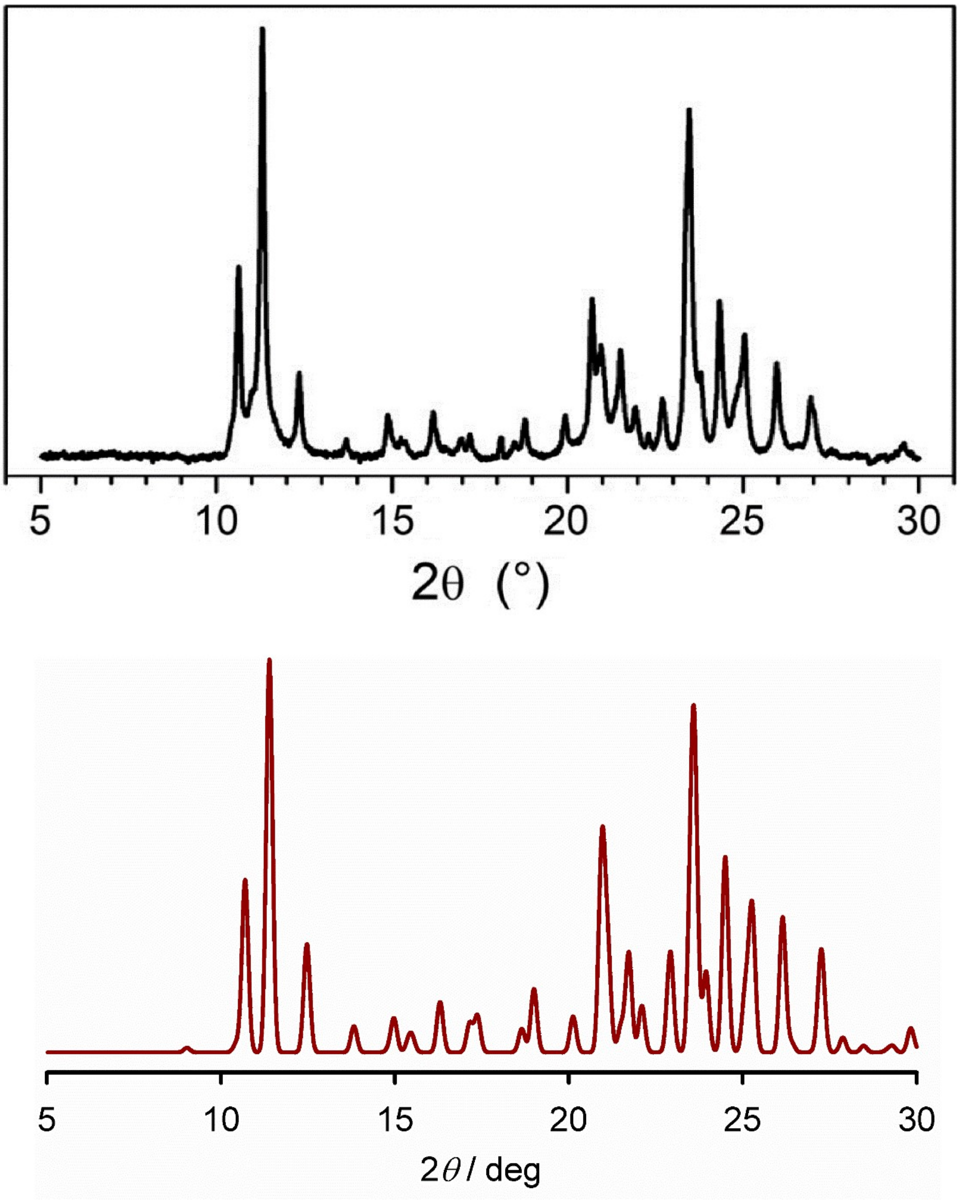

Figure S30 Top: literature room temperature X-ray powder diffraction data for the phase-pure SCO-active form of $[FeL_2][ClO_4]_2$.[1] Bottom: calculated powder pattern from a model derived from the HS2 $P4_3$ phase of $[FeL_2][BF_4]_2$, with the BF_4^- ions replaced by ClO_4^- ions.

The literature data are a poor match for the low-spin single crystal phase of $[FeL_2][ClO_4]_2$ (Figure S26), but agree well with the simulation based on the $P4_3$ phase from this work. Hence, the SCO active form of $[FeL_2][ClO_4]_2$ corresponds to the $P4_3$ phase of that salt, not the single crystal material as previously reported.

Interestingly, magnetic data in ref. [1] show the spin-transition in $[FeL_2][ClO_4]_2$ does not change upon multiple scanning. Hence, the HS2 phase of $[FeL_2][ClO_4]_2$ does not appear to exhibit the bifurcated phase behavior shown by the BF_4^- salt.

The literature data are adapted by permission from ref. [1]. Copyright the Royal Society of Chemistry.

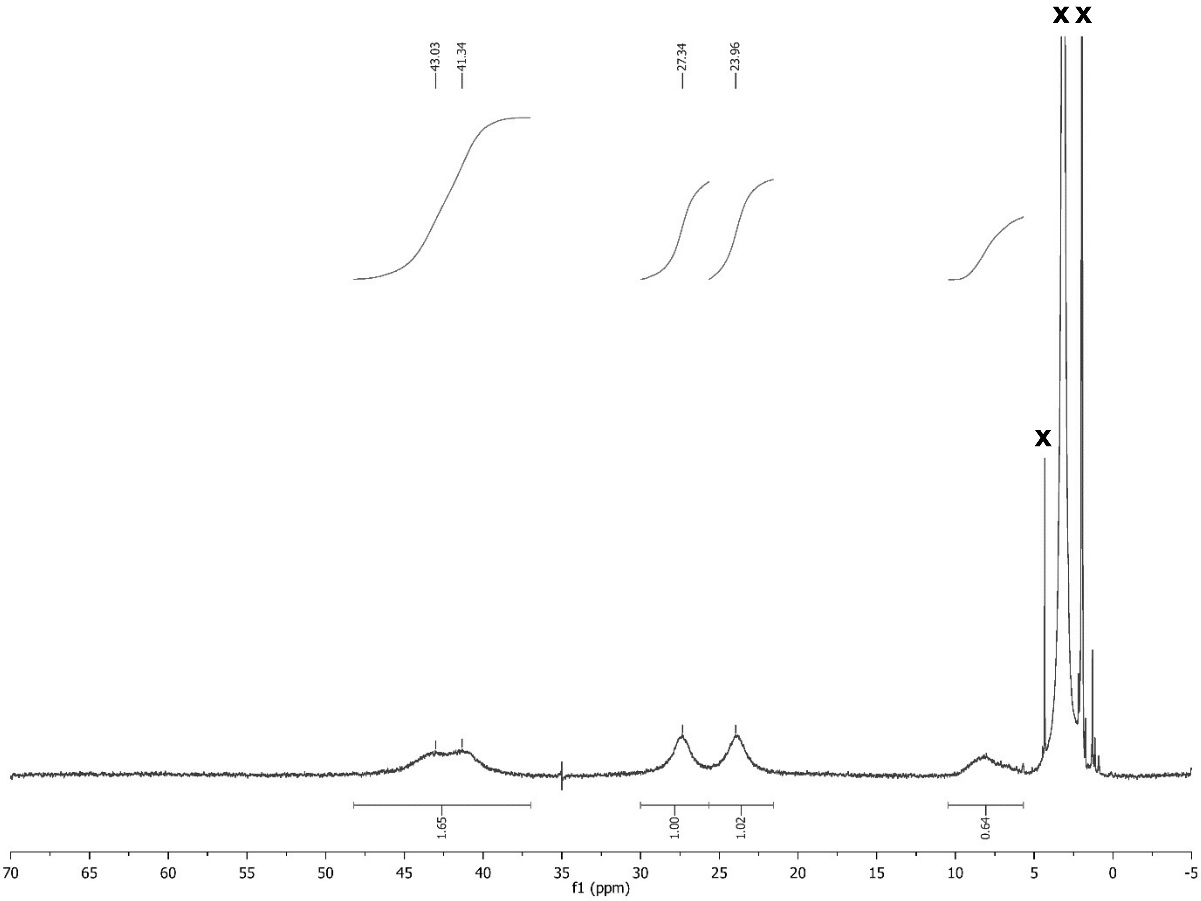

Figure S31 ^{1}H NMR spectrum of $[FeL_2][PF_6]_2$ (CD_3CN). The feature at 35 ppm is a spectrometer artefact.

The four contact shifted peaks are consistent with a C_2- or *m*-symmetric L environment, as expected in $[FeL_2]^{2+}$. The contact shifts are consistent with a $[Fe(bpp)_2]^{2+}$ derivative with a mixed high:low-spin population at room temperature;[28,29] a fully high-spin complex would exhibit two peaks at 60-70 ppm and two peaks at 40-45 ppm.[28-30]

The broad feature between 6-10 ppm may indicate the presence of uncoordinated L in the solution (Figure S1). If so, *ca* 18 % of the ligand in the sample is displaced from the metal centres by integration. We propose this indicates a slow ligand dissociation equilibrium in solution promoted by the peripheral nucleophilic nitrile donors in $[FeL_2]^{2+}$.

Similar solution behavior is also evident in the NMR spectrum of another $[Fe(bpp)_2]^{2+}$ derivative with nucleophilic peripheral substituents.[31]

Unfortunately salts of $[FeL_2]^{2+}$ are too insoluble for measurement of their solution magnetic properties.

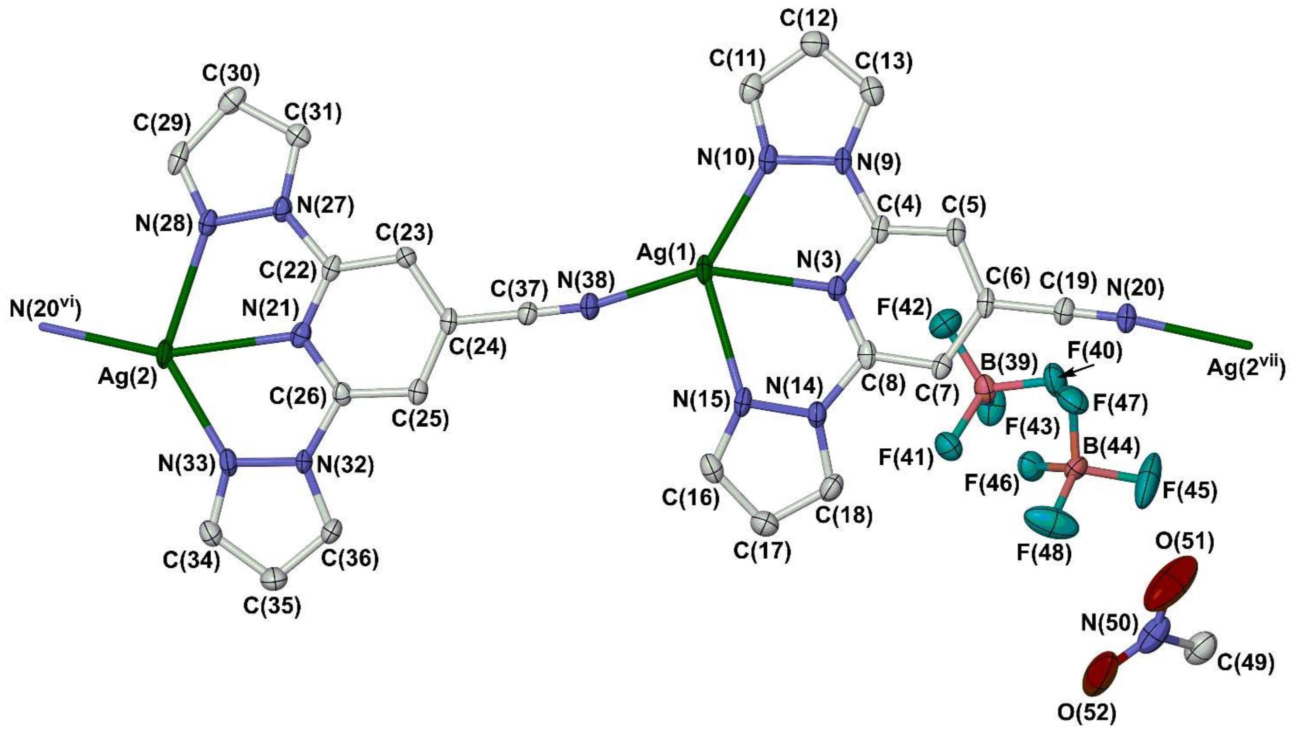

Figure S32 The asymmetric unit of [Ag(μ-L)]BF_4·0.5$MeNO_2$, showing the full atom numbering scheme. Displacement ellipsoids are at the 50 % probability level, and H atoms are omitted for clarity. Symmetry codes: (vi) x–2, y, z–1; (vii) x+2, y, z+1.

Color code: C, white; Ag, green; B, pink; F, cyan; N, blue; O, red.

Table S13 Selected bond lengths [Å] and angles [deg] for [Ag(μ-L)]BF_4·0.5$MeNO_2$. See Figure S32 for the atom numbering scheme.[a]

Ag(1)–N(3)	2.429(3)	Ag(2)–N(20^{viii})	2.261(4)
Ag(1)–N(10)	2.299(4)	Ag(2)–N(21)	2.520(4)
Ag(1)–N(15)	2.530(4)	Ag(2)–N(28)	2.491(4)
Ag(1)–N(38)	2.196(4)	Ag(2)–N(33)	2.300(4)
N(3)–Ag(1)–N(10)	68.23(13)	N(20^{viii})–Ag(2)–N(21)	158.94(13)
N(3)–Ag(1)–N(15)	65.20(12)	N(20^{viii})–Ag(2)–N(28)	95.98(13)
N(3)–Ag(1)–N(38)	154.48(14)	N(20^{viii})–Ag(2)–N(33)	133.17(14)
N(10)–Ag(1)–N(15)	133.14(12)	N(21)–Ag(2)–N(28)	63.60(12)
N(10)–Ag(1)–N(38)	136.88(14)	N(21)–Ag(2)–N(33)	66.53(12)
N(15)–Ag(1)–N(38)	89.97(13)	N(28)–Ag(2)–N(33)	130.02(12)

[a] Symmetry code: (viii) x–2, y, z–1.

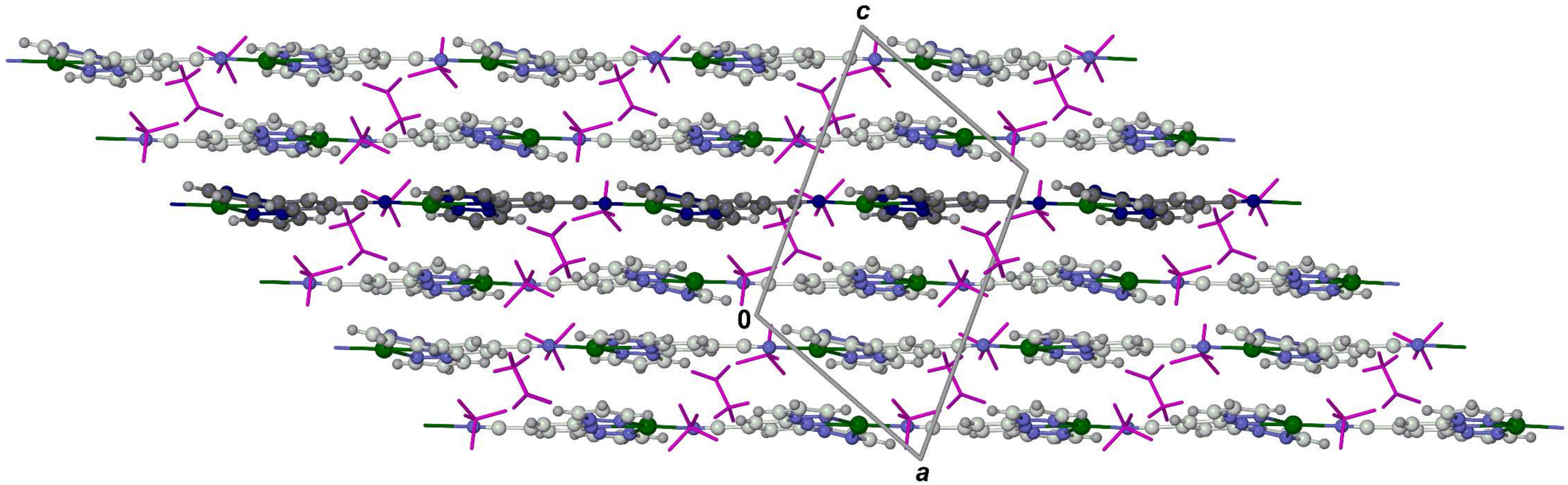

Figure S33 Packing diagram of [Ag(μ-L)]$BF_4 \cdot 0.5MeNO_2$, viewed parallel to the [010] crystal vector. One coordination polymer is highlighted with dark coloration, and the BF_4^- ions and nitromethane molecules are de-emphasized for clarity.

Color code: C{complex}, white or dark gray; H, pale gray; Ag, green; N{complex}, pale or dark blue; BF_4^-, and $MeNO_2$, pink.

The coordination polymer chains propagate by translation along the [201] lattice vector, and associate further *via* crystallographic inversion symmetry into 1D $\pi \cdots \pi$ stacks along *c*. Adjacent polymer chains within the stacks are co-parallel by symmetry, and separated by the interplanar distance 3.243(3) Å. There are no noteworthy Ag···Ag contacts in the structure.

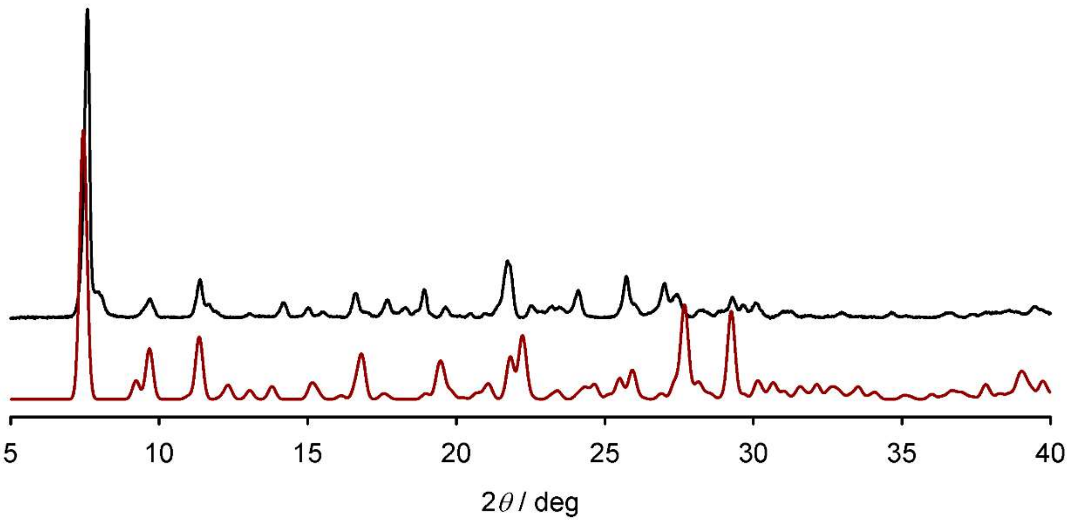

Figure S34 Powder X-ray diffraction data for polycrystalline [Ag(μ-L)]BF_4 at 290 K (black), and a simulation from the single crystal structures of its nitromethane solvate (red).

Agreement between the data and the simulation is good, but not perfect. That could reflect structural changes following loss of lattice solvent from the sample. The material contains 0.5 equiv nitromethane when freshly crystallized, but this is replaced by *ca* 0.5 equiv H_2O in air-dried samples by microanalysis.

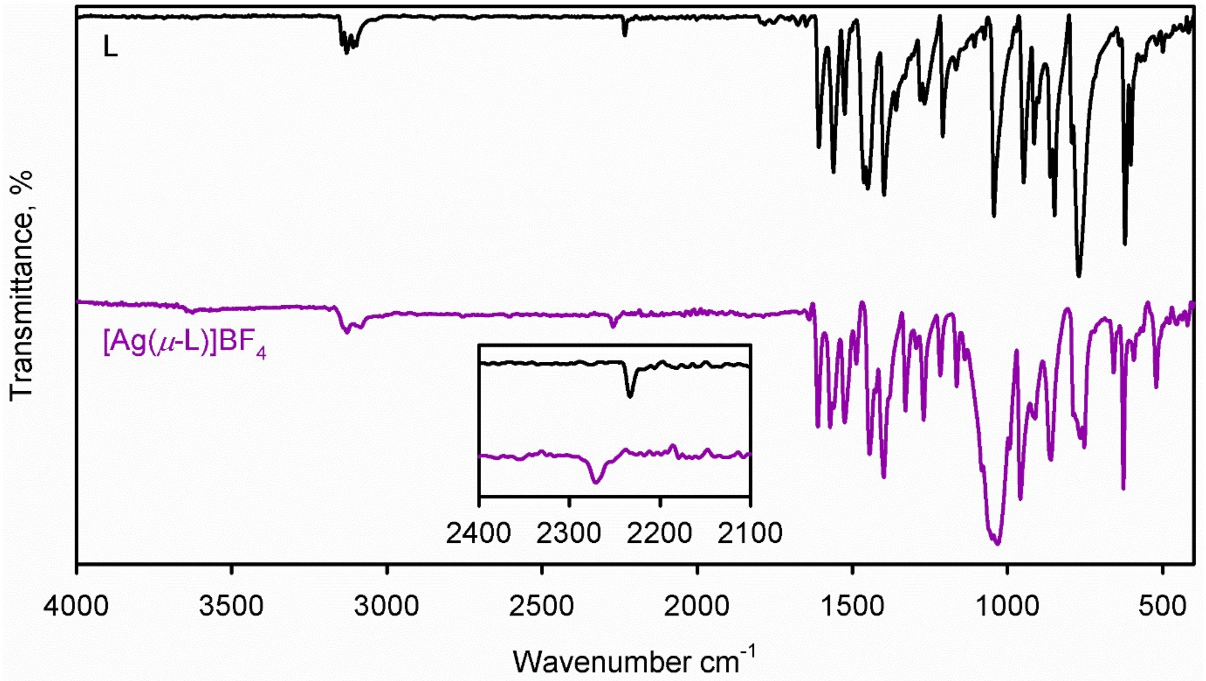

Figure S35 Comparison of the IR spectra of L (black) and [Ag(μ-L)]BF_4 (purple). The inset highlights the +38 cm^{-1} upward shift of the ν{C≡N} vibration in the silver coordination polymer, which is consistent with CN→Ag coordination.

References

[1] K. Senthil Kumar, N. Del Giudice, B. Heinrich, L. Douce, M. Ruben, *Dalton Trans.* **2020**, *49*, 14258–14267.

[2] G. M. Sheldrick, *Acta Cryst. Sect. A.: Found. Adv.* **2015**, *71*, 3–8.

[3] G. M. Sheldrick, *Acta Cryst. Sect. C.: Struct. Chem.* **2015**, *71*, 3–8.

[4] L. J. Barbour, *J. Appl. Cryst.* **2020**, *53*, 1141–1146.

[5] O. V. Dolomanov, L. J. Bourhis, R. J. Gildea, J. A. K. Howard, H. Puschmann, *J. Appl. Crystallogr.* **2009**, *42*, 339–341.

[6] C. J. O'Connor, *Prog. Inorg. Chem.* **1982**, *29*, 203–283.

[7] G. S. Pawley, *J. Appl. Crystallogr.* **1981**, *14*, 357–361.

[8] H. M. Rietveld, *J. Appl. Crystallogr.* **1969**, *2*, 65–71.

[9] D. R. Allan, S. P. Collins, G. Evans, D. Hall, K. McAuley, R. L. Owen, T. Sorensen, C. C. Tang, F. von Delft, A. Wagner, H. Wilhelm, *Eur. Phys. J. Plus* **2015**, *130*, 1–20.

[10] S. P. Thompson, J. E. Parker, J. Potter, T. P. Hill, A. Birt, T. M. Cobb, F. Yuan, C. C. Tang, *Rev. Sci. Instrum.* **2009**, *80*, 075107/1–9.

[11] S. P. Thompson, J. E. Parker, J. Marchal, J. Potter, A. Birt, F. Yuan, R. D. Fearn, A. R. Lennie, S. R. Street, C. C. Tang, *J. Synchrotron Radiat.* **2011**, *18*, 637–648.

[12] A. A. Coelho, *Topas Acad. Version 6*. http//www.topas-academic.net.

[13] A. A. Coelho, J. Evans, I. Evans, A. Kern, S. Parsons, *Powder Diffr.*, **2011**, *26*, S22–S25.

[14] A. A. Coelho, *J. Appl. Crystallogr.* **2018**, *51*, 210–218.

[15] J. M. Holland, J. A. McAllister, C. A. Kilner, M. Thornton-Pett, A. J. Bridgeman, M. A. Halcrow, *J. Chem. Soc. Dalton Trans.* **2002**, 548–554.

[16] M. A. Halcrow, *Coord. Chem. Rev.* **2009**, *253*, 2493–2514.

[17] L. J. Kershaw Cook, R. Mohammed, G. Sherborne, T. D. Roberts, S. Alvarez, M. A. Halcrow, *Coord. Chem. Rev.* **2015**, *289–290*, 2–12.

[18] I. Capel Berdiell, E. Michaels, O. Q. Munro, M. A. Halcrow, *Inorg. Chem*. **2024**, *63*, 2732–2744.

[19] L. J. Kershaw Cook, F. L. Thorp-Greenwood, T. P. Comyn, O. Cespedes, G. Chastanet, M. A. Halcrow, *Inorg. Chem.* **2015**, *54*, 6319−6330.

[20] I. Capel Berdiell, R. Kulmaczewski, N. Shahid, O. Cespedes, M. A. Halcrow, *Chem. Commun.* **2021**, *57*, 6566−6569.

[21] N. Suryadevara, A. Mizuno, L. Spieker, S. Salamon, S. Sleziona, A. Maas, E. Pollmann, B. Heinrich, M. Schleberger, H. Wende, S. K. Kuppusamy, M. Ruben, *Chem. − Eur. J.* **2022**, *28*, e202103853.

[22] R. Kulmaczewski, L. J. Kershaw Cook, C. M. Pask, O. Cespedes, M. A. Halcrow, *Cryst. Growth Des.* **2022**, *22*, 1960−1971.

[23] S. Vela, J. J. Novoa, J. Ribas-Arino, *Phys. Chem. Chem. Phys.* **2014**, *16*, 27012–27024.

[24] S. Brooker, *Chem. Soc. Rev.* **2015**, *44*, 2880–2892.

[25] A. J. Gordon, R. A. Ford, *The Chemists Companion – A Handbook of Practical Data, Techniques and References*, John Wiley, Chichester, UK, **1972**. p. 109.

[26] C. M. Pask, S. Greatorex, R. Kulmaczewski, A. Baldansuren, E. J. L. McInnes, F. Bamiduro, M. Yamada, N. Yoshinari, T. Konno, M. A. Halcrow, *Chem. – Eur. J.* **2020**, *26*, 4833–4841.

[27] E. Michaels, C. M. Pask, I. Capel Berdiell, H. B. Vasili, M. J. Howard, O. Cespedes, M. A. Halcrow, *Cryst. Growth Des.* **2022**, *22*, 6809−6817.

[28] L. J. Kershaw Cook, R. Kulmaczewski, R. Mohammed, S. Dudley, S. A. Barrett, M. A. Little, R. J. Deeth and M. A. Halcrow, *Angew. Chem. Int. Ed.* **2016**, *55*, 4327–4331.

[29] I. Capel Berdiell, V. García-López, M.J. Howard, M. Clemente-León, M. A. Halcrow, *Dalton Trans.* **2021**, *50*, 7417–7426.

[30] J. M. Holland, S. A. Barrett, C. A. Kilner, M. A. Halcrow, *Inorg. Chem. Commun.* **2002**, *5*, 328–332.

[31] I. Capel Berdiell, R. Kulmaczewski, S. L. Warriner, O. Cespedes, M. A. Halcrow, *Eur. J. Inorg. Chem.* **2020**, 4334–4340.
